# Supplementary material for: Antidyslipidemia Pharmacotherapy in Chronic Kidney Disease: A Systematic Review and Bayesian Network Meta-Analysis
Source: Pharmaceutics. 2022 Dec 20;15(1):6. doi: 10.3390/pharmaceutics15010006 (PMC9862001; doi:10.3390/pharmaceutics15010006)
Supplement: Supplementary file 1 [file pharmaceutics-15-00006-s001.zip › pharmaceutics-2029482-supplementary.pdf]

Supplementary Sections

Supplementary to:

Antidyslipidemia Pharmacotherapy in Chronic Kidney Disease: A Systematic Review and Bayesian Network Meta-analysis

Table of contents

S1. PRISMA flow diagram……………………………………………………………………...…4

S2. Words search strategy……………………………………………………………...….5

S3. Dose conversion of different statins……………………………………………….......7

S4. Baseline characteristics and major findings of studies………………………....... … ……..….....8

S5. Risk of bias in individual trials (Rob2)……………………………………………...…..28

S6. Supplemental information for all the outcomes of interest…………………………...….31

S6.1. All-cause death………………………………………..……………………………...31

S6.1.1. The funnel plot………………………………………..……………………………....31

S6.1.2. The analysis of consistency and heterogeneity………………..………………..……………………...32

S6.1.3. Trace plots, density plots, Brooks-Gelman-Rubin diagnosis plots and potential scale reduction factors……………………………………………..…………………………………....……………....34

S6.1.4. The rank probabilities of each treatment…...…………………..………………....……………......36

S6.1.5. The regression analysis for baseline LDL-c level……………………..…………….....…………......37

S6.1.6. Network meta-analysis with the frequentist model……………………..…………....…………......40

S6.1.7. Sensitivity analysis by excluding studies enrolling a large number of patients with cardiovascular disease……………….…………....……………………..……………………....……….........……….........…41

S6.2. The composite cardiovascular events………………..……………………………....………........42

S6.2.1. The definitions of the composite cardiovascular events in trials included in the NMA……………....……….........……………....……….................…………....……….........…………….......42

S6.2.2. The pairwise meta-analysis for the composite cardiovascular events……………………….……….45

S6.2.3. The funnel plot………………..…………………………………....……….........………......……...46

S6.2.4. The analysis of consistency and heterogeneity ………....……….......………….......................…....46

S6.2.5. Trace plots, density plots, Brooks-Gelman-Rubin diagnosis plots and potential scale reduction

factors……………..…………………………….....……….........………....……….....……………..…….…48

S6.2.6. The rank probabilities of each treatment.………........………....………......……….…..……. ….…...52

S6.2.7. The regression analysis for baseline LDL-c level.……….........………....……….....……..….….……...53

S6.2.8. Sensitivity analysis: network meta-analysis with the frequentist model………......……….…….....56

S6.2.9. Sensitivity analysis by excluding studies enrolling a large number of patients with cardiovascular disease ………........……….....……….....……..…..……. ….….........……….....……….....……..…..……...57

S6.3. Cardiovascular death and stroke……………………………..………………..………………..58

S6.4. LDL-c reduction…………………………..………………...………………..………..……..59

S6.4.1. The forest plot……......…………………….....…………....………...…………………....………………59

S6.4.2. The analysis of consistency and heterogeneity...……………..……......……………..………………..59

S6.5. Safety assessment…......………………………...…………...………...…………………...………………....... .61

S7. GRADE assessment………………...………….....………...………………...……………..……..……….....62

S8. The confidence assessment in the results of NMA using CINeMA approach……………………….......64

S8.1. The confidence assessment of all-cause death……………………………………………......……......64

S8.2. The composite cardiovascular events……………………………………………………......……......66

S8.3. Cardiovascular death……………………………………………………………………………........68

S8.4. Stroke…………………………………………………………………………………………......…70

S8.5. LDL-C reduction……………………………………………………………………..…......…72

S1. PRISMA flow diagram

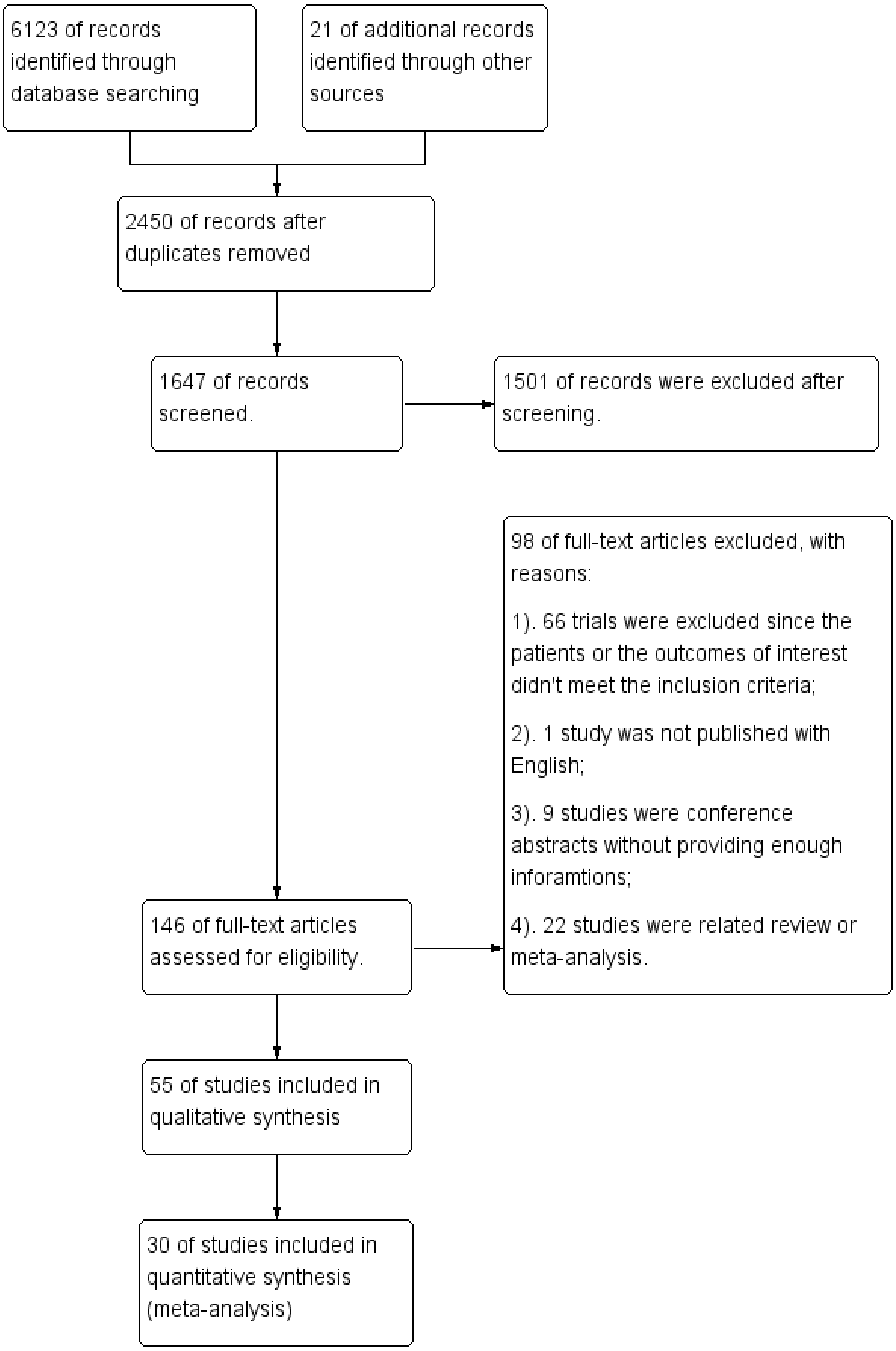

Search strategy using PRISMA statement. PRISMA: Preferred Reporting Items for Systematic Reviews and Meta-Analysis Extension.

S2. Words search strategy

Pubmed (1153 records)

#1 Chronic kidney insufficiency [MeSH Terms] 129255

#2 (((((kidney disease[Title/Abstract]) OR (renal disease[Title/Abstract])) OR (kidney failure[Title/Abstract])) OR (renal failure[Title/Abstract])) OR (kidney insufficiency[Title/Abstract])) OR (renal insufficiency[Title/Abstract]) 252599

#3 #1 OR #2 84215

#4 agents, lipid regulating [MeSH Terms] 61406

#5 ((((((((((((((((*statin[Title/Abstract]) OR (Evolocumab[Title/Abstract])) OR (Alirocumab[Title/Abstract])) OR (PCSK9[Title/Abstract])) OR (*fibrate[Title/Abstract])) OR (gemfibrozil[Title/Abstract])) OR (ezetimibe[Title/Abstract])) OR (fish oil[Title/Abstract])) OR (ω-3[Title/Abstract])) OR (ω-3[Title/Abstract])) OR (Polyunsaturated Fatty Acid[Title/Abstract])) OR (chlestyramine[Title/Abstract])) OR (colest*[Title/Abstract])) OR (bile acid sequestrant[Title/Abstract])) OR (niacin[Title/Abstract])) OR (acipimox[Title/Abstract])) OR (antioxidants[Title/Abstract])) OR (probucol[Title/Abstract]) 108666

#6 #4 OR #5 162761

#7 #3 AND #6 1153

Embase (567 records)

#1 'chronic kidney failure'/exp 184,288

#2 ('kidney disease':ti,ab OR 'renal disease':ti,ab OR 'kidney failure':ti,ab OR 'renal failure':ti,ab) AND 'kidney insufficiency':ti,ab OR 'renal insufficiency':ti,ab 33451

#3 #1 OR #2 209,576

#4 'hydroxymethylglutaryl coenzyme a reductase inhibitor'/exp OR 'hydroxymethylglutaryl coenzyme a reductase inhibitor' OR '?statin' OR '?fibrate' OR 'gemfibrozil'/exp OR 'gemfibrozil' OR 'ezetimibe'/exp OR 'ezetimibe' OR 'fibric acid derivative'/exp OR 'fibric acid derivative' OR 'pcsk9 inhibitor'/exp OR 'pcsk9 inhibitor' OR 'alirocumab'/exp OR 'alirocumab' OR 'evolocumab'/exp OR 'evolocumab' OR 'fish oil'/exp OR 'fish oil' OR 'ω-3' OR 'ω-6' OR 'chlest*' OR 'colest*' OR 'polyunsaturated fatty acid'/exp OR 'polyunsaturated fatty acid' OR 'bile acid sequestrant'/exp OR 'bile acid sequestrant' OR 'niacin'/exp

OR ‘niacin’ OR ‘acipimox’/exp OR ‘acipimox’ OR ‘antioxidant’/exp OR ‘antioxidant’ OR ‘probucol’/exp OR ‘probucol’ #5 ‘lipid’/exp/dm_dm,dm_dt 755,886

#5. ‘lipid’/exp/dm_dm,dm_dt 85,499

#6 #4 OR #5 823,711

#7 #3 AND #6 11,182

#8 ‘randomized controlled trial’/exp 723,775

#9 #7 AND #8 567

Cochrane library (4403 records)

#1 MeSH descriptor: [Renal Insufficiency, Chronic] explode all trees 7347

#2 (kidney disease):ti,ab,kw OR (renal disease):ti,ab,kw OR (kidney failure):ti,ab,kw OR (renal failure):ti,ab,kw 49446

#3 (kidney insufficiency):ti,ab,kw OR (renal insufficiency):ti,ab,kw 6797

#4 MeSH descriptor: [Hypolipidemic Agents] explode all trees 7033

#6 MeSH descriptor: [Hydroxymethylglutaryl-CoA Reductase Inhibitors] explode all trees 3716

#7 MeSH descriptor: [Ezetimibe] explode all trees 812

#8 MeSH descriptor: [PCSK9 Inhibitors] explode all trees 88

#9 MeSH descriptor: [Fibric Acids] explode all trees 1329

#10 MeSH descriptor: [Fish Oils] explode all trees 3774

#11 MeSH descriptor: [Fatty Acids, Unsaturated] explode all trees 13308

#12 MeSH descriptor: [Cholestyramine Resin] explode all trees 278

#13 MeSH descriptor: [Niacin] explode all trees 621

#14 MeSH descriptor: [Antioxidants] explode all trees 5214

#15 (statin):ti,ab,kw OR (ezetimibe):ti,ab,kw OR (fibrates):ti,ab,kw OR (PCSK9):ti,ab,kw OR

(colest*):ti,ab,kw9136

#16 (bile acid sequestrant):ti,ab,kw OR (acipimox):ti,ab,kw OR (Probucol):ti,ab,kw 589

#17 #4 OR #5 OR #6 OR #7 OR #8 OR #9 OR # 10 OR #11 OR #12 OR # 13 OR #14 OR #15 OR # 16 1241561

#18 #17 AND #3 4403

S3. Dose conversion in different statins

**Medications**	**Equivalent dose (mg)**				
Atorvastatin	-	10	20	40	80
Fluvastatin	40	80	-	-	-
Pitavastatin	1	2	4	-	-
Lovastatin	20	40-80	80	-	-
Pravastatin	20	40	80	-	-
Rosuvastatin	-	-	5	10	20
Simvastatin	10	20	40	80	-
LDL-c reduction (%)	30	38	41	47	55

**S4. Baseline characteristics and major findings of studies**		
**Study**	**Inclusion criteria**	**Major findings**
**PCSK9i**		
Tuñón2020: a prespecified analysis of ODYSSEY OUTCOMES trial	1) with an ACS 1–12 months before randomization;<br>2) having persistent dyslipidemia LDL-c ≥70 mg/dL, non-HDL-c≥100 mg/dL, or Apo B ≥80 mg/dL;<br>3) despite treatment with atorvastatin 40–80 mg daily, rosuvastatin 20–40 mg daily, or the maximum-tolerated dose of one of these statins;<br>4) an eGFR of <30 mL/min/1.73 m^2 at the screening visit for the study was an exclusion criterion.	**All-cause death and cardiovascular risk:**<br>1) all-cause death: in patients with recent ACS, alirocumab was associated with less death while the between-treatment difference was not significant in CKD persons;<br>2) cardiovascular events: this work did not find evidence for a greater absolute reduction of the composite cardiovascular events among CKD patients. While the low number of patients in the CKD subgroup limited power.
		**Renal function:** alirocumab or placebo did not influence eGFR at 36 months after randomization.
		**Lipid-lowering:** improve lipid profile significantly.
		**Adverse events:** the only adverse event with greater incidence in the alirocumab group was local injection site reactions.
Charytan2019:<br>Post hoc analysis of FOURIER 2017	1) patients with atherosclerotic cardiovascular disease and additional cardiovascular risk factors.<br>2) a fasting LDL-c level ≥70 mg/dl or non–HDL-c≥100 mg/dl.<br>3) on a high- or moderate-intensity statin (defined as equivalent to a dose of atorvastatin ≥20 mg daily).<br>4) patients with an eGFR <20 ml/min/1.73 m^2 or a history of renal transplantation were excluded.	**All-cause death and cardiovascular events:**<br>1) all-cause death: PS failed to decrease death effectively;<br>2) cardiovascular events: PS can decrease the composite cardiovascular events (cardiovascular death, myocardial infarction, or stroke) effectively.
		**Lipid-lowering:** PS improved lipid profile totally at each stage of CKD.
		**Renal function:** differences in eGFR between placebo and evolocumab-treated patients over time were minimal and nonsignificant.
		**Adverse events:** there was no evidence of an increase in the risk of adverse events in those taking evolocumab compared with those receiving placebo.
Toth2018:<br>Pooled analysis of 8	All trials had similar designs, with double-blind treatment periods of 6 to 26 months, and enrolled individuals	**All-cause death and Cardiovascular events:**<br>1) all-cause death: NA**;**

trials (LONG TERM, HIGH FH, FH I, FH II, COMBO I, COMBO II, OPTIONS I, OPTIONS II).	with hypercholesterolemia.	2) cardiovascular events: no significant difference in the occurrence of the composite cardiovascular events.<br><br>**Lipid-lowering:** regardless of baseline proteinuria status, LDL-c can be decreased effectively.<br><br>**Renal function and adverse events:** alirocumab was generally well tolerated and had no effect on renal function over the study period compared with control, regardless of baseline renal function status.<br><br>**Other important information:** hs-CRP levels were unchanged with alirocumab treatment, regardless of CKD status, indicating that this inflammatory marker of CVD risk is unaffected by alirocumab treatment among individuals with or without CKD.
Summary: PCSK9i can significantly improve lipid profile and composite cardiovascular events in CKD patients safely. No additional reduction in death and the composite cardiovascular events was observed with the further LDL-c decrease. However, this benefit was diminished with the deterioration of renal function, especially in patients with CKD stage≥3.		
**Fibrates**		
Dogra2007	1) patients with CKD stages 3 to 5.<br><br>2) exclusion criteria: nephrotic-range proteinuria (protein > 3 g/d), bilateral arteriovenous fistulas, abnormal liver function test results or muscle enzyme levels greater than 2 times the upper limit of normal, alcohol consumption greater than 30 g/d, active upper gastrointestinal dyspepsia, a clinical cardiovascular event including chest pain or angina within the preceding 6 months, use of anticoagulant or immunosuppressive therapy, and previous intolerance of statins or fibrates.	**Lipid-lowering:** gemfibrozil therapy was associated with a significant increase in HDL-c levels and a decrease in TG levels, but no significant changes in TC, LDL cholesterol, and oxidized LDL levels.<br><br>**Renal function:** within the predialysis group, there was no significant increase in SCr level.<br><br>**Adverse events:** compliance with gemfibrozil, was lower than with placebo. There was a greater incidence of self-reported gastrointestinal side effects (nausea, bloating, and diarrhea) with gemfibrozil. No significant episodes of myalgia, myositis, or CK or ALT level increase with atorvastatin or gemfibrozil were observed.
Weinstein2013	1) stage 3 CKD.<br><br>2) mixed dyslipidemia (fasting TG ≥150 mg/dL; LDL-c ≥130 mg/dL; and HDL-c <40 mg/dL for men and <50 mg/dL for	**Lipid-lowering:** combination therapy with fenofibric acid plus rosuvastatin resulted in significantly greater decreases in TG, HDL-c, and non-HDL-c, compared with rosuvastatin monotherapy. Prespecified subgroup analyses

	women).	showed no significant treatment-by-subgroup interaction for age (<65 vs ≥65 years).<br><br>**Adverse events:** few patients experienced muscle-, renal-, or hepatic-related adverse events of special interest.<br><br>**Renal function:** decreased eGFR occurred more frequently in the combination therapy group than in the rosuvastatin monotherapy group_(8.6% vs 1.4%; P ≤ 0.05), but was reversible.
Ting2012: FIELD study	1) patients with type 2 diabetes, aged 50–75 years.<br><br>2) exclusion criteria: plasma creatinine>130 mmol/L, liver or symptomatic gallbladder disease, or a CVD event within 3 months before recruitment.	**All-cause death and cardiovascular risk:**<br><br>1) all-cause death: total mortality was not decreased significantly;<br><br>2) cardiovascular events: fenofibrate group had independently significant absolute risk reductions in coronary revascularization, total CVD events, and CVD mortality, but not in coronary events, or total stroke.<br><br>**Renal function**: the use of a standard dose of fenofibrate in patients with moderate renal impairment had no adverse effects on either CVD or renal outcomes.<br><br>**Adverse events**: adverse event rates generally were no higher among those receiving fenofibrate than those on placebo.
Tonelli2004:<br><br>VA-HIT 1999	1) a documented history of CAD (previous MI, angina corroborated by objective evidence of ischemia, coronary revascularization, or angiographic evidence of stenosis>50% of the luminal diameter in>1 major epicardial coronary artery), age<74 years.<br><br>2) HDL-c level of 40 mg/dL or less(≤1.0 mmol/L), LDL-c level of 140 mg/dL or less (≤ 3.6 mmol/L), and TG level of 300 mg/dL or less (≤ 3.4 mmol/L).<br><br>3) participants with SCr value greater than 2.0 mg/dL (>176 mol/L) were excluded.	**All-cause and cardiovascular event:**<br><br>1) all-cause death: NA.<br><br>**2)** cardiovascular events: gemfibrozil reduced the cumulative incidence of CV death or MI, and also appeared to reduce the risk of coronary revascularization, although this was not statistically significant.<br><br>**Lipid-lowering:** gemfibrozil was associated with statistically significant reductions of TG, TC, and a significant increase of HDL-c compared with placebo, without an obvious effect on mean LDL-c levels.<br><br>**Renal function:** the rate of change in renal function in the gemfibrozil group was not significantly different from that in the placebo group.
Samuelsson1997	With non-diabetic primary renal disease and moderately advanced renal	**Lipid-lowering:** gemfibrozil resulted in significantly reduced concentrations of plasma lipids in TC (13%), TG(47%) and LDL-c(14%),

	insufficiency.	and apo B.
		**Renal function:** the change in eGFR didn't differ in the two groups.
Summary: fibrates can significantly decrease the plasma TG concentration but not LDL-c in CKD, which is consistent in non-CKD individuals. RCTs indicated that it may be associated with the reduction of cardiovascular events, especially CV death, without a significant effect on the decrease of all-cause mortality. Most CKD patients can tolerate fibrate without serious or frequent drug-related Aes. Although it may increase SCr in a short time, particularly when it was added to the use of statin, this adverse effect on renal function is reversible. To conclude, fibrates can be applied in the therapy of CKD patients as an ancillary lipid-lowering drug.		
**High-intensity statins**		
Holme2009:<br>IDEAL 2005	Patients with a history of confirmed MI.	**All-cause and cardiovascular events:**<br>1) all-cause death: no significant reduction.<br>2) cardiovascular events: compared with LS, HS significantly reduced the risk of any cardiovascular event and stroke but not major coronary events and MI.
		**Lipid-lowering:** HS significantly improves the lipid profile.
		**Renal function:** kidney function in the CKD group improved during the study by statin treatment and HS appeared to maintain eGFR during the 5-year treatment period significantly better than simvastatin.
Ridker2010:<br>A secondary analysis from the JUPITER JUPITER 2008	1) CKD men and women free of cardiovascular disease.<br>2) had LDL-c<130 mg/dl and high sensitivity C-reactive protein (hs-CRP) ≥2 mg/l.<br>1) exclusion criteria: treatment within 6 weeks of screening with any lipid-lowering therapies, current use of hormone replacement therapy, evidence of hepatic dysfunction, creatinine>2.0 mg/dl, diabetes, uncontrolled hypertension, prior malignancy, uncontrolled hypothyroidism, or a recent history of alcohol, drug abuse, or other medical condition that might compromise safety.	**All-cause death and cardiovascular events**:<br>1) all-cause death: rosuvastatin could significantly reduce all-cause death;<br>2) cardiovascular events: MI, stroke, hospital stay for unstable angina, revascularization, or confirmed CV death were decreased obviously.
		**Lipid-lowering**: HS significantly improves lipid profile especially LDL-c, and CRP.
		**Renal function**: there is no evidence in the JUPITER trial that rosuvastatin led to any impairment of renal function as measured by eGFR at the 12-month visit; if anything, in the trial as a whole, median eGFR at 12 months was marginally improved among those randomly allocated to rosuvastatin as compared with placebo.
		**Adverse events**: adverse event rates associated with treatment were similar between rosuvastatin

		and placebo groups among those with CKD.
Koren2009: post hoc analysis of ALLIANCE2004	Older than 18 years with known CHD, defined as prior ACS, PCI, CABG.	**All-cause death and cardiovascular events:**<br>1) all-cause death: all-cause mortality was reduced significantly compared with usual care;<br>2) cardiovascular events: atorvastatin therapy reduced the relative risk of cardiovascular events. A trend toward benefit with atorvastatin was observed for all secondary end points (peripheral revascularization, hospitalization for congestive heart failure, and stroke) in patients with CKD. The benefit of atorvastatin therapy appeared greater in patients with CKD than in patients without CKD.
		**Lipid-lowering:** mean baseline TG was greater in patients with CKD. Significant decreases in LDL-c levels were seen in the atorvastatin versus usual-care group irrespective of whether patients had or did not have CKD. The decrease in TG levels in each renal cohort with atorvastatin therapy did not reach statistical significance versus usual care.
		**Renal function:** the difference in eGFR between the two groups didn't reach statistical significance in CKD patients.
		**Adverse events**: few treatment-related adverse events in the two groups.
Shepherd2008:<br>(The TNT Study)	Ages 35 to 75 years with clinically evident CHD, defined as previous MI, previous or current angina with objective evidence of atherosclerotic CHD, or a history of coronary revascularization.	**All-cause death Cardiovascular events**:<br>1) all-cause death: no significant effect;<br>2) cardiovascular events: atorvastatin 80 mg significantly reduced the incidence of major cardiovascular events. They observed a significantly greater treatment effect in patients with CKD than in patients with normal eGFR.
		**Lipid-lowering**: patients with CKD had a higher TG level at baseline. Mean LDL-c and TG levels at the final visit were similar in the two groups. HDL-c levels remained stable with no differences between treatment groups.
		**Adverse events**: safety of atorvastatin 80 mg in patients with CKD was similar to that reported for the overall TNT population, with no

		unexpected safety concerns identified.
Deedwania2015	Participants were aged 65–85 years with a documented history of clinically stable CAD and one or more episodes of myocardial ischemia with a total ischemia duration ≥3 min during 48-h ambulatory ECG at screening and with baseline low-density lipoprotein cholesterol (LDLC) 100–250 mg/dL.	**Renal function:** this post hoc analysis observed the renal benefits of statins to a high-risk population of older patients with a history of stable CAD with silent ischemia, in particular to those with coexistent CKD.
Summary: for CKD patients, HS can improve lipid profile, resulting in more reduction in LDL-c without obvious TG lowering compared with LS. And the relationship between lipid-lowering and cardiovascular risk reduction is not well-defined and controversial. Compared with placebo or LS, HS is associated with a significant decrease in CV risk and all-cause mortality even in those without primary CHD. According to current studies, there is no evidence supporting the positive or negative influence of high-intensity statins on renal function and CKD progression. Meanwhile, no serious and obvious treatment-related events were observed in these patients, suggesting the safety of high-intensity statin. However, the absence of dialysis ones requires further study on the effect and safety.		
**Ezetimibe**		
Stanifer2017:<br><br>Post hoc analysis of IMPROVE-IT 2015	1) adults ≥50 years.<br>2) had been hospitalized within the preceding 10 days for ACS.<br>3) have an LDL-c of 1.3 mmol/L or higher.<br>4) exclusion criterion: creatinine clearance (CrCl)<30 ml/min.	**All-cause death and cardiovascular events:**<br>1) all-death death: negative effect;<br>2) cardiovascular events: the difference in treatment was statistically significant and the benefit of combination therapy on cardiovascular events reduction was most pronounced at eGFR levels<60 ml/min per 1.73 m^2, indicating that the combination therapy can result in additional benefit for the secondary prevention in CKD patients.
		**Lipid-lowering**: For CKD patients, individuals in the combination therapy arm experienced a greater mean change in LDL-c (P=0.01), TG (P,0.001), and HDL-c(P=0.03) compared with individuals in the monotherapy arm at 1 year of follow-up.
		**Adverse events:** few adverse events in either treatment were observed.
Suzuki2013	1) age 35 to years.<br>2) undergoing treatment with low-dose statins.<br>3) LDL cholesterol >120 mg/dl.<br>4) and positive proteinuria or eGFR	**Cardiovascular events:** during the study, no atherosclerotic events were reported in either group.
		**Lipid-lowering:** the proportional decreases in LDL-c at 1 year in the two groups appeared similar among patients with CKD 1 and 2 and

	<60 ml/min/1.73 m2 for more than 3 months before enrollment.<br>5) exclusion criterion: undergoing dialysis therapy; uncontrolled hypertension; uncontrolled diabetes; severe liver disease with alanine transaminase (ALT) levels >2 times the upper limit of normal (ULN); TG >400 mg/dl; secondary hyperlipidemia or hyperlipidemia associated with the administration of a drug; homozygous familial hypercholesterolemia; unstable angina, MI, surgical coronary intervention or stroke within 3 months of study entry; pregnancy, possible pregnancy, desire to become pregnant during the study period, or lactation; history of hypersensitivity to any ingredient in ezetimibe tablets; and deemed inappropriate for study entry by the investigator.	patients with CKD 3–5.<br>**Adverse events:** among patients with CKD stages 3–5, no difference in the occurrence of muscle-related events, and combination therapy was associated with fewer events of liver-related toxicity.<br>**Renal function:** the two groups did not differ in the change in renal function.
Baigent2011,<br>(SHARP 2011)<br>Haynes2014 [1]<br>The post hoc analysis of SHARP 2011	1) patients aged 40 years and older.<br>2) had plasma creatinine of at least 150umol/L (1.7 mg/dL) in men or 130 umol/L (1.5 mg/dL) in women, whether receiving dialysis or not.	**All-cause death and cardiovascular events:**<br>1) all-cause death: no significant difference;<br>2) cardiovascular events: reduced the incidence of major atherosclerotic events in a wide range of patients with advanced CKD.<br>**Lipid-lowering:** allocation to simvastatin plus ezetimibe reduced LDL-c by an average of 0.85 mmol/L over about 5 years, compared with placebo.<br>**Renal function:** exploratory analysis showed no significant effect on the rate of change in eGFR among patients on dialysis or not on dialysis.<br>**Adverse events:** there were very few cases of myopathy of any severity or more severe cases with rhabdomyolysis. Nor were there significant excesses of persistently raised transaminases to greater than three times the upper limit of normal value, hepatitis, or gallstones.
Hagiwara2017:<br>HIJ-PROPER 2017	All participants≥had been hospitalized for STEMI or NSTEMI or UA within 72 h before randomization.	**All-cause death and cardiovascular events:**<br>No significant difference was noted between standard and intensive treatment in terms of the primary outcome including all-cause death, non-fatal MI, non-fatal stroke, unstable angina, and ischemia-driven revascularization among

		patients with eGFR<60ml/min/1.73m^2.
		**Lipid-lowering**: combination therapy reduced LDL-c significantly throughout the study.
		**Renal function:** no significant between-group differences were seen in the percentage of patients who achieved creatine kinase elevation ≥5 the upper limit of normal value or doubling of SCr.
		**Adverse events:** no significant between-group differences were seen.
Landray2006：<br>A pilot study:<br>Biochemical safety and efficacy of adding ezetimibe to simvastatin as initial therapy among patients with advanced CKD.	1) men or women aged≥18 years.<br>2) they were a predialysis patient with the most recent serum or plasma creatinine level of 1.7 mg/dL or greater, a hemodialysis patient, or a peritoneal dialysis patient.<br>3) their nephrologist did not consider that there was a definite indication for cholesterol-lowering therapy or a definite contraindication to either simvastatin or ezetimibe therapy.<br>4) exclusion criteria: history of acute uremic emergency within the preceding 2 months; history of chronic liver disease or baseline alanine transaminase [ALT] level 1.5 or more times the upper limit of normal (ULN); active muscle disease, or creatine kinase [CK] level greater than 3 times the ULN; previous adverse reaction to a statin or ezetimibe; current treatment with a contraindicated drug; childbearing potential in the absence of a reliable method of contraception; a life-threatening condition other than chronic renal failure or vascular disease; frequent nonattendance at clinics, or known noncompliance with drug treatments.	**Lipid-lowering:** the addition of ezetimibe to simvastatin as initial therapy showed an incremental significant decrease of LDL-c.
		**Adverse events:** no serious adverse event was attributed to the study's treatment.
		**Renal function:** no significant reduction of eGFR was observed during the 6-month follow-up in the two treatment groups.
Summary: compared with statin monotherapy, the combination of statin and ezetimibe can improve lipid profile, including reducing TG, TC, LDL-c, and increasing HDL-c. But the additional benefit of this combination therapy was not obvious and controversial compared with statin up-titration. For the cardiovascular outcomes, the majority of trials failed to observe the obvious benefit of combination therapy on risk reduction. Combination therapy and statin monotherapy were both safe.		
**Low-intensity statins**		

Wanner2005: 4D RCT<br><br>März2011: the post hoc analysis of 4D RCT	1) subjects with type 2 diabetes mellitus 18 to 80 years of age.<br>2) had been receiving maintenance hemodialysis for less than two years.<br>3) exclusion criteria: levels of fasting serum LDL-c of less than 2.1 mmol/L or more than 4.9 mmol/L, TG levels greater than 11.3 mmol/L; liver function values more than three times the upper limit of normal or equal to those in patients with symptomatic hepatobiliary cholestatic disease; hematopoietic disease or systemic disease unrelated to end-stage renal disease; vascular intervention, congestive heart failure, or MI within the three months preceding the period of enrollment; unsuccessful kidney transplantation; and hypertension resistant to therapy.	**All-cause death and cardiovascular events：**<br>1) all-cause death: no significant effect.<br>2) cardiovascular events: atorvastatin had no statistically significant effect on the composite primary end point of cardiovascular death, nonfatal myocardial infarction, and stroke in patients with diabetes receiving hemodialysis. While in the subgroup analysis, atorvastatin significantly decreased adverse fatal and nonfatal cardiac events and all-cause death compared with placebo at a baseline LDL-c>145 mg/dl with no significant effect of atorvastatin below this threshold.
		**Lipid-lowering**: after four weeks of treatment, the median level of LDL-c was reduced by 42 percent among patients receiving atorvastatin, and among those receiving placebo it was reduced by 1.3 percent.
		**Adverse events:** patients receiving hemodialysis generally have many adverse and serious adverse events, but no cases of rhabdomyolysis or severe liver disease were detected in either group.
Bianchi2003	Patients with mild to moderate CKD, proteinuria, and hypercholesterolemia were enrolled in this study, CrCl (ml/min) was about 50ml/min.	**All-cause death and cardiovascular events:** NA
		**Lipid-lowering**: during this time, TC and LDL-c decreased in patients treated with atorvastatin, but did not change in patients treated with conventional therapy.
		**Adverse events**: NA
		**Renal function**: by the end of one-year treatment, urine protein excretion can be decreased by atorvastatin in addition to ACE inhibitor or ARBs while creatinine clearance decreased only slightly.
Fassett2010	1) prior use of ACEIs or ARBs.<br>2) age between 20-49 and 50-80 years.	**Renal function**: compared with placebo, active treatment was associated with an attenuated increase in urinary albumin excretion over time and an attenuated increase in urinary albumin excretion over time (P=0.04 for between-group difference after 24 months), but no effect was observed on renal function.

Kendrick2010: post hoc analysis of AFCAPS/TexCAPS	1) TC level of 180-264 mg/dL, LDL-c level of 130-190 mg/dL, HDL-c level ≤ 45 mg/dL for men or ≤ 47 mg/dL for women, and TG level ≤ 400 mg/dl.<br>2) exclusion criteria: clinical evidence of atherosclerotic CVD, secondary hyperlipoproteinemia, nephrotic syndrome, uncontrolled hypertension, and type 1 or 2 diabetes mellitus.	**All-cause death and cardiovascular events:**<br>1) all-cause death: NA;<br>2) cardiovascular events: after an average follow-up of 5.3±0.8 years, the incidence of a fatal and nonfatal CVD event was lower in participants with CKD receiving lovastatin than in those receiving a placebo.
		**Renal function:** lovastatin did not have renoprotective effects in CKD without prior CVD.
Tonelli2005:<br>Pooled analysis of CARE, LIPID, WOSCOPS	High-risk individuals who had not previously experienced a MI.	**All-cause death and cardiovascular events:**<br>1) all-cause death: pravastatin didn't significantly reduce all-cause mortality;<br>2) cardiovascular events: this analysis confirms that statins reduce cardiovascular risk in diabetic individuals with mild or moderate CKD.
		**Lipid-lowering:** pravastatin reduced LDL-c and TG and raised HDL-c significantly at 12 months, compared with baseline. LDL-c was reduced by a significantly greater extent in subjects with more severe renal insufficiency.
		**Renal function:** in the CARE trial (one of the trials included), pravastatin significantly reduced rates of decline in renal function in individuals with MDRD-GFR<40 ml/min per 1.73 m^2 and in those with MDRD-GFR<50ml/min per 1.73 m^2 in association with proteinuria. These data suggest that individuals with moderate to severe kidney disease may derive clinically relevant renal benefits from the use of pravastatin, especially those with proteinuria.
Verma2005:	1) age>18 years with no clinical evidence of acute renal failure.<br>2) not undergoing hemodialysis, not recently hospitalized for any reason, no known recent acute illness or infection, not currently taking antilipemic medications, no history of statin therapy discontinuation because of adverse events, no	**All-cause death and cardiovascular events:** NA.
		**Lipid-lowering**: patients receiving rosuvastatin tended to derive more favorable improvements from baseline values in LDL-c.
		**Renal function:** eGFR increased significantly with 20 weeks of rosuvastatin treatment. In contrast, eGFR did not change significantly with

	known contraindications to statin therapy.<br>3) fasting LDL-c≥100 mg/dl, HDL-c<40 mg/dl, and/or TG≥150 mg/dl.<br>4) the ability to attend the study clinic once every 4 weeks for 20 weeks.	no antilipemic treatment.<br><br>**Adverse events**: no patients in either group experienced elevations in serum ALT or AST to≥3 times the upper limit of normal or elevation in serum creatine kinase to ≥10 times the upper limit of normal during the study.
Goicoechea2006	1) with an eGFR<90 ml/min and>15 ml/min.<br>2) exclusion criteria: levels of fasting LDL-c of 100 mg/dl and 200 mg/dl; the existence of liver, cardiovascular, infectious, or systemic disease unrelated to ESRD; and the history of vascular intervention, congestive heart failure, or MI within the 3 mo preceding the period of enrollment. No patient took lipid-lowering medication during the 4-wk period before the study.	**All-cause death and cardiovascular events:** NA<br><br>**Lipid-lowering:** compared with no antilipemic treatment, LDL-c, TC, and TG were improved but significant improvement in HDL-c was not observed.<br><br>**Renal function:** no significant change in eGFR was not observed in the two groups.
Colhoun2009:<br>CARDS 2009	1) patients had to have diabetes and at least 1 of the following risk factors: history of hypertension, retinopathy, microalbuminuria or macroalbuminuria, or current smoking.<br>2) exclusion criteria: had a history of MI, angina, coronary vascular surgery, cerebrovascular accident, or severe peripheral vascular disease; had a plasma creatinine concentration greater than 1.7 mg/dL or glycated hemoglobin level greater than 12%. Mean serum LDL-c concentration during baseline visits before randomization had to be 160 mg/dL or less (4.14 mmol/L), and serum triglycerides, 600 mg/dL or less (6.78 mmol/L).	**All-cause death and cardiovascular events:**<br>1) all-cause death: no significant difference;<br>2) cardiovascular events: use of statins in the majority of patients with diabetes delivers substantial macrovascular benefits, even in those with modest impairment in kidney function.<br><br>**Lipid-lowering:** average absolute decreases in LDL-c and TC with atorvastatin were very slightly, but significantly, greater in those with eGFR less than 60 mL/min/1.73 m^2 compared with those with eGFR greater than this level.<br><br>**Renal function:** the beneficial effect on eGFR was modest and of uncertain long-term clinical significance, but it suggests that longer-term statin use in albuminuric patients with diabetes might have some clinically beneficial preventive effect on the decrease in kidney function.
Kimura2016:<br>(ASUCA 2016**)**	1) 40<age<75.<br>2) not treated with statins.	**All-cause death and cardiovascular events:**<br>1) all-cause death: no significant effect;

	3) LDL-c ≥140 mg/dl in subjects not taking any dyslipidemia-treating agents or LDL-c ≥100 mg/dl in those taking dyslipidemia-treating agents other than statins.<br>4) exclusion criteria: eGFR<30 ml/min/1.73 m2; systolic blood pressure ≥180 mmHg or diastolic blood pressure C110 mmHg; HbA1c>8.5%; familial hypercholesterolemia; secondary hypercholesterolemia including nephrotic syndrome; liver dysfunction including acute hepatitis, chronic active hepatitis, liver cirrhosis, and hepatoma; history of severe side effects of atorvastatin; pregnancy, the possibility of pregnancy, or breast-feeding woman.	2) cardiovascular events: no significant effect.<br><br>**Lipid-lowering:** LDL-c, and TG decreased significantly in atorvastatin. The difference in the HDL-c lowering effects between the two groups did not reach statistical significance.<br><br>**Renal function**: The difference in mean eGFR changes between the two groups didn't reach statistical significance.
Lemos2005[2]:<br>a LIPS sub-study<br>.	1) TC level of 135 to 270 mg/dl with a fasting TG level<400 mg/dl.<br>2) TC level<212 mg/dl for patients whose lipids levels were measured 24 hours to 4 weeks after an episode of MI.<br>3) TC level<232 mg/dl for patients who had diabetes mellitus.<br>4) exclusion criteria included a baseline SCr value<1.8 mg/dl.	**All-cause death and cardiovascular events:**<br>**1)** all-cause death: NA<br>**2)** cardiovascular events: renal impairment increases the incidence of atherosclerotic-related events after vascular intervention treatment and this effect could be abolished by long-term fluvastatin therapy, which could not be explained by a differential action on lipid levels or renal function during follow-up.<br><br>**Lipid-lowering**: the decrease of LDL-c was similar in patients who had renal impairment and those who did not. At the end of the study, no significant differences in TG levels were observed between treatment groups. Levels of HDL-c increased by a median of 12%, regardless of treatment allocation or baseline renal function.<br><br>**Renal function**: renal function remained stable throughout follow-up and the predicted clearance-time profile was not influenced by fluvastatin therapy, regardless of baseline creatinine clearance.
Stegmayr2005	1) CKD patients with age<18 years.<br>2) exclusion criteria: fertile women who were not taking oral contraceptives; pregnant or lactating women; patients with active liver disease; a history of adverse reactions to statins; patients	**All-cause death and cardiovascular events:** atorvastatin had no apparent benefit concerning the primary endpoints of overall mortality or cardiovascular events.<br><br>**Lipid-lowering:** the treatment group showed considerable reduction in their LDL-c levels.

	with a functioning kidney transplant who were not on dialysis; patients on the waiting list for transplantation; those on a protein-restricted diet of<40 g of protein/day; and patients with poor compliance to medication and follow-up; patients with a history of a progressive malignancy and those with a life expectancy of<6 months.	**Adverse events:** there were no significant differences in the mean levels of safety variables (ASAT, ALAT, GT, CK) between Groups A and C, and a change did not occur over time.
Harris2001	1) aged 18 years or older and had chronic renal failure treated with dialysis for at least three months.<br>2) had untreated dyslipidemia [defined as total cholesterol ≥5.2 mmol/L (200 mg/dL) and LDL-cholesterol≥3.5 mmol/L (135 mg/dL)] or dyslipidemia uncontrolled by treatment with maximally tolerated doses of other lipid-lowering agents taken for a minimum period of four weeks.<br>3) exclusion criteria: included ALT, AST, creatinine kinase≥3ULN; patients who had a MI, revascularization within 3 months.	**Lipid-lowering:** after four weeks, a significantly greater proportion of patients receiving atorvastatin 10 mg had achieved the LDL-c goal of ≤3.5 mmol/L compared with patients receiving a placebo. The statistically significant difference between the two groups was maintained at week 8 and week 16 (P≤0.001 at both time points).<br>**Adverse events:** no differences between atorvastatin and placebo in the overall incidence or severity of serious adverse events.
Nakamura2008.<br>MEGA 2006	1) aged 40–70 years with total cholesterol (TC) levels of 5.69–6.98 mmol/L (220–270 mg/dL) and no history of CHD and/or stroke.<br>2) major exclusion criteria: familial hypercholesterolemia, history of CVD, cancer, severe renal dysfunction (SCr≥ 1.5 mg/dL), significant liver disease, and secondary hyperlipidemia.	**All-cause death and cardiovascular events:**<br>1) all-cause death: the addition of statin to diet therapy significantly reduce all-cause mortality.<br>2) cardiovascular events: CHD, stroke, and CVD, were significantly reduced. (a marked risk reduction in patients with moderate CKD compared with the total group was found, confirming the beneficial effect of pravastatin in individuals with moderate

		CKD.)
		**Lipid-lowering:** among patients assigned diet plus pravastatin LDL-c was significantly reduced.
		**Adverse events:** in patients with moderate CKD, there were no significant differences in serious adverse events or subjective complaints of side effects between the two treatment arms.
		**Renal function:** The change in eGFR during follow-up in patients with moderate CKD was significantly higher in those assigned to receive diet plus pravastatin compared with those on diet alone. Pravastatin also exerted beneficial effects on renal function in patients with moderate CKD.
Rahman2008<br>(post hoc of ALLHAT)	1) age≥55 years and stage 1 or 2 hypertension with at least 1 additional CHD risk factor.<br>2) fasting LDL-c level of 120 to 189 mg/dL (3.1 to 4.9 mmol/L) for those with no known CHD or 100 to 129 mg/dL (2.6 to 3.3 mmol/L) for those with known CHD, and fasting triglyceride level less than 350 mg/dL(<3.9 mmol/L). Participants excluded who were currently using prescribed lipid-lowering agents or large doses (≥500 mg/d) of nonprescription niacin, were known to be intolerant of statins or to have significant liver dysfunction (serum alanine aminotransferase>100IU/L), had other contraindications for statin therapy, or had a known secondary cause of hyperlipidemia.	**Lipid-lowering:** LDL-c, HDL-c, and TG measurements were available for only a small subset of patients, LDL-c was decreased significantly while there were no statistically significant differences between the pravastatin and usual-care groups concerning change in HDL-c or TG between baseline and year 6.
		**Renal function:** there were no statistically significant differences in rates of change in eGFRs between pravastatin and usual care in the overall population and stratified by baseline eGFR. But there was a trend for a greater eGFR in the pravastatin group.
Fellström2009:<br>(AFCAPS)	1) men and women 50 to 80 years of age who had ESRD and had been treated with regular hemodialysis or hemofiltration for at least 3 months.<br>2) exclusion criteria: statin therapy within the previous 6 months, expected kidney transplantation within 1 year, and serious hematologic, neoplastic, gastrointestinal, infectious, or metabolic disease (excluding diabetes) that was predicted to limit life expectancy to less than 1 year. A history of a malignant condition,	**All-cause death and cardiovascular events:**<br>1) all-cause death: there was no significant effect of rosuvastatin on all-cause mortality<br>2) cardiovascular events: no effect of rosuvastatin on the primary end point of nonfatal MI, nonfatal stroke, or CV death.
		**Lipid-lowering:** the lipid profile was generally improved.
		**Adverse events**: there was a high incidence of adverse and serious adverse events in these HD patients, without significant differences between

	active liver disease, uncontrolled hypothyroidism, and an unexplained elevation in the creatine kinase level to more than three times the upper limit of the normal range.	treatment groups.
Burmeister2009	1) had been regularly treated with 4-hour HD sessions 3 times a week for the last 3 months or more.<br>2) on regular hemodialysis, irrespective of their levels of LDL-c.<br>3) exclusion criteria: included uncontrolled diabetes mellitus, levels of fasting LDL-c of >190 mg/dL, TG levels greater than 400 mg/dL, impaired hepatic function, elevated SCr phosphokinase levels, use of beta-blockers, any active infectious disease, past or present malignancies, acute MI, or any other systemic disease not-related to CRF, and previous usage of any lipid-lowering drug for the last 3 months. Any concurrent acute inflammatory disease during the follow-up led to the discontinuation of the patient's participation in the study.	**Lipid-lowering:** daily administration of 10 mg of rosuvastatin significantly decreased total cholesterol, LDL-c, and non-HDL-c levels after 3 months when compared with the baseline. The hs-CRP level was significantly lower at the end of the study when compared with that of the placebo group, but not significantly different compared with the baseline.<br>**Adverse events**: few adverse events occurred in two groups.
Holmberg2005	1) eGFR<30ml/min/1.73m^2.<br>2) exclusion criteria: age<18 years; fertile women who were not using contraception; pregnant or nursing women; patients with active liver disease and a history of adverse reactions to statins; patients with a functioning kidney transplant; patients who were on a waiting list for kidney transplantation; patients on a protein-restricted diet (<40 g); and patient with a history of bad compliance with medication or follow-up. Patients with a history of progressive neoplastic disease as well as those expected to live for B6 mo< were also excluded.	**Lipid-lowering**: atorvastatin 10 mg/day effectively lowers LDL-c, TC and, to some extent, TG. The efficacy of atorvastatin in reducing LDL-c did not differ in percentage terms depending on whether the baseline LDL concentration was high or low. This indicates that the same doses of atorvastatin can be used for patients with higher and lower baseline concentrations of LDL-c.<br>**Adverse events**: a considerable number of patients withdrew from the study due to Aes, and no severe Aes occurred. The commonest Aes were nausea, vomiting, headache, and gastrointestinal problems. However, when atorvastatin is used in severe CKD patients, care should be taken regarding the development of AEs, which is not always reflected by laboratory values.
Yasuda2004	1) 18-70 years old and had at least 1	**Lipid-lowering**: LDL-c was reduced significantly by fluvastatin while no significant

	month on CAPD.<br>2) exclusion criteria: inflammatory cause of ESRD, hepatic or malignant disease, any infectious disease within the last 3 months, drug intake with anti-inflammatory effect (including statins, steroids, or NSAIDs), or uncontrolled cholesterol levels≥400 mg/dL.	difference in serum concentrations of TG, HDL-c, and apo-lipoproteins A-I and E were observed between the two groups.<br><br>**Adverse events:** the serum myoglobin concentration, serum creatine kinase, and aldolase levels did not change during treatment in both treatment groups.<br><br>**Renal function**: no significant difference in SCr concentrations and creatinine clearance values was observed between the two groups throughout the 48-week treatment period. Similarly, 24-hour urinary albumin excretion rates did not change significantly throughout treatment in both groups.
Chonchol2004: 4S RCT	1) aged 35 to 70 years with a history of MI and/or angina.<br>2) TC levels of 212 to 309 mg/dL and TG levels less than 221 mg/dl were randomly assigned to treatment with simvastatin, 20 mg/d, or placebo and followed up for a median of 5.4 years.<br>3) exclusion criteria: patients were excluded if they had secondary hypercholesterolemia, unstable angina, planned coronary artery surgery or angioplasty, recent MI, congestive heart failure requiring treatment, or hypersensitivity to 3-hydroxy-3-methylglutaryl coenzyme A reductase inhibitors. Participants with renal insufficiency were not excluded.15	**All-cause death and cardiovascular events**:<br>1) all-cause death: among the CKD subgroup, simvastatin didn't significantly decrease all-cause mortality;<br>2) cardiovascular events: decreased major coronary events, CHD mortality, and coronary revascularization. These results must be interpreted with caution given the small number of individuals (n=508) in this subgroup.<br><br>**Adverse events**: the side-effect profile was similar between the 2 treatment groups.
Nanayakkara 2007:<br>ATIC 2007	With chronic renal failure who had no manifest arterial occlusive disease.	**Lipid-lowering:** LDL-c was decreased significantly while the reduction of TG was not obvious.<br><br>**Renal function**: after 2 years of treatment in their patient population, they could not demonstrate a statistically significant effect on the eGFR between the groups. However, they were able to demonstrate a significant attenuation of the increase in urinary albumin excretion over time in the treatment group.

Cueto-Manzano2013:	1) 18-70 years old and had at least 1 month on CAPD.<br>2) exclusion criteria: inflammatory cause of ESRD, hepatic or malignant disease, any infectious disease within the last 3 months, drug intake with anti-inflammatory effect (including statins, steroids, or NSAIDs), or uncontrolled cholesterol levels≥400 mg/dL.	**Lipid-lowering:** TC and LDL-c significantly decreased in the pravastatin group. And median CRP was only significantly decreased in the pravastatin group.
Saltissi2002	1) had a fasting non-HDL c >135 mg/dL, with an LDL-c >116 mg/dL, and a TG concentration< 600 mg/dL.<br>2) exclusion criteria: impaired hepatic function, elevated creatine phosphokinase measurements, myocardial insufficiency, active infection or malignancy, and treatment with other lipid-lowering therapies.	**Lipid-lowering**: reduction in LDL-c was 24%. Simvastatin appears to be highly effective in reducing non-HDL–c concentrations in dialysis patients.<br>**Adverse events**: except for a higher incidence of adverse events for the body as a whole for the CAPD patients' placebo group, there were no differences between the groups.
Colin Baigent 2005<br>UK-HARP-I 2005	1) no definite indication for cholesterol-lowering therapy or aspirin.<br>2) exclusion criteria: if there was evidence of a recent history of acute uremia, history of chronic liver disease, inflammatory muscle disease, or creatine kinase [CK] level greater than 3 times the upper limit of normal (ULN), previous adverse reaction to a statin or history of aspirin hypersensitivity, concurrent treatment with a contraindicated drug, high immediate risk for bleeding, child-bearing potential in the absence of a reliable method of contraception, a life-threatening condition other than CKD or vascular disease, frequent nonattendance at clinics or known noncompliance with drug treatments, or alcohol or substance abuse.	**Lipid-lowering**: during a 1-year treatment period, simvastatin, 20 mg/d, produced a significant sustained reduction of LDL-c, TC, and TG, while HDL-c improvement was not obvious.<br>**Adverse events**: no drug-related adverse events difference was observed between the two groups.
Summary: LS is associated with a significant improvement of lipid profile, with an obvious reduction of LDL-c while the relatively slighter decrease of TG and increase of HDL-c. For cardiovascular risk reduction, moderate CKD patients can also benefit from LS treatment without serious side effects and the mechanisms are still controversial as this benefit seems to be independent of lipid-lowering effects. For the advanced CKD and dialysis groups. But some of them indicated that for those individuals with severe hyperlipidemia, lipid-lowering medication is associated with the reduction of cardiovascular risk. And for all CKD stratifications, no drug-related		

adverse events and significant negative influence on renal function were observed. In contrast, statin therapy appeared to exert a positive effect on the improvement of renal function and CKD progression. Therefore, to conclude, for moderate CKD patients, statin treatment can be initiated regardless of lipid concentration. But for dialysis patients without obvious hyperlipidemia, given the potential side effects, statin treatment is not recommended. And the cut-off lipid concentration for the initiation of lipid-lowering treatment among these end-stage CKD patients is not well defined and waiting for the study of more trials in the future.		
**Other medications**		
Lok2012<br>(FISH study)	1) patients with end-stage renal disease who required new arteriovenous graft access,<br>2) major exclusion criteria were reversible renal failure; active malignancy; pregnancy; malignant hypertension; active major bleed in the prior month; receiving more than 2 antiplatelet agents or anticoagulants (ie, concomitant use of aspirin and warfarin was not an exclusion); life expectancy less than 6 months; surgical revision of previous access, such as a jump graft (ie, the arteriovenous graft understudy needed to be a new graft); arteriovenous graft that failed before and including postoperative day 7; ingestion of any form of fish oil at the time of randomization; allergy to fish or fish products; and enrollment in another interventional study of arteriovenous grafts.	**All-cause death and cardiovascular events:**<br>1) all-cause death: NA<br>**2)** cardiovascular events: fish oil improved cardiovascular event-free survival and rate as well as improved blood pressure and led to a reduction in the use of anti-hypertensive medications.
Bowden2009	1) ESRD patients receive chronic hemodialysis treatment 3 times per week.<br>2) exclusion criteria for this study comprised a life expectancy of fewer than 6 months (based on physician prognosis), pregnancy, a history of hemodialysis noncompliance, previous medication noncompliance, no desire to participate in the study, or age below 18 years.	**Lipid-lowering**: omega-3 fatty acid could effectively reduce LDL particle number.

Daud2012	1) adults who were undergoing routine hemodialysis.<br>2) patients who were residents of nursing homes, receiving intradialytic parenteral nutrition or tube feeding, undergoing 8 hours of dialysis treatment, undergoing nocturnal dialysis, and serum albumin. 3.9 g/dL, or who had fish allergies were excluded.	**Lipid-lowering**: although compared to the controlled group, omega-3 failed to lower LDL-c and improve HDL-c levels significantly, it effectively reduced the LDL-HDL ratio in hemodialysis patients.
Wanner2014	1) dialysis patients≥ 18 years.<br>2) patients were excluded if they had serum albumin < 3.0 g/dL, serum triglycerides > 600 mg/dL, serum LDL-c > 190 mg/dL, body mass index ≤ 16.0 kg/m2, or ≥ 40.0 kg/ m2, significant gastrointestinal abnormalities or liver dysfunction, a history of rhabdomyolysis or myopathy, or used medications contraindicated for coadministration with simvastatin.	**Lipid-lowering**: colestilan was non-inferior to simvastatin at reducing LDL-c, and was more effective than a placebo at maintaining control of LDLC levels.<br>**Adverse events**: adverse events were generally of mild or moderate severity. No serious AEs occurred and were considered related to the study medication in the colestilan group. Gastrointestinal adverse events were the most common adverse event.
Locatelli2013	1) dialysis patients with age≥ 18 years; have stable phosphate	**Lipid-lowering:** compared to placebo, colestilan could significantly reduce LDL-c and TC.

	control using phosphate-binding medication for at least 1 month before screening; serum phosphorus level <2.42 mmol/L. The calcium dialysate content had to be between 2 and 3.5 mEq/L and remain constant throughout the study. Furthermore, to qualify for randomization serum phosphorus had to be ≥1.94 mmol/L (6.0 mg/dL) and at least 15% greater than at screening, after both 2 and 3 weeks of phosphate binder washout, or ≥2.58 mmol/L (8.0 mg/dL) and at least 15% greater than at screening after 1 week of washout.<br>2) exclusion criteria: clinically significant medical comorbidities which could substantially compromise patient safety or interfere with study procedures; serum albumin level <30.0 g/L, intact parathyroid hormone (iPTH) levels consistently/frequently >1000 pg/mL, BMI ≤16.0 or ≥40.0 kg/m2, a history of significant gastrointestinal abnormalities including motility problems or major gastrointestinal surgery, biliary obstruction or proven liver dysfunction or liver function tests three times the upper limit of normal for at least two of alanine aminotransferase, aspartate aminotransferase, and gamma-glutamyl transferase.	**Adverse events**: there was no notable difference in the incidence of treatment-emergent AEs reported with colestilan and placebo.
Kalantar-Zadeh2021<br>(BETonthe composite cardiovascular events study)	1) aged over 18 years, with type 2 diabetes, low HDL cholesterol levels (,40 mg/dl in men or 45 mg/dl in women), and recent acute coronary syndrome.<br>2) exclusion criteria included an eGFR,30 ml/min per 1.73 m2, liver transaminase levels .1.5 times the upper limit of normal, and total bilirubin level greater than the upper limit of normal.	**All-cause death and cardiovascular events:**<br>**1)** all-cause death**:** NA<br>**2)** cardiovascular events: apabetalone may reduce the incidence of major adverse cardiovascular events in patients with CKD and type 2 diabetes who have a high burden of cardiovascular disease.<br>**Lipid-lowering**: apabetalone didn't reduce LDL-c but improved HDL-c significantly.<br>**Adverse events**: no drug-related adverse events difference was observed between the two groups.

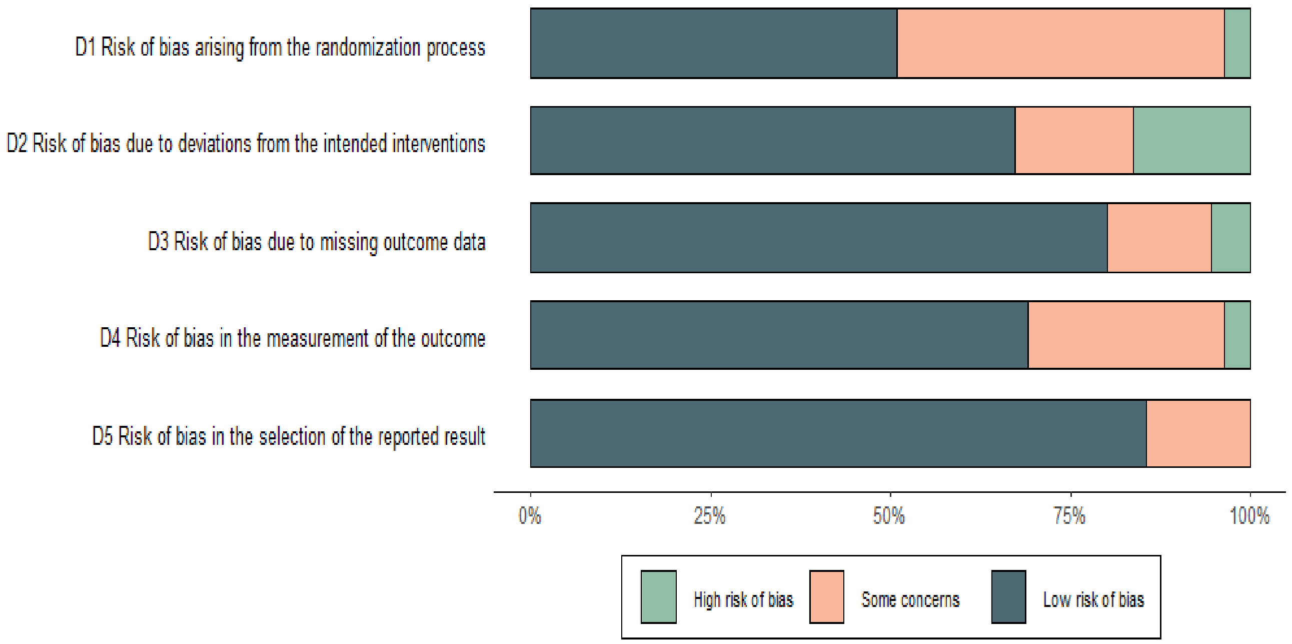

Bar chart to show risk of bias in included studies.

S5. Risk of bias in individual trials (Rob2)

Risk of bias summary.

Study	D1	D2	D3	D4	D5	Overall
4S 2009	Some concerns	Low	Low	Low	Low	Some concerns
AFCAPS/TexCAPS 2010	Some concerns	Some concerns	Low	Low	Low	Some concerns
4D 2005	Low	Low	Low	Low	Low	Low
ALLHAT 2008	High	High	High	Some concerns	Low	High
ALLIANCE 2004	Some concerns	Some concerns	Some concerns	Low	Low	Some concerns
ASUCA 2016	Some concerns	Some concerns	High	Some concerns	Low	High
ATIC 2007	Low	Low	Some concerns	Some concerns	Low	Some concerns
AURORA 2009	Some concerns	Low	Low	Low	Low	Some concerns
Bianchi 2003	Some concerns	Low	Low	Low	Some concerns	Some concerns

Burmeister 2009	Some concerns	Low	Low	Low	Low	Some concerns
CARDS 2009	Some concerns	Low	Low	Low	Low	Some concerns
CARE 1996	Some concerns	Low	Low	Low	Low	Some concerns
COMBO I	Some concerns	Low	Low	Low	Low	Some concerns
COMBO II	Some concerns	Low	Low	Low	Low	Some concerns
Dogra 2007	Low	Some concerns	Low	Low	Some concerns	Some concerns
Fassett 2010	Low	Low	Low	Low	Low	Low
FH I	Low	Low	Low	Low	Low	Low
FH II	Low	Low	Low	Low	Low	Low
FOURIER 2017	Low	Low	Low	Low	Low	Low
Goicoechea 2006	Some concerns	High	Some concerns	Some concerns	Some concerns	High
Harris 2002	Some concerns	High	Low	Some concerns	Low	High
HIGH FH	Low	Low	Low	Low	Low	Low
Holmberg 2005	Some concerns	Some concerns	High	Some concerns	Some concerns	High
HIJ-PROPER 2017	Some concerns	Some concerns	Low	Some concerns	Low	Some concerns
IDEAL 2005	Low	Some concerns	Low	Low	Low	Some concerns
IMPROVE-IT 2015	Some concerns	Low	Low	Low	Low	Some concerns
JUPITER 2008	low	Low	Low	Low	Low	low
Landray 2006	Some concerns	High	Some concerns	Low	Some concerns	High
LIPID 1996	Some concerns	Low	Low	Low	Low	Some concerns
LIPS 2002	Some concerns	Low	Low	Low	Low	Some concerns
LONG TERM	Low	Low	Low	Low	Low	Low
MEGA 2006	Low	Some concerns	Some concerns	Low	Low	Some concerns
ODYSSEYOUTCOMES 2018	Low	Low	Low	Low	Low	Low

OPTIONS I	Low	Low	Low	Some concerns	Low	Some concerns
OPTIONS II	Low	Low	Low	Some concerns	Low	Some concerns
Saltissi 2002	Some concerns	Low	Low	Some concerns	Low	Some concerns
Samuelsson 1997	High	High	Low	Some concerns	Some concerns	High
SHARP 2011	Low	Low	Low	Low	Low	Low
SAGE 2007	Low	Low	Low	Low	Low	Low
STEGMAYR 2005	Some concerns	High	Some concerns	Some concerns	Low	High
Suzuki 2014	Some concerns	High	Low	high	Low	High
TING 2012	Low	Low	Low	Some concerns	Low	Some concerns
TNT 2007	Low	Low	Low	Some concerns	Low	Some concerns
UK-HARP-I 2005	Low	High	Low	Low	Low	High
VA-HIT 1999	Low	Low	Low	Low	Low	Low
Verma 2005	Some concerns	Low	Low	Some concerns	Some concerns	Some concerns
Weinstein 2013	Low	Low	Some concerns	Low	Low	Some concerns
WOSCOPS 1995	Some concerns	Low	Low	Low	Low	Some concerns
Yasuda 2004	Some concerns	High	Some concerns	High	Some concerns	High
Kalantar-Zadeh2021	Low	Low	Low	Low	Low	Low
Lok2012	Low	Low	Low	Low	Low	Low
Daud2012	Low	Low	Low	Low	Low	Low
Bowden2009	Low	Low	Low	Low	Low	Low
Wanner2014	Low	Low	Low	Low	Low	Low
Locatelli2014	Low	Some concerns	Low	Low	Low	Some concerns

Footnotes: D1: Risk of bias arising from the randomization process; D2: Risk of bias due to deviations from the intended interventions; D3: Risk of bias due to missing outcome data; D4: Risk of bias in the measurement of the outcome; D5: Risk of bias in the selection of the reported result; Overall: Overall risk of bias

S6. Supplemental information for all the outcomes of interest

S6.1. All-cause death

S6.1.1. The funnel plot

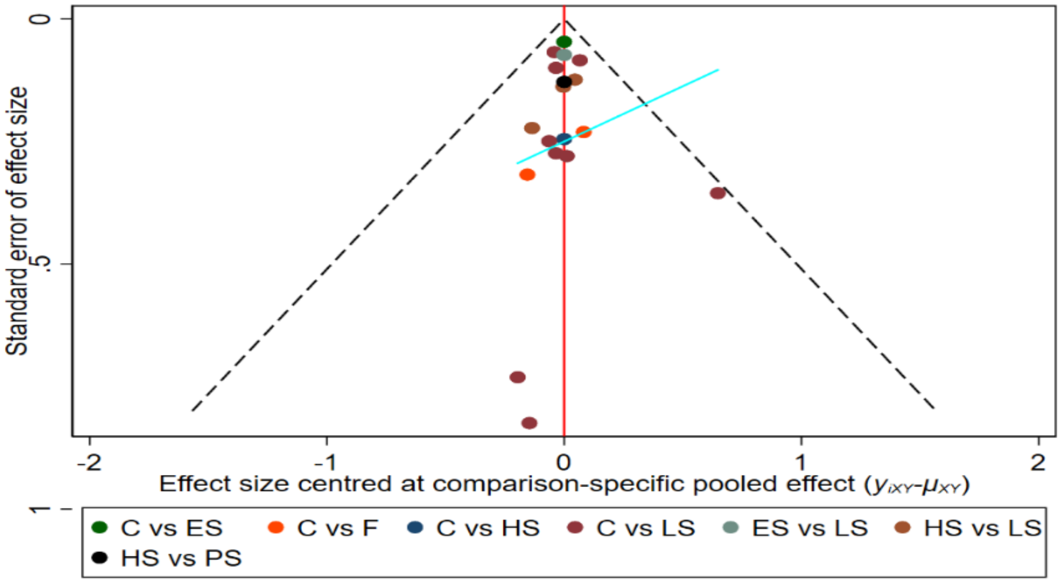

S6.1.2. The analysis of consistency and heterogeneity

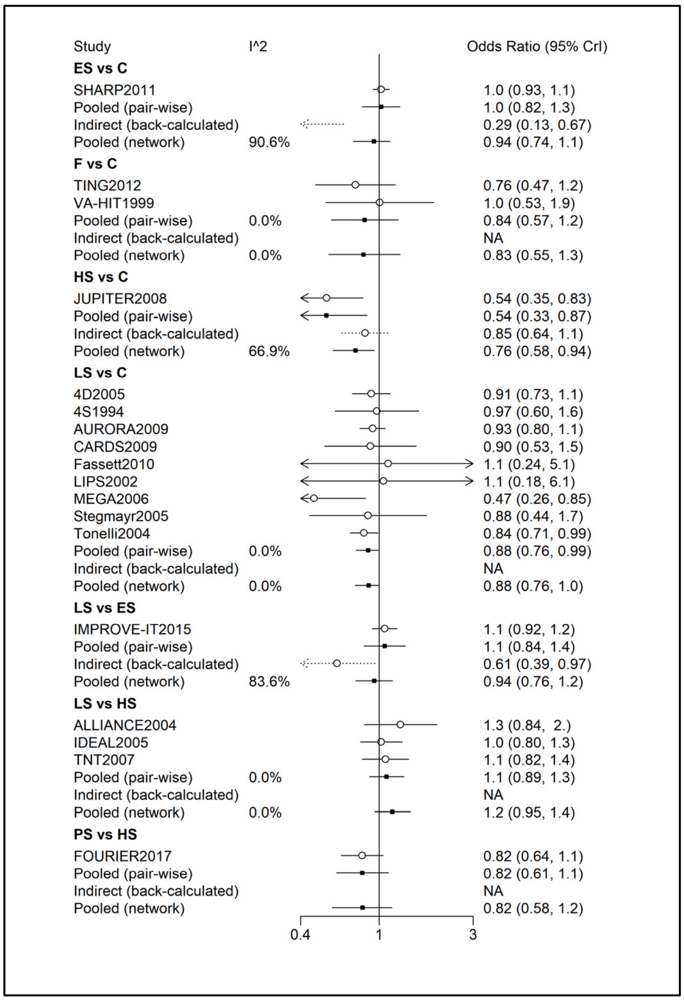

A

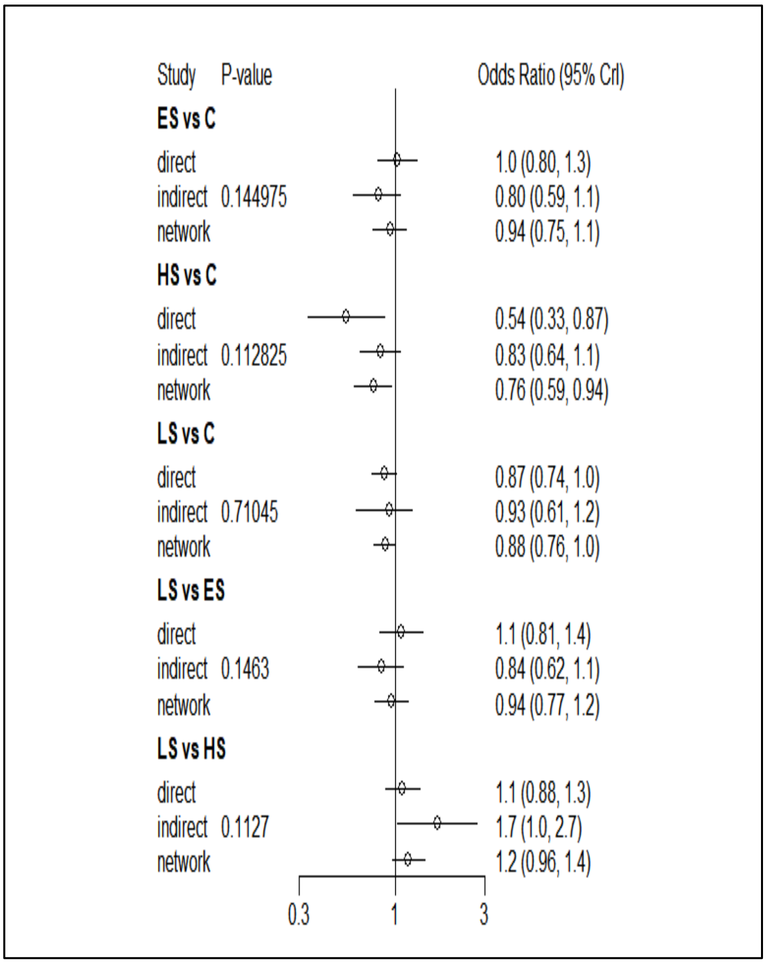

B

The analyses of heterogeneity (A) and consistency (B).

S6.1.3. Trace plots, density plots and Brooks-Gelman-Rubin diagnosis plots.

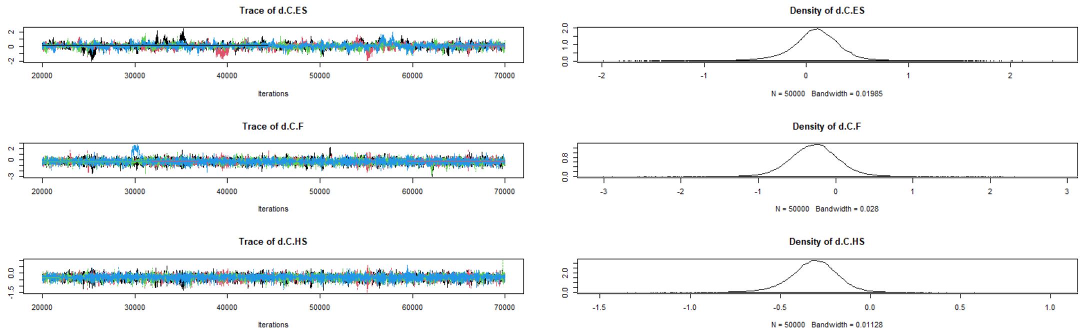

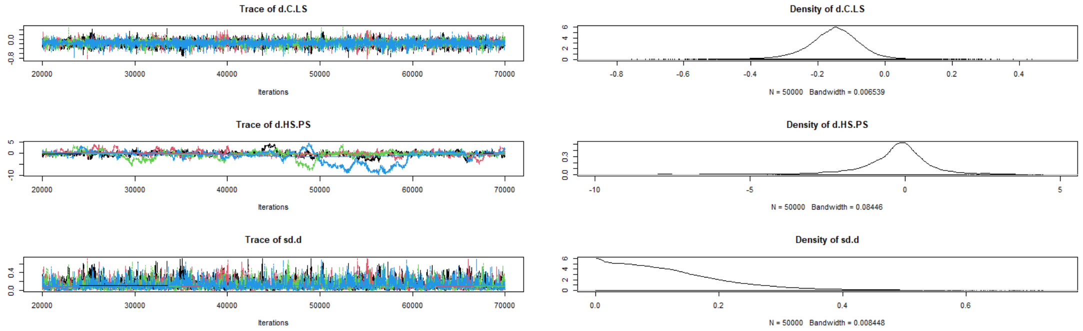

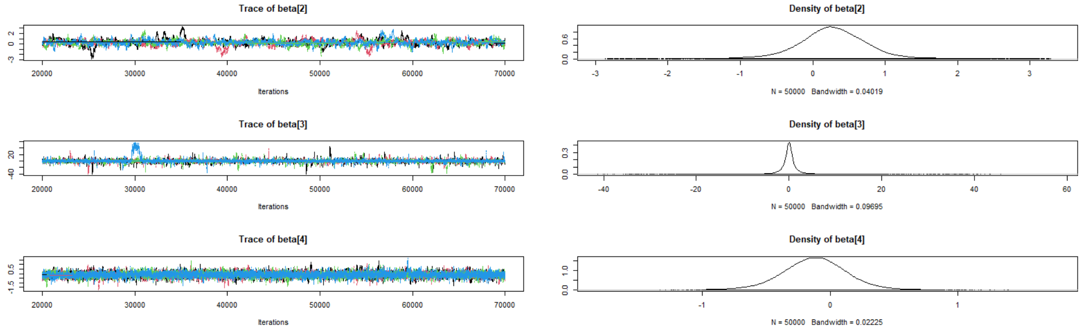

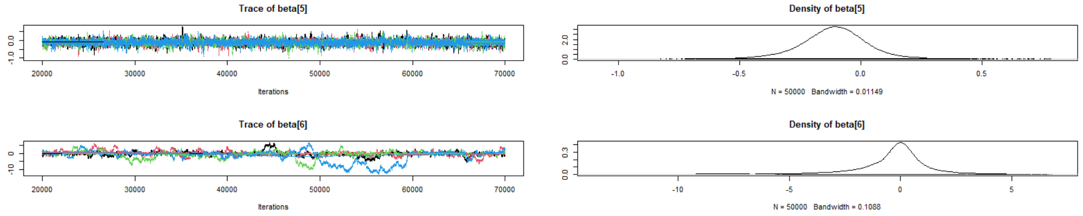

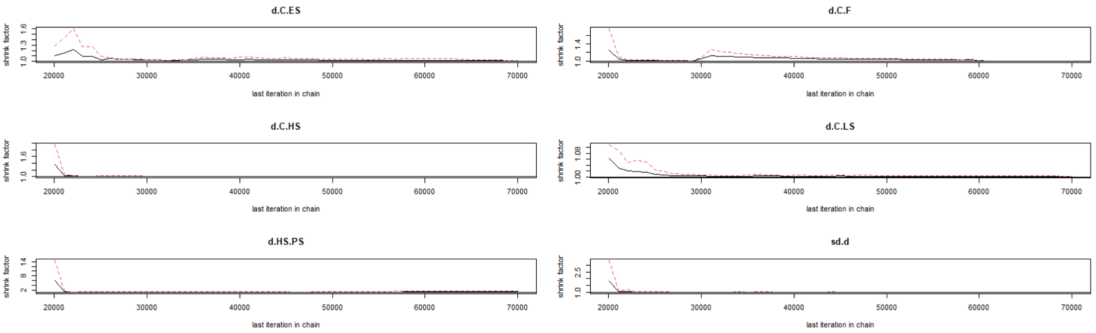

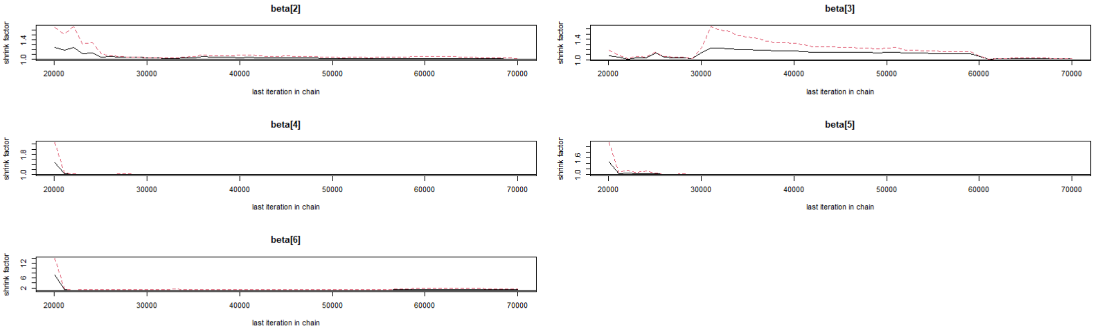

Potential scale reduction factors:

Point est. Upper C.I.		
d.C.ES	1.01	1.02
d.C.F	1.00	1.00
d.C.HS	1.00	1.00
d.C.LS	1.00	1.00
d.HS.PS	1.22	1.62
sd.d	1.00	1.00
beta[2]	1.01	1.02
beta[3]	1.02	1.03
beta[4]	1.00	1.00
beta[5]	1.00	1.00
beta[6]	1.22	1.65
Multivariate psrf: 1.09		

S6.1.4. The rank probabilities of each treatment.

I \ R	1	2	3	4	5	6
C	0.000125	0.000250	0.003500	0.051875	0.328500	0.615750
ES	0.007000	0.028875	0.109500	0.269375	0.397000	0.188250
F	0.123375	0.197000	0.237625	0.147750	0.117000	0.177250
HS	0.081625	0.601250	0.259250	0.041625	0.013625	0.002625
LS	0.003375	0.031375	0.347125	0.476000	0.134125	0.008000
PS	0.784500	0.141250	0.043000	0.013375	0.009750	0.008125

I: interventions; R: rank. ☐ **Rank the highest**

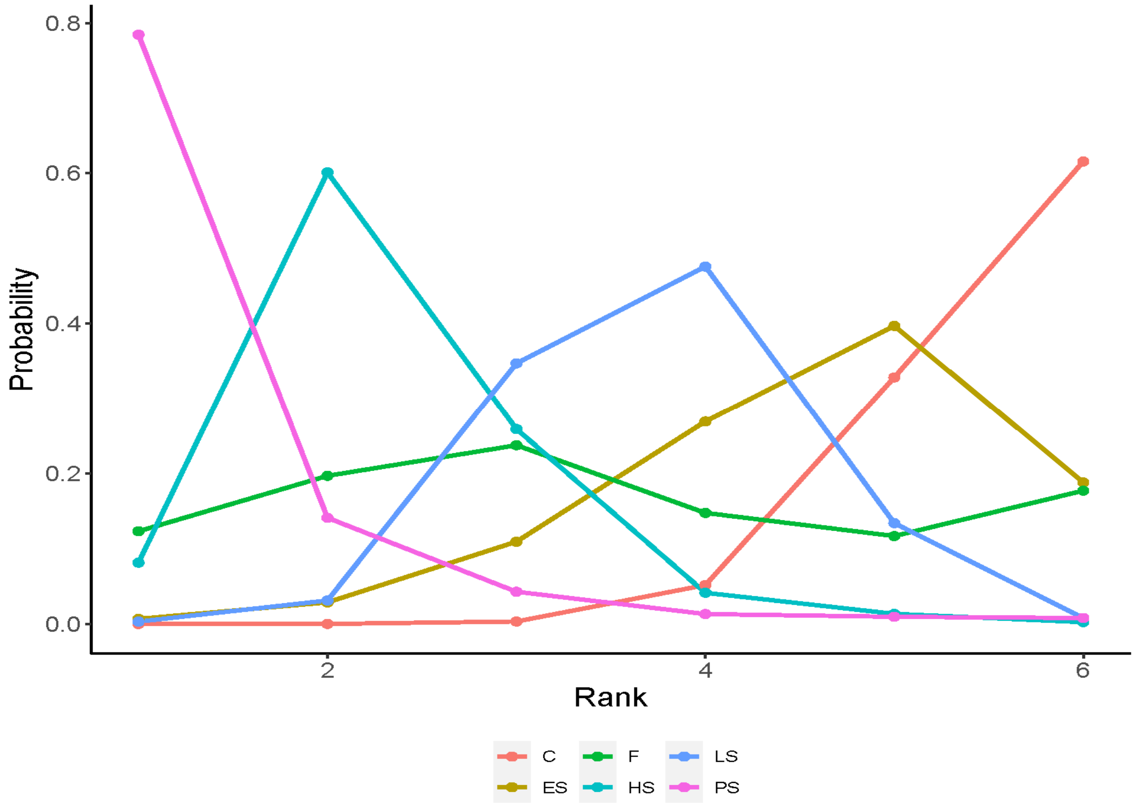

S6.1.5. The regression analysis for LDL-c.

Quantiles for each variable:

	2.5%	25%	50%	75%	97.5%
d.C.ES	-0.41	-0.03	0.10	0.23	0.55
d.C.F	-1.01	-0.48	-0.28	-0.08	0.41
d.C.HS	-0.59	-0.39	-0.30	-0.22	-0.07
d.C.LS	-0.33	-0.19	-0.15	-0.10	-0.003
d.HS.PS	-3.66	-0.63	-0.13	0.37	2.42
sd.d	0.004	0.04	0.09	0.16	0.35
beta[2]	-0.60	0.01	0.27	0.54	1.17
beta[3]	-8.98	-0.72	-0.00	0.71	7.71
beta[4]	-0.61	-0.27	-0.11	0.05	0.38
beta[5]	-0.40	-0.19	-0.11	-0.03	0.16
beta[6]	-5.07	-0.64	-0.01	0.62	3.56

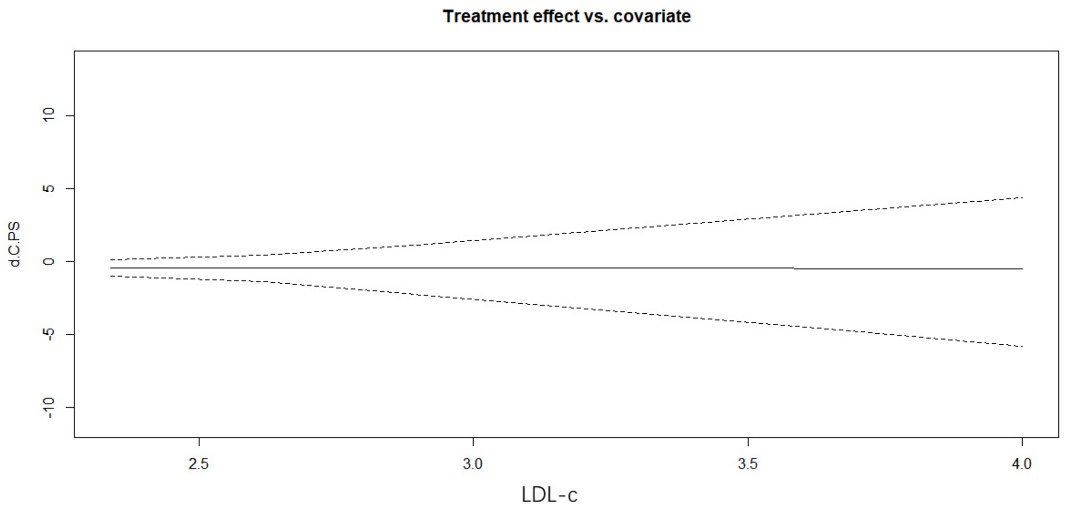

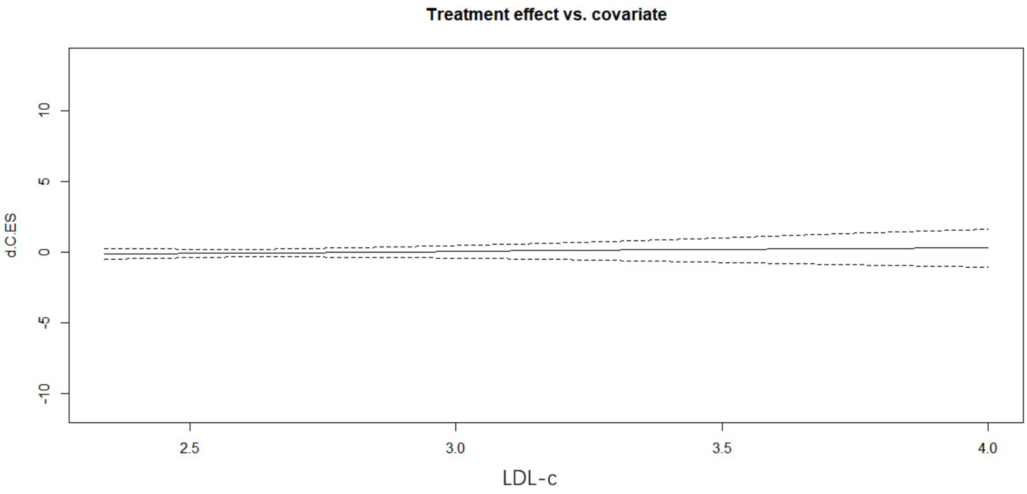
Treatment effect vs. covariate
d.C.ES
10
5
0
-5
-10
2.5
3.0
3.5
4.0
LDL-c

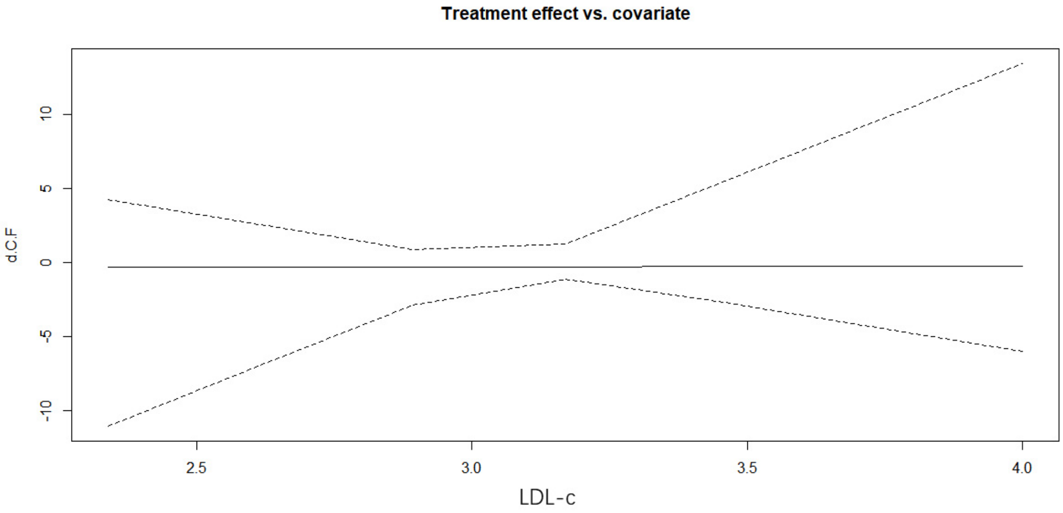
Treatment effect vs. covariate
d.C.F
10
5
0
-5
-10
2.5
3.0
3.5
4.0
LDL-c

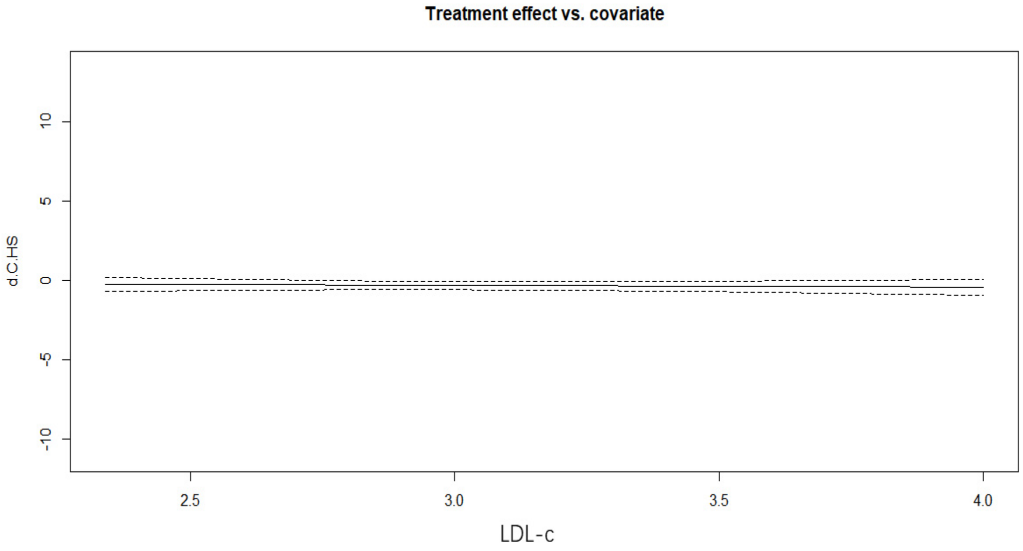
Treatment effect vs. covariate
d.C.HS
10
5
0
-5
-10
2.5
3.0
3.5
4.0
LDL-c

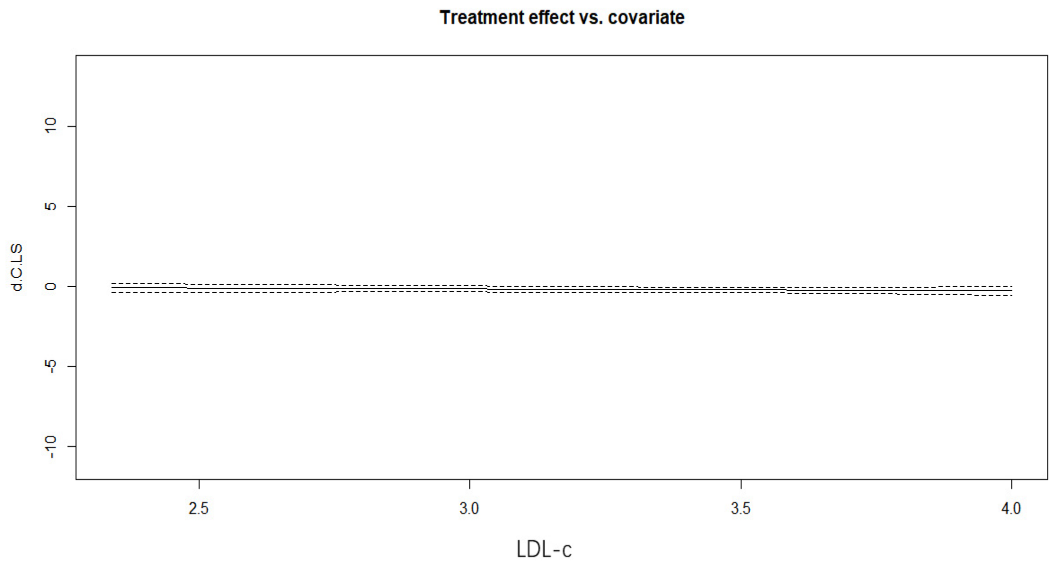
Treatment effect vs. covariate
d.C.LS
10
5
0
-5
-10
2.5
3.0
3.5
4.0
LDL-c

S6.1.6. Network meta-analysis was performed with the frequentist model

1). SUCRA-based ranking of all treatments in CKD patients.

C	1.061 (0.973, 1.156)	1.193 (0.819, 1.736)	1.270 (1.070, 1.508)	1.108 (1.021, 1.202)	1.544 (1.142, 2.087)
0.943 (0.865, 1.028)	ES	1.124 (0.765, 1.653)	1.198 (0.998, 1.437)	1.044 (0.945, 1.155)	1.456 (1.070, 1.981)
0.839 (0.576, 1.221)	0.889 (0.605, 1.308)	F	1.065 (0.705, 1.610)	0.929 (0.632, 1.364)	1.295 (0.800, 2.096)
0.787 (0.663, 0.934)	0.835 (0.696, 1.002)	0.939 (0.621, 1.419)	HS	0.872 (0.746, 1.020)	1.216 (0.949, 1.558)
0.903 (0.832, 0.980)	0.958 (0.866, 1.059)	1.077 (0.733, 1.582)	1.147 (0.981, 1.341)	LS	1.394 (1.040, 1.869)
0.648 (0.479, 0.875)	0.687 (0.505, 0.934)	0.772 (0.477, 1.250)	0.823 (0.642, 1.054)	0.717 (0.535, 0.962)	PS

2). NMA after excluding the trials with a high risk of bias

C	1.055 (0.971, 1.146)	1.193 (0.820, 1.736)	1.234 (1.030, 1.478)	1.093 (1.008, 1.186)	1.500 (1.105, 2.035)
0.948 (0.873, 1.030)	ES	1.131 (0.770, 1.660)	1.170 (0.967, 1.415)	1.037 (0.940, 1.144)	1.422 (1.042, 1.941)
0.838 (0.576, 1.220)	0.884 (0.602, 1.299)	F	1.034 (0.682, 1.569)	0.917 (0.624, 1.346)	1.258 (0.775, 2.040)
0.811 (0.677, 0.971)	0.855 (0.707, 1.034)	0.967 (0.637, 1.466)	HS	0.886 (0.750, 1.047)	1.216 (0.950, 1.555)
0.915 (0.843, 0.993)	0.965 (0.874, 1.064)	1.091 (0.743, 1.601)	1.129 (0.955, 1.334)	LS	1.372 (1.019, 1.847)
0.667 (0.491, 0.905)	0.703 (0.515, 0.960)	0.795 (0.490, 1.290)	0.823 (0.643, 1.052)	0.729 (0.541, 0.981)	PS

S6.1.7. Sensitivity analysis by by excluding studies enrolling a large number of patients with cardiovascular disease

Study or Subgroup	Experimental Events	Experimental Total	Control Events	Control Total	Weight	Odds Ratio M-H, Fixed, 95% CI
**1.1.1 ES vs C**						
SHARP2011	1142	4650	1115	4620	44.0%	1.02 [0.93, 1.13]
**Subtotal (95% CI)**		**4650**		**4620**	**44.0%**	**1.02 [0.93, 1.13]**
Total events	1142		1115			
Heterogeneity: Not applicable						
Test for overall effect: Z = 0.48 (P = 0.63)						
**1.1.2 F vs C**						
TING2012	43	295	41	223	2.1%	0.76 [0.47, 1.21]
**Subtotal (95% CI)**		**295**		**223**	**2.1%**	**0.76 [0.47, 1.21]**
Total events	43		41			
Heterogeneity: Not applicable						
Test for overall effect: Z = 1.16 (P = 0.25)						
**1.1.3 LS vs C**						
4D2005	297	619	320	636	8.6%	0.91 [0.73, 1.14]
AURORA2009	636	1389	660	1384	18.7%	0.93 [0.80, 1.08]
CARDS2009	27	482	30	488	1.5%	0.91 [0.53, 1.55]
Fassett2010	4	58	4	65	0.2%	1.13 [0.27, 4.74]
IMPROVE-IT2015	463	1870	491	1891	19.2%	0.94 [0.81, 1.09]
MEGA2006	16	1471	34	1507	1.7%	0.48 [0.26, 0.87]
Stegmayr2005	43	70	47	73	0.9%	0.88 [0.45, 1.74]
**Subtotal (95% CI)**		**5959**		**6044**	**50.8%**	**0.91 [0.83, 1.00]**
Total events	1486		1586			
Heterogeneity: Chi² = 4.80, df = 6 (P = 0.57); I² = 0%						
Test for overall effect: Z = 1.98 (P = 0.05)						
**1.1.4 HS vs C**						
JUPITER2008	34	1638	61	1629	3.1%	0.54 [0.36, 0.83]
**Subtotal (95% CI)**		**1638**		**1629**	**3.1%**	**0.54 [0.36, 0.83]**
Total events	34		61			
Heterogeneity: Not applicable						
Test for overall effect: Z = 2.80 (P = 0.005)						
**Total (95% CI)**		**12542**		**12516**	**100.0%**	**0.95 [0.89, 1.01]**
Total events	2705		2803			
Heterogeneity: Chi² = 15.34, df = 9 (P = 0.08); I² = 41%						
Test for overall effect: Z = 1.68 (P = 0.09)						
Test for subgroup differences: Chi² = 10.58, df = 3 (P = 0.01), I² = 71.6%						

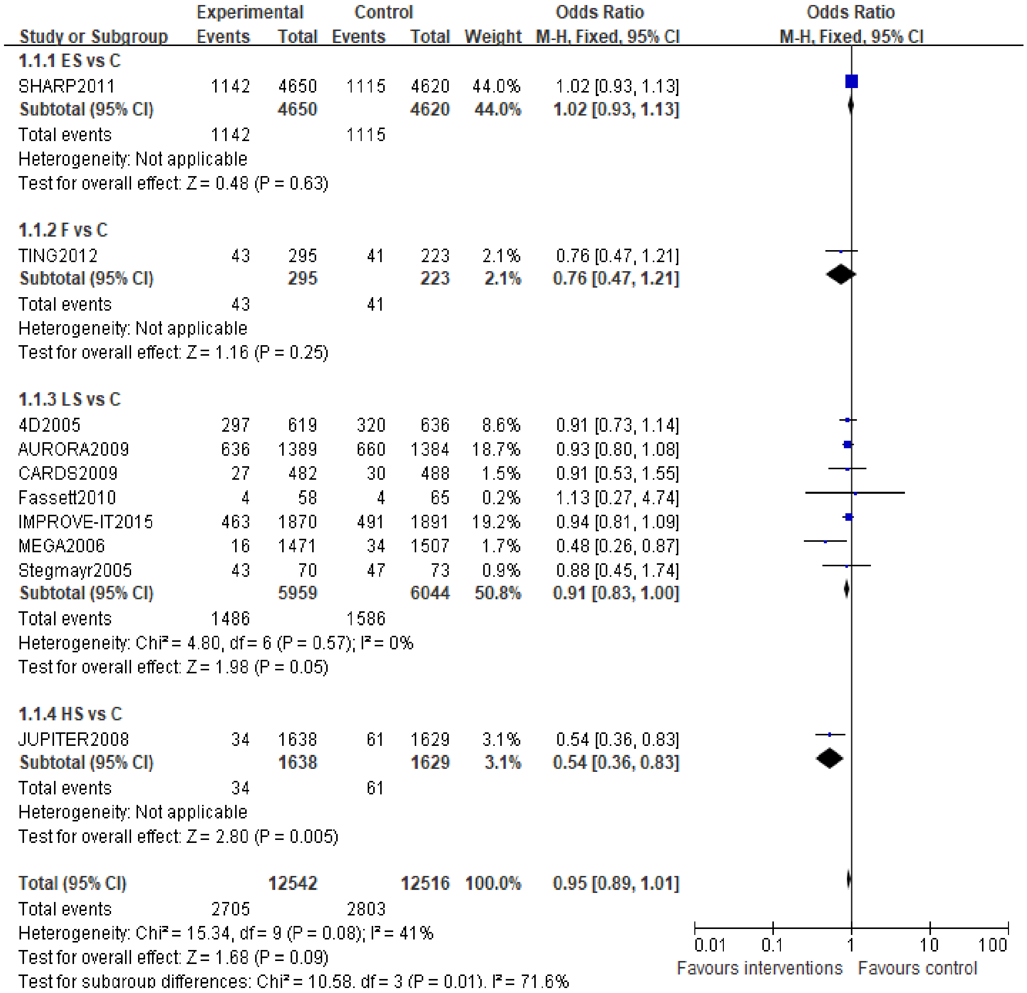

S6.2. The composite cardiovascular events

S6.2.1. the definitions of the composite cardiovascular events in trials included in the NMA

Study	Composite cardiovascular outcome
**PS**	
Charytan2019<br>(Post hoc analysis of FOURIER)	Cardiovascular death, myocardial infarction, stroke, hospitalization for unstable angina, or coronary revascularization.
Toth2018<br>(Pooled analysis of 8 trials)	Coronary heart disease death, nonfatal myocardial infarction, fatal and nonfatal ischemic stroke, unstable angina requiring hospitalization.
Jose2020<br>(post hoc of the ODYSSEY OUTCOMES)	Death due to coronary heart disease, nonfatal myocardial infarction, ischaemic stroke, or unstable angina requiring hospitalization.
**ES**	
Hagiwara2017<br>(subgroup analysis of HIJ-PROPER)	All-cause death, non-fatal myocardial infarction, non-fatal stroke, unstable angina, and ischemia-driven revascularization.
Baigent2011<br>(SHARP RCT)	Non-fatal myocardial infarction or coronary death, non-hemorrhagic stroke, or any arterial revascularisation procedure.
Stanifer2017<br>(post hoc analysis of IMPROVE-IT)	Cardiovascular death, major coronary event, or nonfatal stroke

**F**	
TONELLI2004<br>(post hoc of VA-HIT)	Fatal CHD, nonfatal myocardial infarction, and stroke
TING2012<br>(RCT)	Composite of cardiovascular death, myocardial infarction, stroke, and coronary/carotid revascularization.
**HS**	
Shepherd2007<br>(post hoc of TNT)	Death from CHD, nonfatal non-procedure-related myocardial infarction, resuscitation after cardiac arrest, or fatal or nonfatal stroke
Shepherd2007<br>(post hoc of TNT)	Cardiac death, nonfatal MI, resuscitated cardiac arrest, cardiac revascularization, and unstable angina requiring hospitalization
Holme2009<br>(post hoc of IDEAL)	the composite cardiovascular events
Ridker2010<br>(secondary analysis from JUPITER)	Nonfatal myocardial infarction, nonfatal stroke, hospital stay for unstable angina, arterial revascularization, or cardiovascular death.
**LS**	
Wanner2005<br>(RCT: 4D); Ma¨rz2005: (post hoc of 4D)	The primary endpoint was a composite of death from cardiac causes, fatal stroke, nonfatal myocardial infarction, or nonfatal stroke, whichever occurred first
Tonelli2004<br>(data from 3p: WOSCOPS, CARE, LIPID)<br>TONELLI2003: post hoc of CARE	Fatal/nonfatal CAD, CABG, PTCA, or stroke

Lemos2005<br><br>(substudy of LIPS)	Cardiac death, nonfatal myocardial infarction, and reinterventions not related to restenosis.
STEGMAYR/HOLMBERG2005 (RCT)	All-cause mortality, non-lethal acute myocardial infarction, coronary artery bypass graft surgery, and percutaneous transluminal coronary angioplasty.
Chonchol2006<br><br>(post hoc analysis of 4S)	Coronary deaths, MI, resuscitated cardiac arrest
Nakamura2009<br><br>(Post hoc of MEGA)	Major adverse cardiovascular events were a composite of cardiovascular death, myocardial infarction, or stroke.
Fellström2009<br><br>(RCT: AURORA)	Death from cardiovascular causes, MI, stroke
Colhoun2009<br><br>(post hoc of CARDS)	Acute coronary heart disease events, coronary revascularization, or stroke

S6.2.2. The pairwise meta-analysis for the composite cardiovascular events

Study or Subgroup	Experimental Events	Experimental Total	Control Events	Control Total	Weight	Odds Ratio M-H, Random, 95% CI
**1.2.1 PCSK9i vs. HS**						
FOURIER 2017	296	2302	303	2141	80.2%	0.90 [0.75, 1.06]
Toth 2018	8	315	8	152	19.8%	0.47 [0.17, 1.27]
**Subtotal (95% CI)**		**2617**		**2293**	**100.0%**	**0.79 [0.48, 1.30]**
Total events	304		311			
Heterogeneity: Tau² = 0.07; Chi² = 1.56, df = 1 (P = 0.21); I² = 36%						
Test for overall effect: Z = 0.93 (P = 0.35)						
**1.2.2 HS VS. C**						
JUPITER 2008	40	1638	71	1629	100.0%	0.55 [0.37, 0.81]
**Subtotal (95% CI)**		**1638**		**1629**	**100.0%**	**0.55 [0.37, 0.81]**
Total events	40		71			
Heterogeneity: Not applicable						
Test for overall effect: Z = 2.98 (P = 0.003)						
**1.2.3 HS vs.LS**						
ALLIANCE 2004	78	286	105	293	25.6%	0.67 [0.47, 0.96]
IDEAL 2005	187	1162	196	1159	37.4%	0.94 [0.76, 1.17]
TNT 2007	149	1602	202	1505	36.9%	0.66 [0.53, 0.83]
**Subtotal (95% CI)**		**3050**		**2957**	**100.0%**	**0.76 [0.59, 0.97]**
Total events	414		503			
Heterogeneity: Tau² = 0.03; Chi² = 5.60, df = 2 (P = 0.06); I² = 64%						
Test for overall effect: Z = 2.17 (P = 0.03)						
**1.2.4 LS+Eze. vs. LS**						
HIJ-PROPER 2017	73	206	92	218	10.3%	0.75 [0.51, 1.11]
IMPROVE-IT 2015	655	1870	718	1891	89.7%	0.88 [0.77, 1.01]
**Subtotal (95% CI)**		**2076**		**2109**	**100.0%**	**0.87 [0.76, 0.98]**
Total events	728		810			
Heterogeneity: Tau² = 0.00; Chi² = 0.56, df = 1 (P = 0.45); I² = 0%						
Test for overall effect: Z = 2.23 (P = 0.03)						
**1.2.5 Eze.+LS vs. C**						
SHARP 2011	526	4650	619	4620	100.0%	0.82 [0.73, 0.93]
**Subtotal (95% CI)**		**4650**		**4620**	**100.0%**	**0.82 [0.73, 0.93]**
Total events	526		619			
Heterogeneity: Not applicable						
Test for overall effect: Z = 3.05 (P = 0.002)						
**1.2.6 F vs. C**						
TING 2012	57	295	60	224	37.1%	0.65 [0.43, 0.99]
TING 2012	57	295	60	224	37.1%	0.65 [0.43, 0.99]
VA-HIT 1999	32	199	49	200	25.7%	0.59 [0.36, 0.97]
**Subtotal (95% CI)**		**789**		**648**	**100.0%**	**0.64 [0.50, 0.82]**
Total events	146		169			
Heterogeneity: Tau² = 0.00; Chi² = 0.12, df = 2 (P = 0.94); I² = 0%						
Test for overall effect: Z = 3.50 (P = 0.0005)						
**1.2.7 LS vs. C**						
4D 2005	226	619	243	636	17.3%	0.93 [0.74, 1.17]
4S 1994	53	245	77	260	11.3%	0.66 [0.44, 0.98]
AURORA 2009	396	1389	408	1384	19.7%	0.95 [0.81, 1.12]
CARDS 2009	25	482	42	488	8.6%	0.58 [0.35, 0.97]
LIPS 2002	23	150	47	160	7.7%	0.44 [0.25, 0.76]
MEGA 2006	25	1471	60	1507	9.5%	0.42 [0.26, 0.67]
STEGMAYR 2005	49	70	57	73	5.0%	0.65 [0.31, 1.39]
Toneli 2004	573	2217	730	2274	20.9%	0.74 [0.65, 0.84]
**Subtotal (95% CI)**		**6643**		**6782**	**100.0%**	**0.71 [0.58, 0.85]**
Total events	1370		1664			
Heterogeneity: Tau² = 0.04; Chi² = 21.90, df = 7 (P = 0.003); I² = 68%						
Test for overall effect: Z = 3.59 (P = 0.0003)						

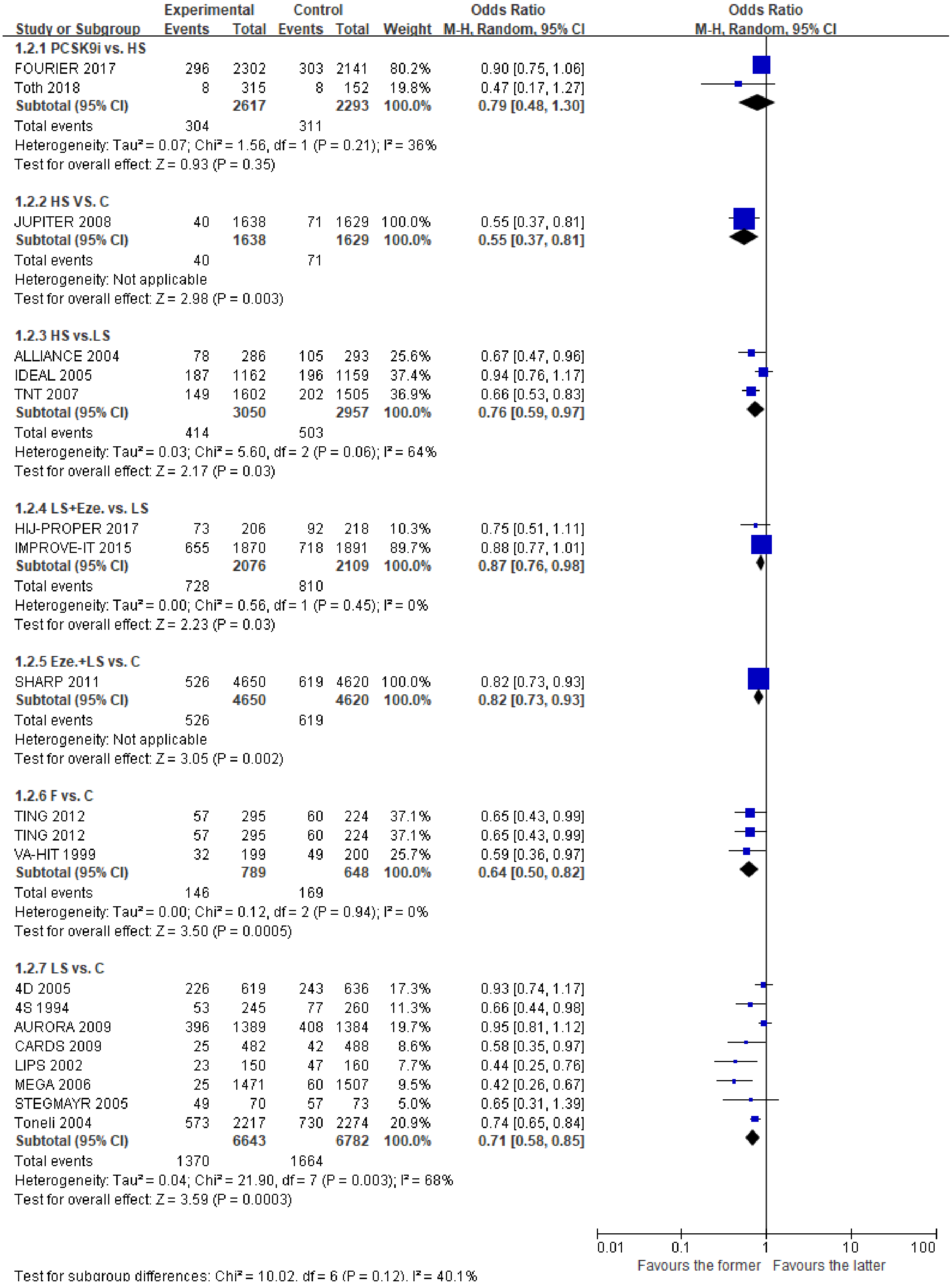

Test for subgroup differences: Chi² = 10.02, df = 6 (P = 0.12), I² = 40.1%

S6.2.3. The funnel plot

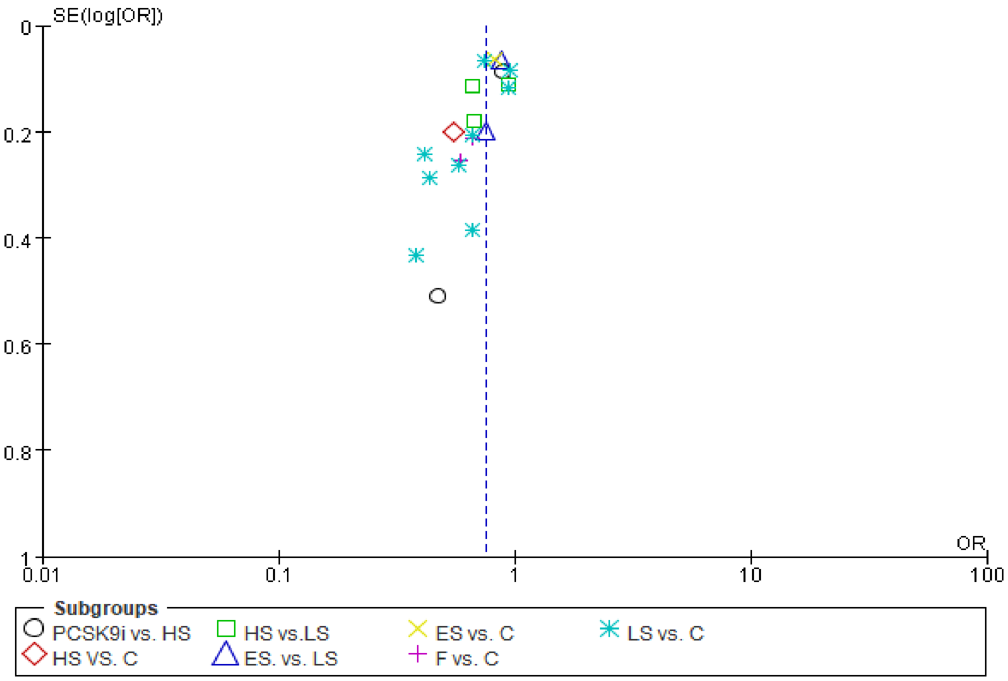

S6.2.4. The analysis of consistency (A) and heterogeneity (B).

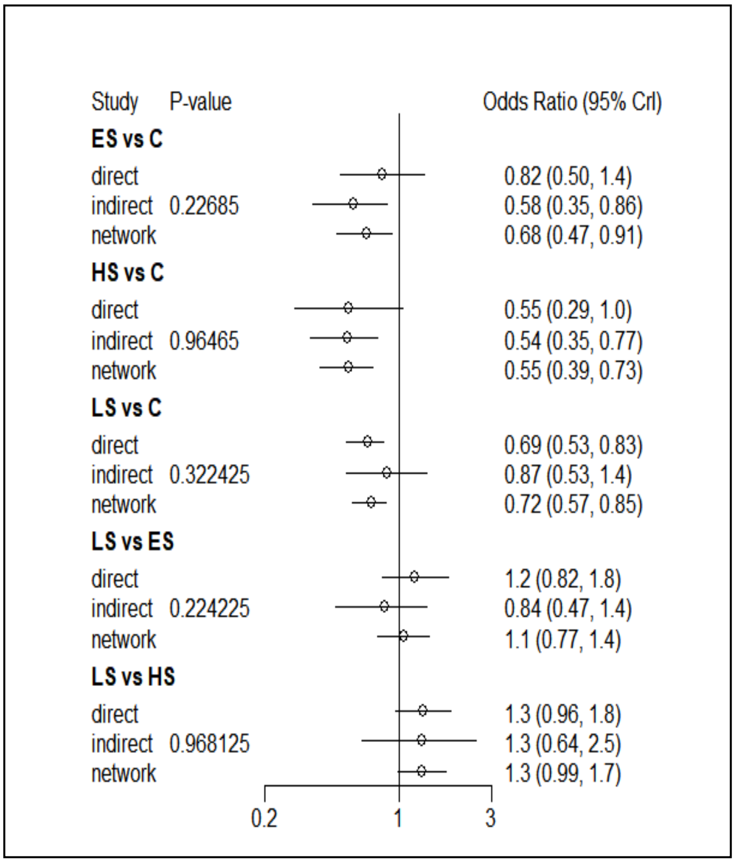

A

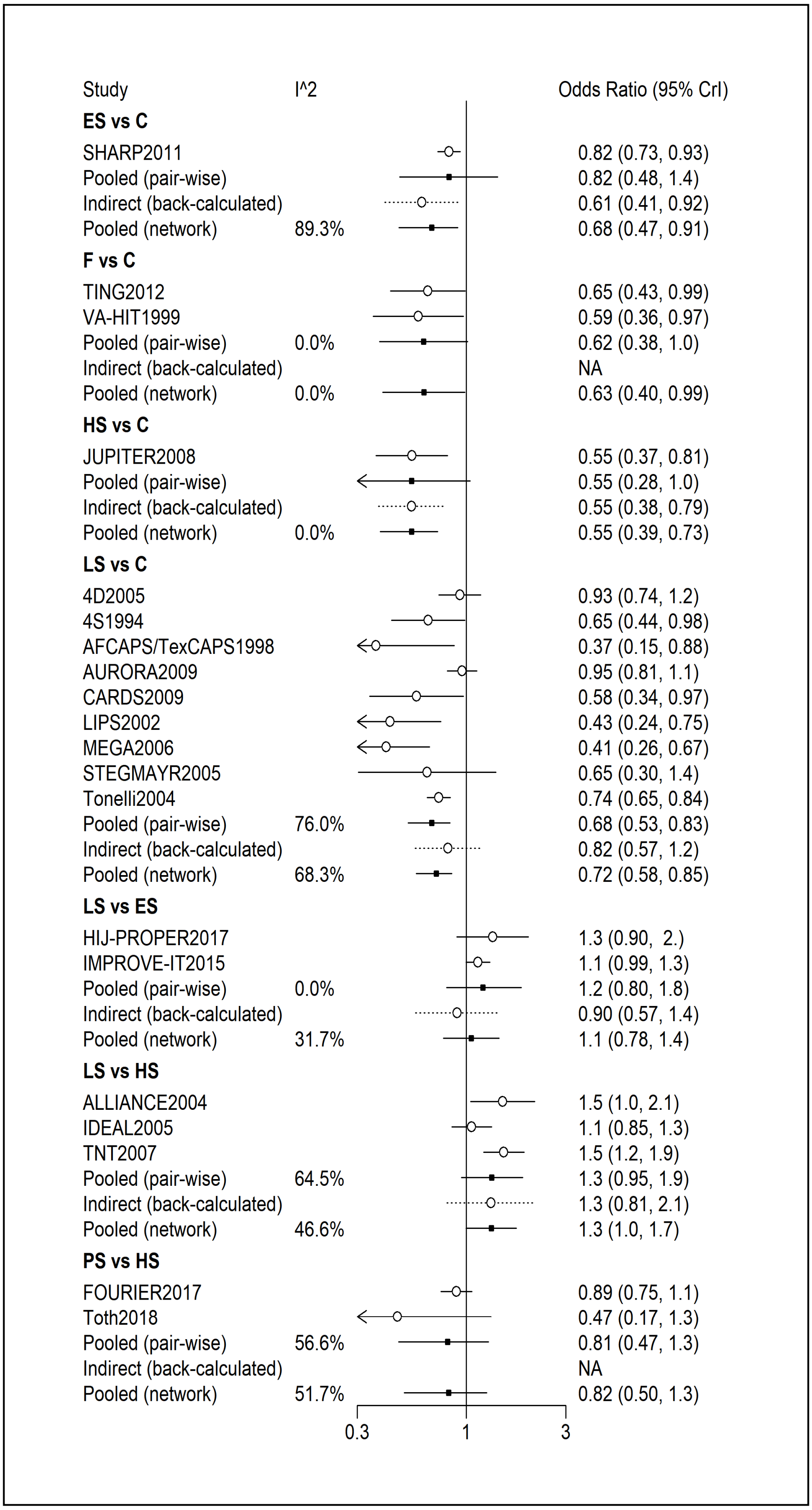
Study
I^2
Odds Ratio (95% CrI)
ES vs C
SHARP2011 0.82 (0.73, 0.93)
Pooled (pair-wise) 0.82 (0.48, 1.4)
Indirect (back-calculated) 0.61 (0.41, 0.92)
Pooled (network) 89.3% 0.68 (0.47, 0.91)
F vs C
TING2012 0.65 (0.43, 0.99)
VA-HIT1999 0.59 (0.36, 0.97)
Pooled (pair-wise) 0.0% 0.62 (0.38, 1.0)
Indirect (back-calculated) NA
Pooled (network) 0.0% 0.63 (0.40, 0.99)
HS vs C
JUPITER2008 0.55 (0.37, 0.81)
Pooled (pair-wise) 0.55 (0.28, 1.0)
Indirect (back-calculated) 0.55 (0.38, 0.79)
Pooled (network) 0.0% 0.55 (0.39, 0.73)
LS vs C
4D2005 0.93 (0.74, 1.2)
4S1994 0.65 (0.44, 0.98)
AFCAPS/TexCAPS1998 0.37 (0.15, 0.88)
AURORA2009 0.95 (0.81, 1.1)
CARDS2009 0.58 (0.34, 0.97)
LIPS2002 0.43 (0.24, 0.75)
MEGA2006 0.41 (0.26, 0.67)
STEGMAYR2005 0.65 (0.30, 1.4)
Tonelli2004 0.74 (0.65, 0.84)
Pooled (pair-wise) 76.0% 0.68 (0.53, 0.83)
Indirect (back-calculated) 0.82 (0.57, 1.2)
Pooled (network) 68.3% 0.72 (0.58, 0.85)
LS vs ES
HIJ-PROPER2017 1.3 (0.90, 2.)
IMPROVE-IT2015 1.1 (0.99, 1.3)
Pooled (pair-wise) 0.0% 1.2 (0.80, 1.8)
Indirect (back-calculated) 0.90 (0.57, 1.4)
Pooled (network) 31.7% 1.1 (0.78, 1.4)
LS vs HS
ALLIANCE2004 1.5 (1.0, 2.1)
IDEAL2005 1.1 (0.85, 1.3)
TNT2007 1.5 (1.2, 1.9)
Pooled (pair-wise) 64.5% 1.3 (0.95, 1.9)
Indirect (back-calculated) 1.3 (0.81, 2.1)
Pooled (network) 46.6% 1.3 (1.0, 1.7)
PS vs HS
FOURIER2017 0.89 (0.75, 1.1)
Toth2018 0.47 (0.17, 1.3)
Pooled (pair-wise) 56.6% 0.81 (0.47, 1.3)
Indirect (back-calculated) NA
Pooled (network) 51.7% 0.82 (0.50, 1.3)
0.3
1
3

B

S6.2.5. Trace plots, density plots and Brooks-Gelman-Rubin diagnosis plots

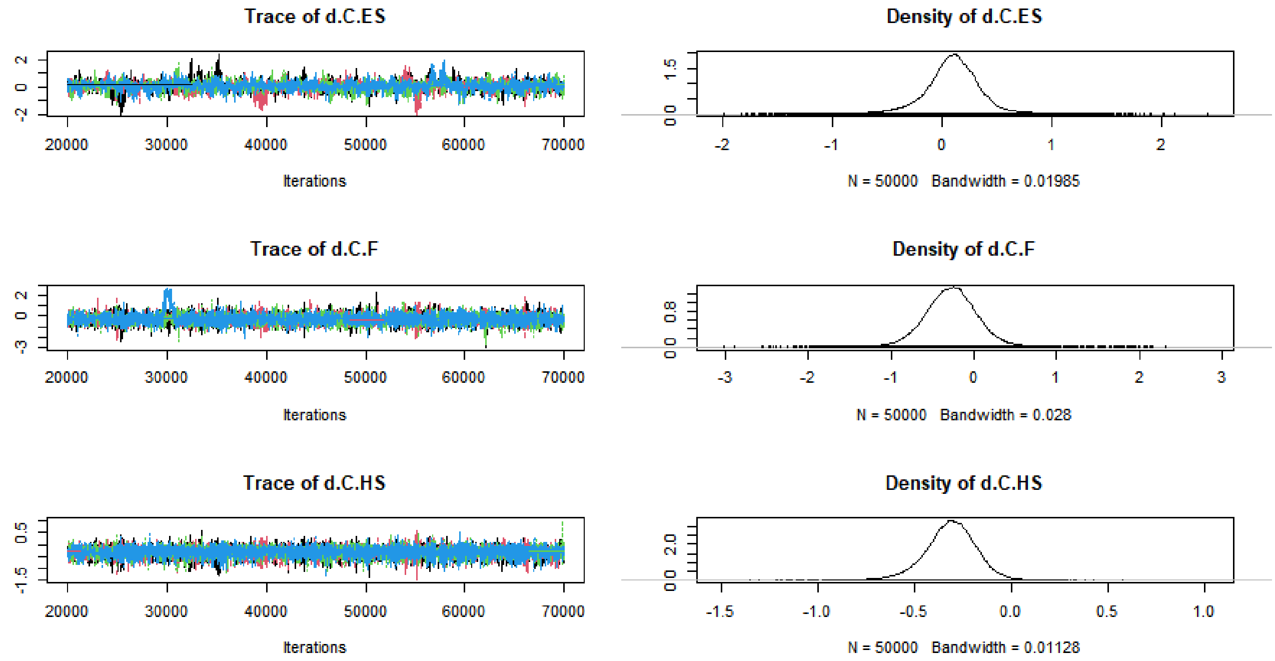

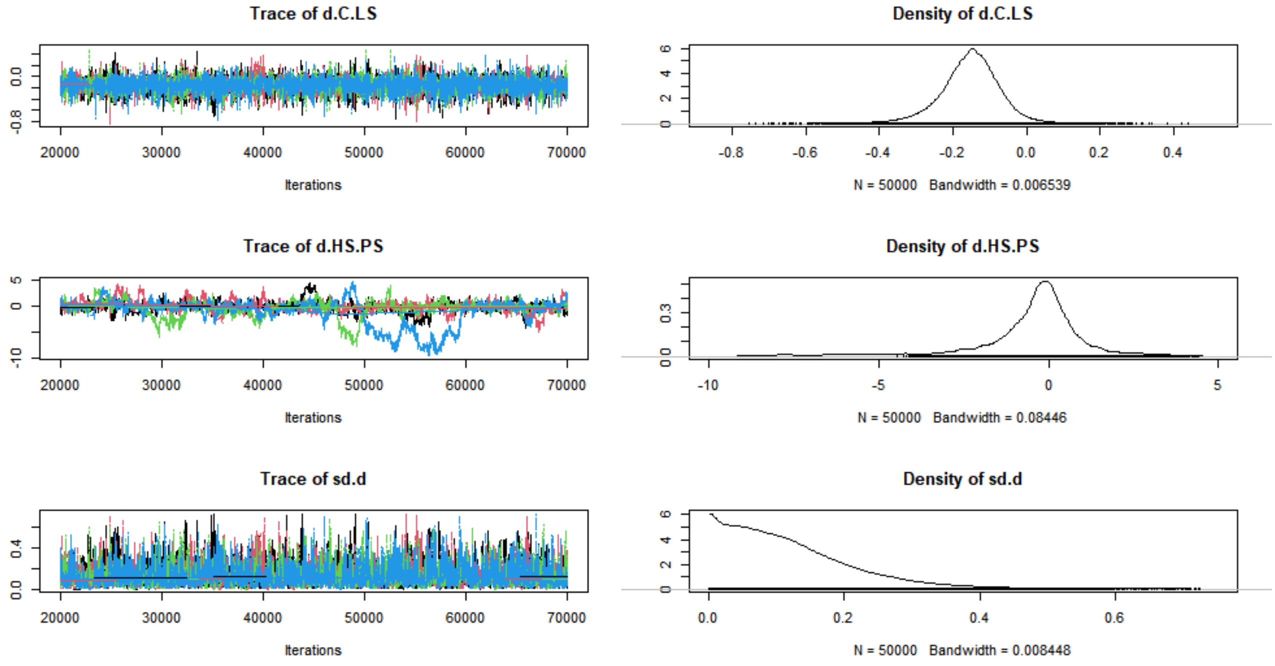

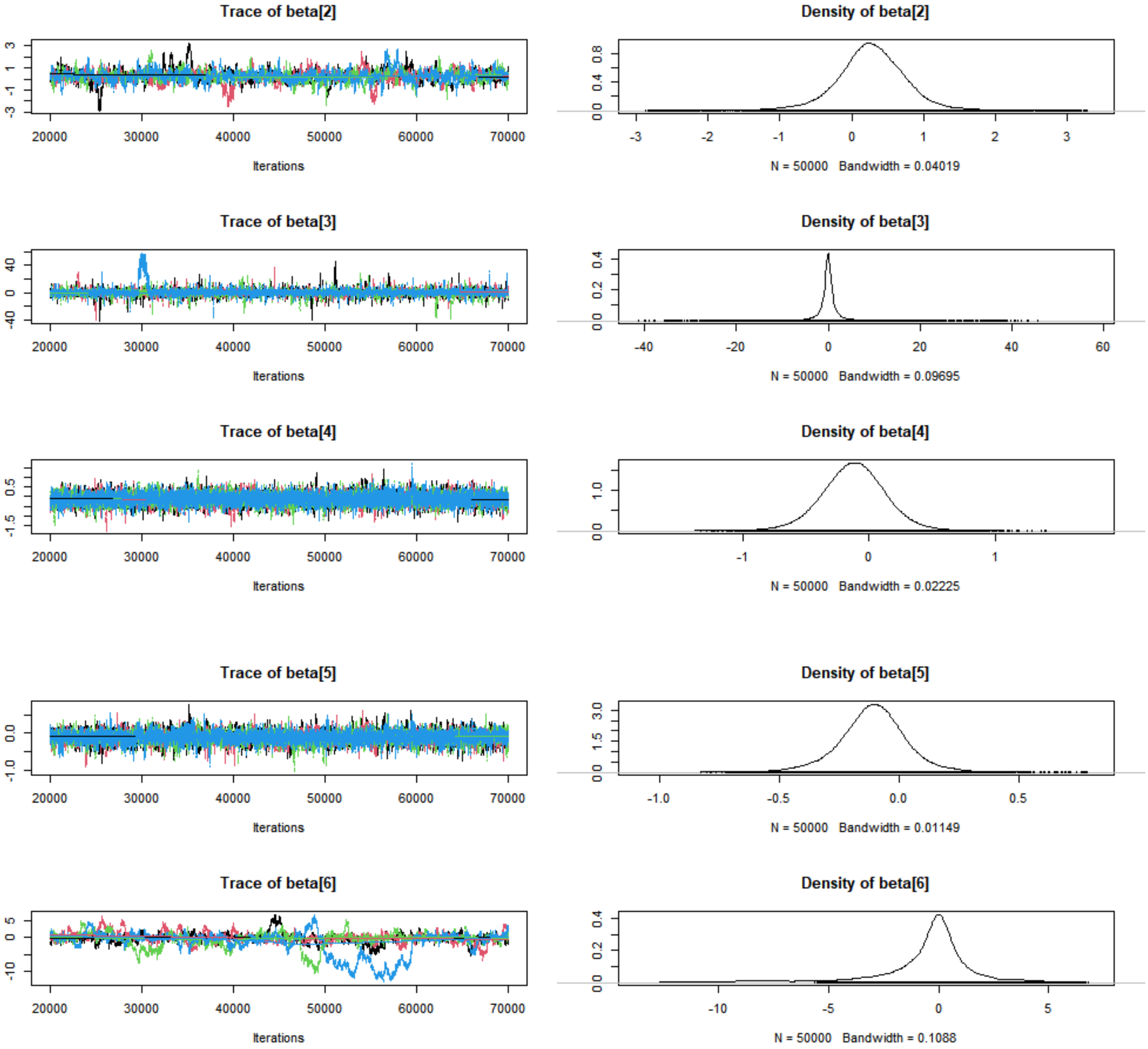

Trace of beta[2]
Iterations
Density of beta[2]
N = 50000 Bandwidth = 0.04019
Trace of beta[3]
Iterations
Density of beta[3]
N = 50000 Bandwidth = 0.09695
Trace of beta[4]
Iterations
Density of beta[4]
N = 50000 Bandwidth = 0.02225
Trace of beta[5]
Iterations
Density of beta[5]
N = 50000 Bandwidth = 0.01149
Trace of beta[6]
Iterations
Density of beta[6]
N = 50000 Bandwidth = 0.1088

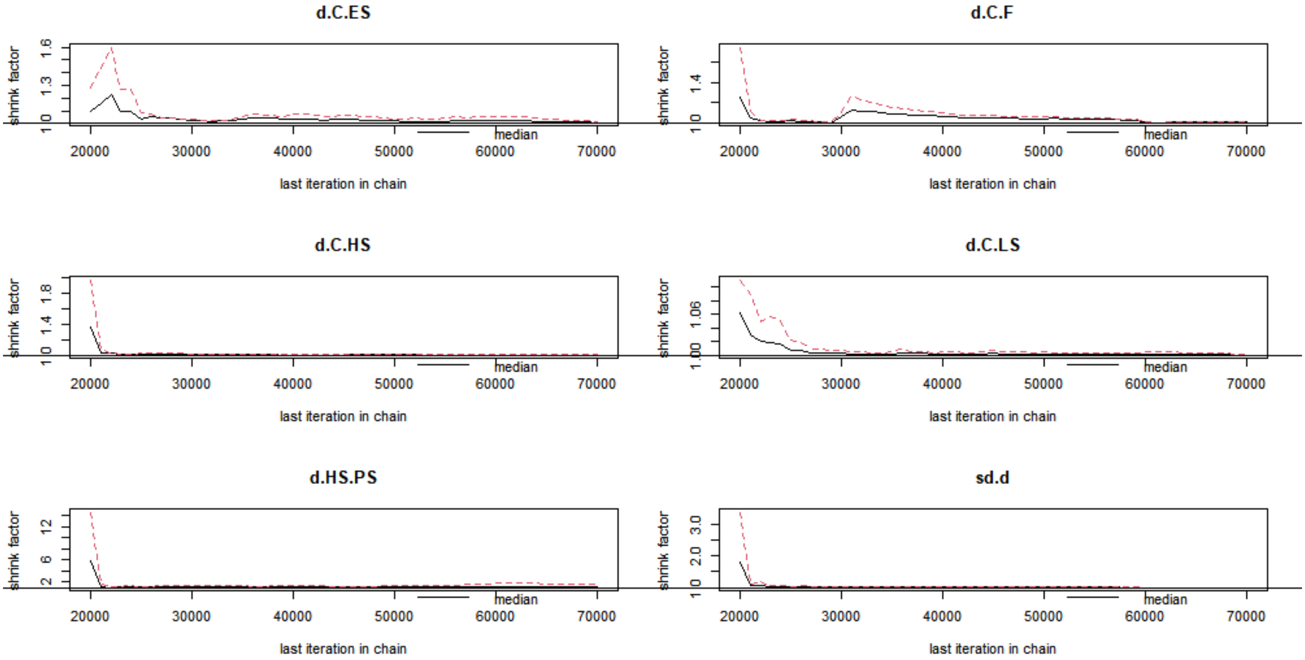
d.C.ES
d.C.F
d.C.HS
d.C.LS
d.HS.PS
sd.d
shrink factor
median
last iteration in chain
20000
30000
40000
50000
60000
70000

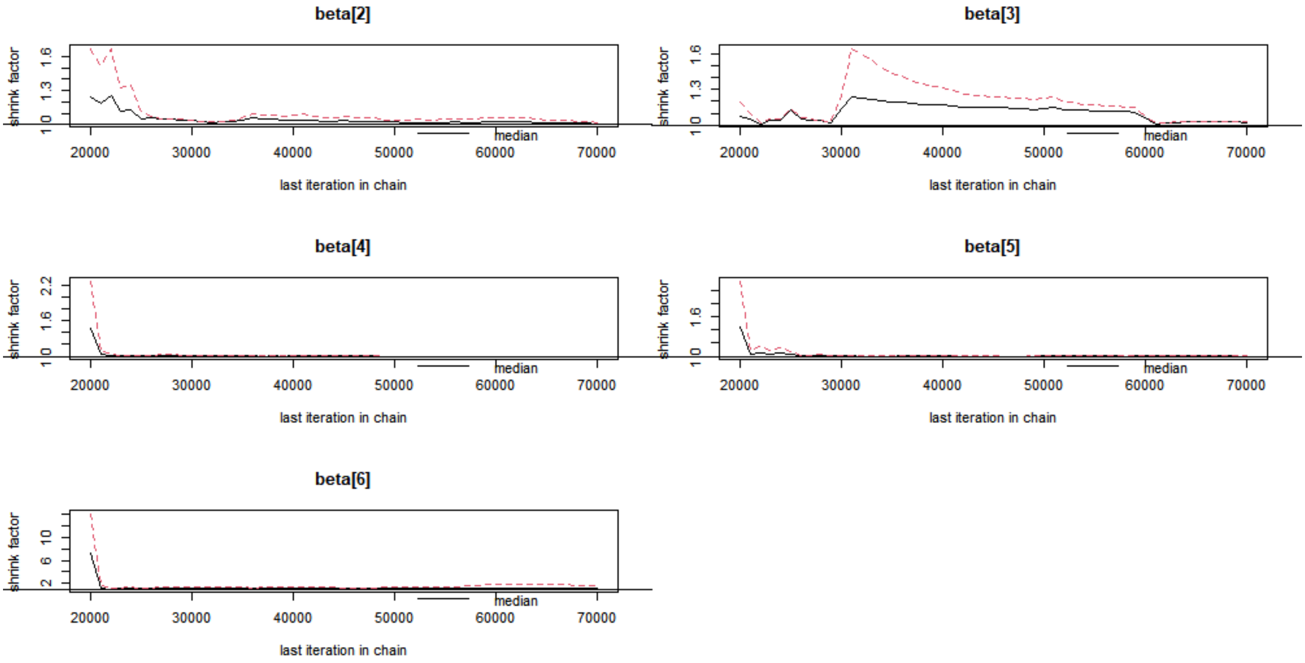
beta[2]
beta[3]
beta[4]
beta[5]
beta[6]
shrink factor
median
last iteration in chain
20000
30000
40000
50000
60000
70000

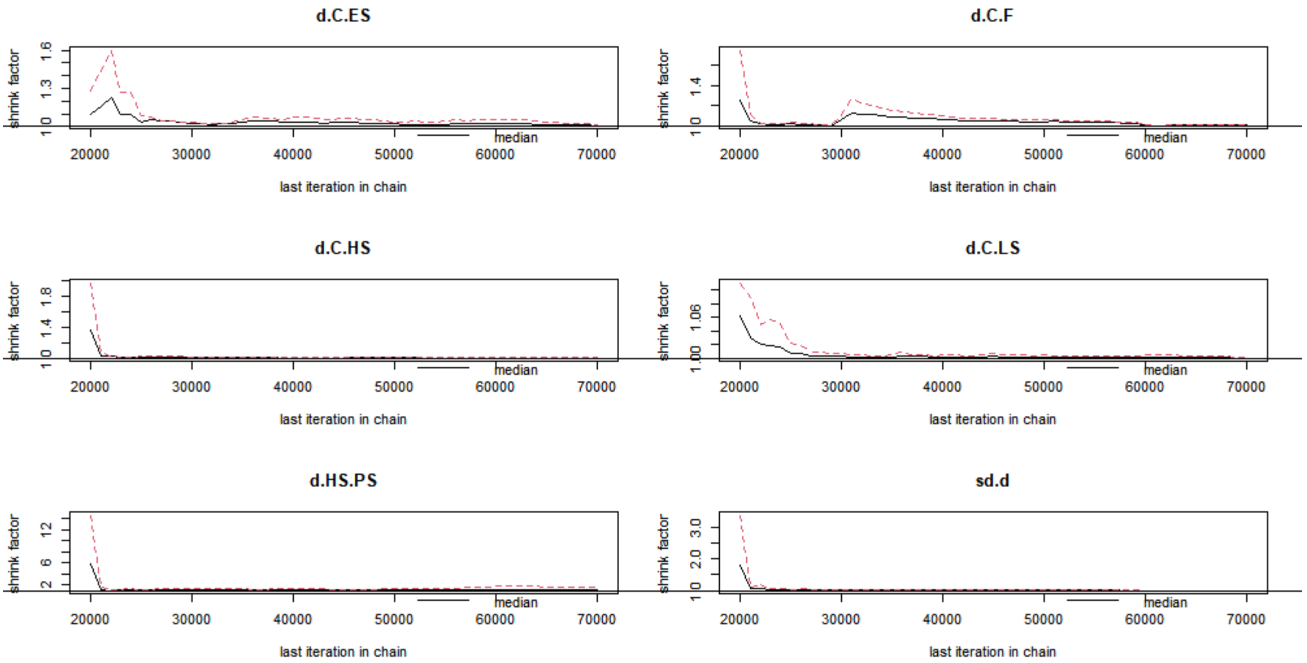

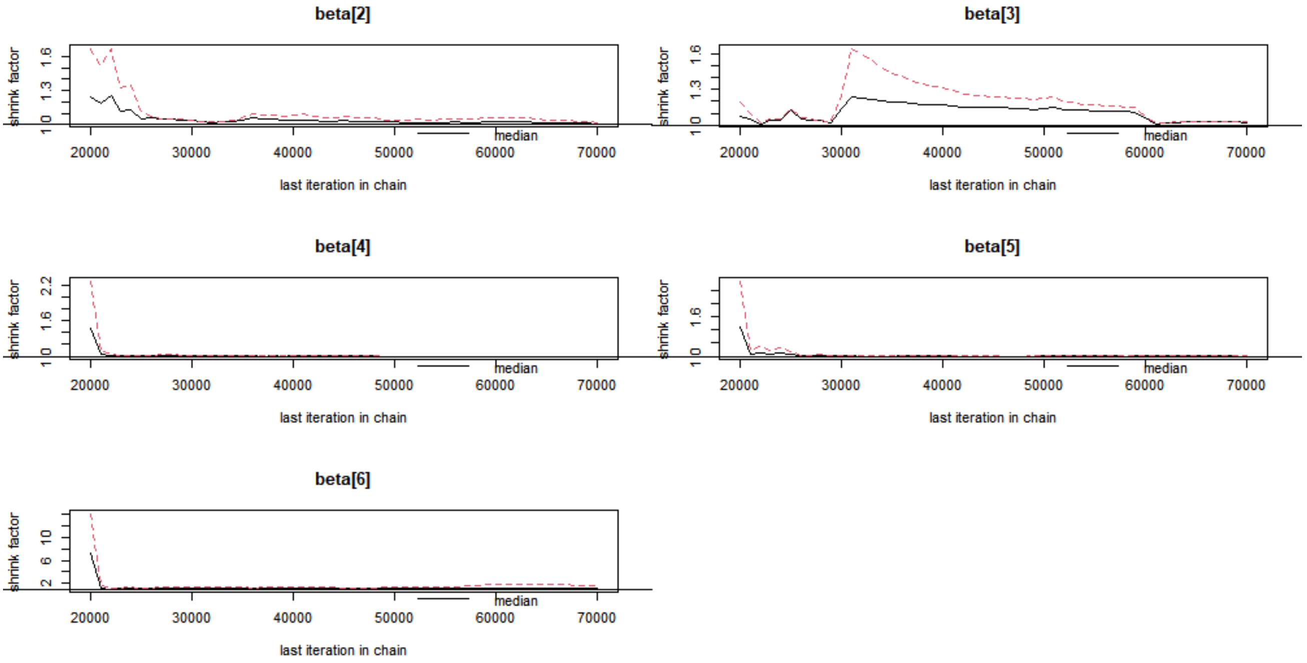

Potential scale reduction factors:

Point est. Upper C.I.		
d.C.ES	1.01	1.02
d.C.F	1.00	1.00
d.C.HS	1.00	1.00

d.C.LS	1.00	1.00
d.HS.PS	1.22	1.62
sd.d	1.00	1.00
beta[2]	1.01	1.02
beta[3]	1.02	1.03
beta[4]	1.00	1.00
beta[5]	1.00	1.00
beta[6]	1.22	1.65
Multivariate psrf=1.09		

S6.2.6. The rank probabilities of each treatment.

R / I	1	2	3	4	5	6
C	0.000000	0.000000	0.000000	0.001000	0.029625	0.969375
ES	0.027375	0.066875	0.257750	0.401375	0.240250	0.006375
F	0.146125	0.189875	0.318750	0.140875	0.183875	0.020500
HS	0.099625	0.569250	0.264875	0.056625	0.009250	0.000375
LS	0.001000	0.008250	0.097375	0.371875	0.520875	0.000625
PS	0.725875	0.165750	0.061250	0.028250	0.016125	0.002750

I: interventions; R: rank. □ **Rank the highest**

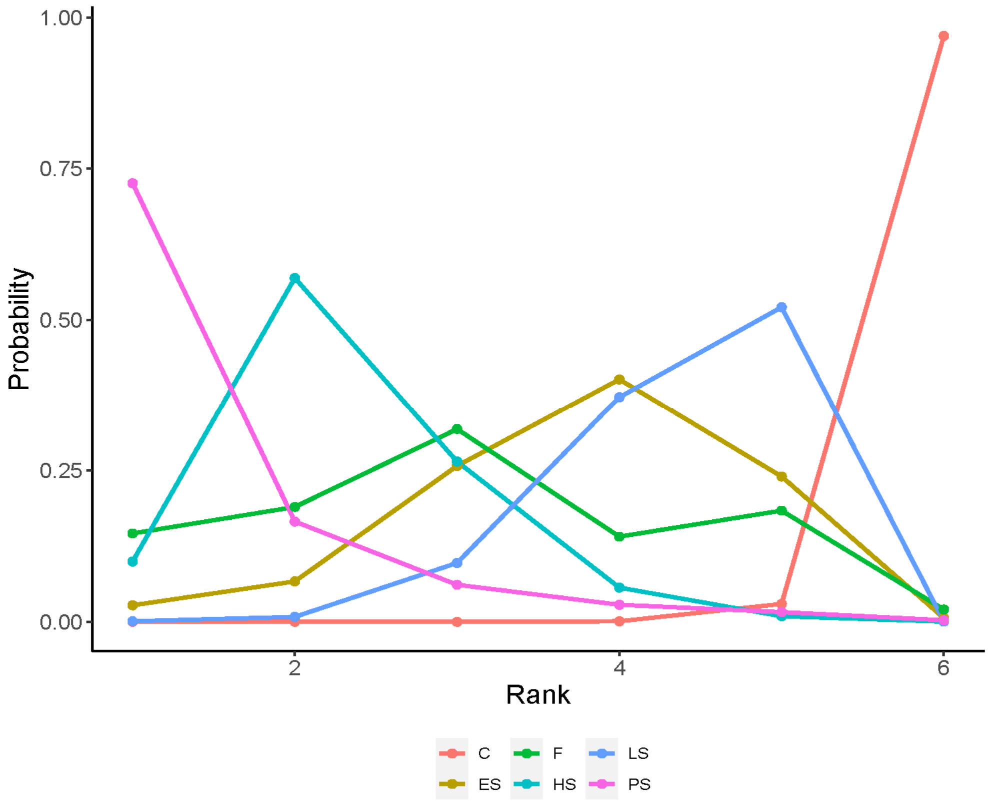

S6.2.7. The regression analysis for baseline LDL-c level

Quantiles for each variable:

	2.5%	25%	50%	75%	97.5%
d.C.ES	-0.80	-0.51	-0.39	-0.28	-0.05
d.C.F	-1.07	-0.65	-0.46	-0.27	0.19
d.C.HS	-0.88	-0.63	-0.53	-0.44	-0.26
d.C.LS	-0.49	-0.32	-0.25	-0.19	-0.10
d.HS.PS	-2.36	-0.98	-0.48	-0.07	0.67
sd.d	0.02	0.11	0.17	0.24	0.40

beta[2]	-1.03	-0.54	-0.30	-0.07	0.39
beta[3]	-2.49	-0.53	0.07	0.71	2.94
beta[4]	-0.69	-0.26	-0.07	0.13	0.56
beta[5]	-0.52	-0.33	-0.24	-0.14	0.11
beta[6]	-3.72	-1.31	-0.50	0.08	1.20

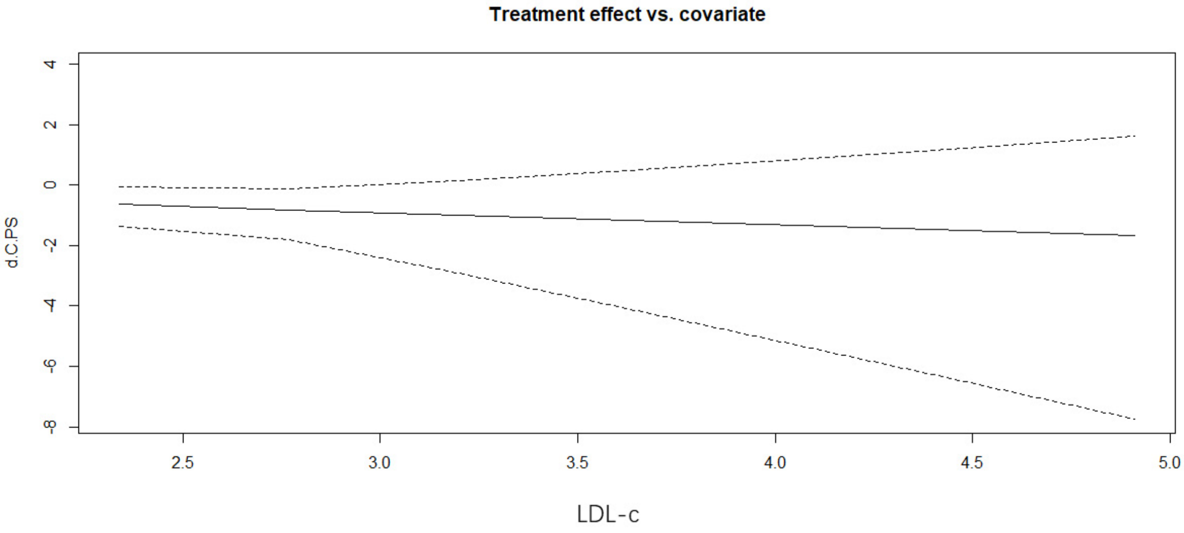

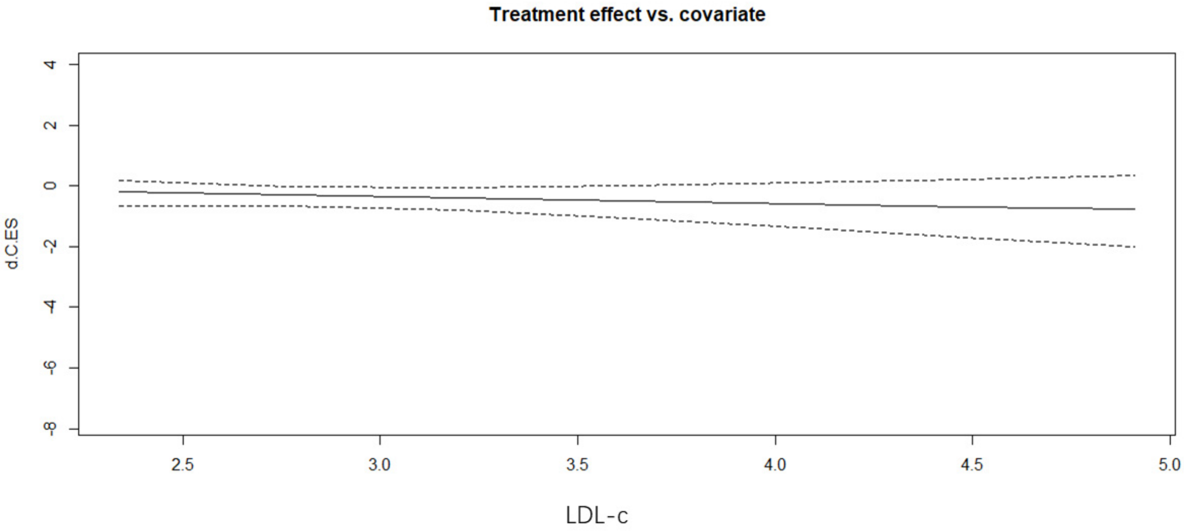

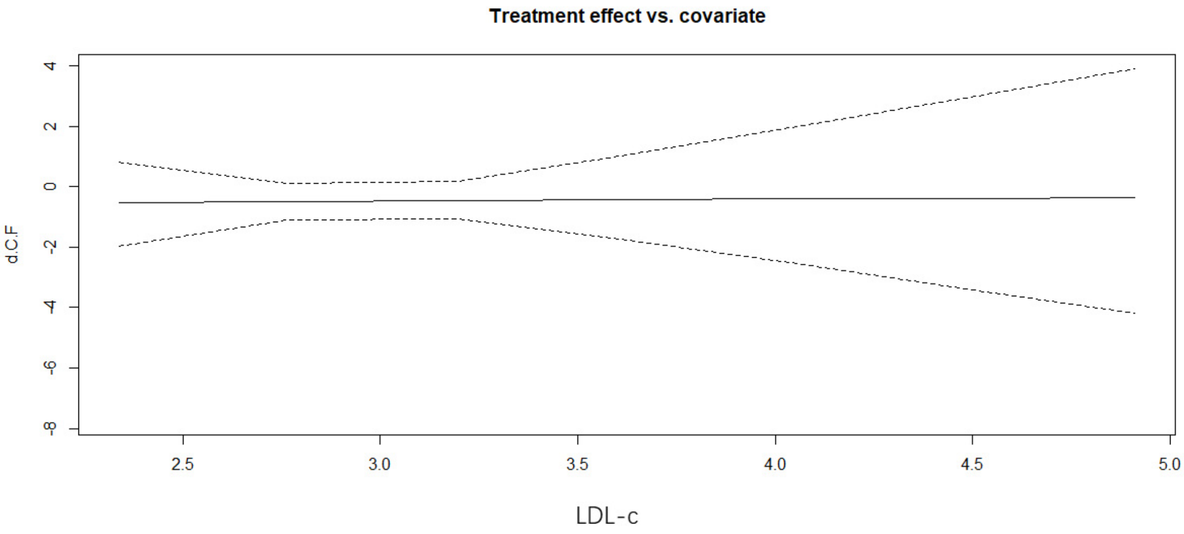
Treatment effect vs. covariate
d.C.F
4
2
0
-2
-4
-6
-8
2.5
3.0
3.5
4.0
4.5
5.0
LDL-c

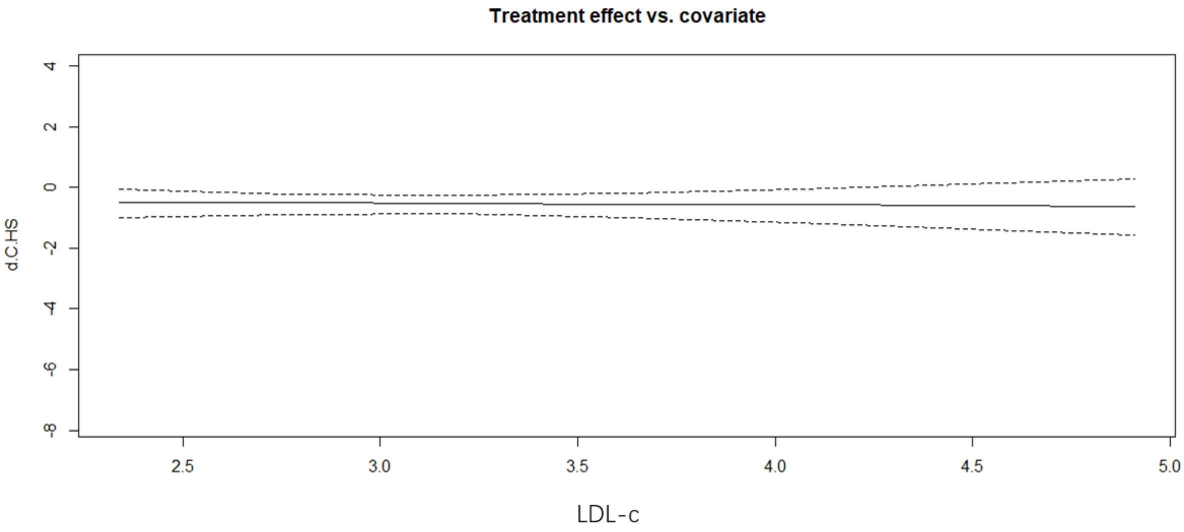
Treatment effect vs. covariate
d.C.HS
4
2
0
-2
-4
-6
-8
2.5
3.0
3.5
4.0
4.5
5.0
LDL-c

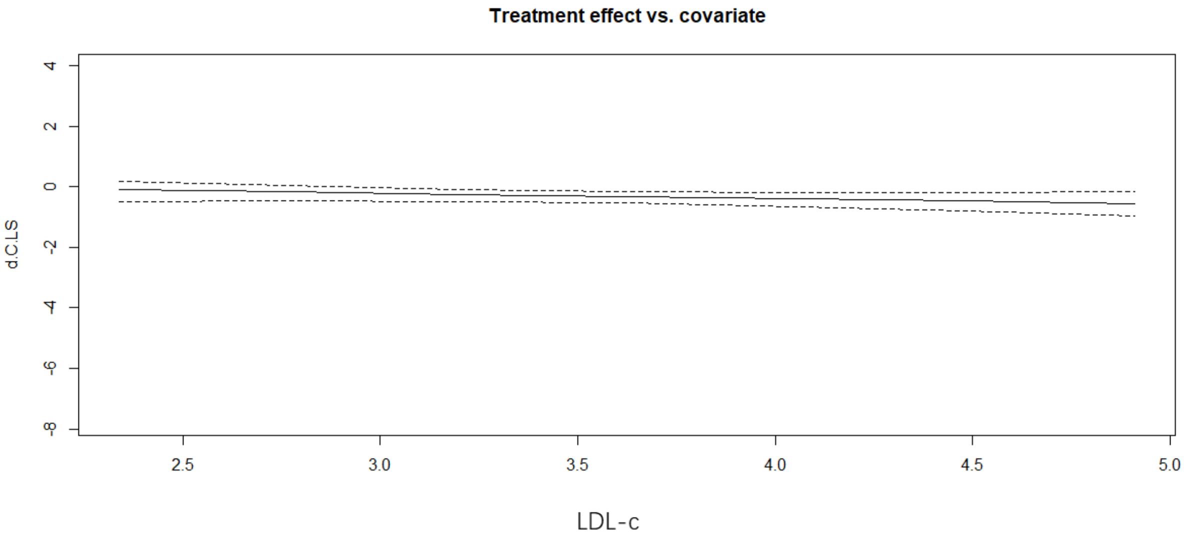
Treatment effect vs. covariate
d.C.LS
4
2
0
-2
-4
-6
-8
2.5
3.0
3.5
4.0
4.5
5.0
LDL-c

S6.2.8. Network meta-analysis was performed with the frequentist model

1). NMA for all the trials.

C	1.444 (1.138, 1.832)	1.598 (1.079, 2.367)	1.801 (1.404, 2.310)	1.364 (1.175, 1.583)	2.158 (1.410, 3.304)
0.693 (0.546, 0.879)	ES	1.107 (0.699, 1.752)	1.247 (0.913, 1.704)	0.944 (0.751, 1.188)	1.495 (0.938, 2.380)
0.626 (0.422, 0.927)	0.903 (0.571, 1.430)	F	1.127 (0.708, 1.794)	0.853 (0.560, 1.299)	1.350 (0.756, 2.410)
0.555 (0.433, 0.712)	0.802 (0.587, 1.095)	0.887 (0.557, 1.413)	HS	0.757 (0.608, 0.942)	1.198 (0.848, 1.693)
0.733 (0.632, 0.851)	1.059 (0.842, 1.332)	1.172 (0.770, 1.784)	1.321 (1.062, 1.644)	LS	1.583 (1.051, 2.382)
0.463 (0.303, 0.709)	0.669 (0.420, 1.066)	0.741 (0.415, 1.322)	0.835 (0.591, 1.179)	0.632 (0.420, 0.951)	PS

2). NMA after excluding trials with a high risk of bias.

C	1.341 (1.054, 1.706)	1.598 (1.090, 2.342)	1.690 (1.304, 2.191)	1.309 (1.125, 1.522)	2.011 (1.326, 3.051)
0.746 (0.586, 0.949)	ES	1.192 (0.759, 1.872)	1.260 (0.908, 1.750)	0.976 (0.767, 1.242)	1.500 (0.944, 2.382)
0.626 (0.427, 0.917)	0.839 (0.534, 1.318)	F	1.058 (0.666, 1.679)	0.819 (0.543, 1.235)	1.259 (0.715, 2.216)
0.592 (0.456, 0.767)	0.794 (0.571, 1.102)	0.945 (0.596, 1.501)	HS	0.774 (0.613, 0.978)	1.190 (0.859, 1.649)
0.764 (0.657, 0.889)	1.025 (0.805, 1.304)	1.221 (0.810, 1.842)	1.292 (1.022, 1.632)	LS	1.537 (1.029, 2.296)
0.497 (0.328, 0.754)	0.667 (0.420, 1.059)	0.794 (0.451, 1.398)	0.840 (0.607, 1.164)	0.651 (0.436, 0.972)	PS

S6.2.9. Sensitivity analysis by excluding studies enrolling a large number of patients with cardiovascular disease

Study or Subgroup	Experimental Events	Experimental Total	Control Events	Control Total	Weight	Odds Ratio M-H, Random, 95% CI
**1.2.2 ES vs C**						
SHARP2011	526	4650	619	4620	20.0%	0.82 [0.73, 0.93]
**Subtotal (95% CI)**		**4650**		**4620**	**20.0%**	**0.82 [0.73, 0.93]**
Total events	526		619			
Heterogeneity: Not applicable						
Test for overall effect: Z = 3.05 (P = 0.002)						
**1.2.4 F vs C**						
TING2012	57	295	60	224	10.1%	0.65 [0.43, 0.99]
**Subtotal (95% CI)**		**295**		**224**	**10.1%**	**0.65 [0.43, 0.99]**
Total events	57		60			
Heterogeneity: Not applicable						
Test for overall effect: Z = 2.01 (P = 0.04)						
**1.2.5 HS vs C**						
JUPITER2008	40	1638	71	1629	10.6%	0.55 [0.37, 0.81]
**Subtotal (95% CI)**		**1638**		**1629**	**10.6%**	**0.55 [0.37, 0.81]**
Total events	40		71			
Heterogeneity: Not applicable						
Test for overall effect: Z = 2.98 (P = 0.003)						
**1.2.6 LS vs C**						
4D2005	226	619	243	636	16.2%	0.93 [0.74, 1.17]
AFCAPS/TexCAPS2009	8	145	21	159	3.6%	0.38 [0.16, 0.90]
AURORA2009	396	1389	408	1384	18.7%	0.95 [0.81, 1.12]
CARDS2009	25	482	42	488	7.8%	0.58 [0.35, 0.97]
MEGA2006	25	1471	60	1507	8.6%	0.42 [0.26, 0.67]
Stegmayr2005	49	70	57	73	4.4%	0.65 [0.31, 1.39]
**Subtotal (95% CI)**		**4176**		**4247**	**59.3%**	**0.69 [0.51, 0.92]**
Total events	729		831			
Heterogeneity: Tau² = 0.08; Chi² = 17.21, df = 5 (P = 0.004); I² = 71%						
Test for overall effect: Z = 2.53 (P = 0.01)						
**Total (95% CI)**		**10759**		**10720**	**100.0%**	**0.71 [0.60, 0.85]**
Total events	1352		1581			
Heterogeneity: Tau² = 0.04; Chi² = 22.56, df = 8 (P = 0.004); I² = 65%						
Test for overall effect: Z = 3.72 (P = 0.0002)						
Test for subgroup differences: Chi² = 5.18, df = 3 (P = 0.16), I² = 42.1%						

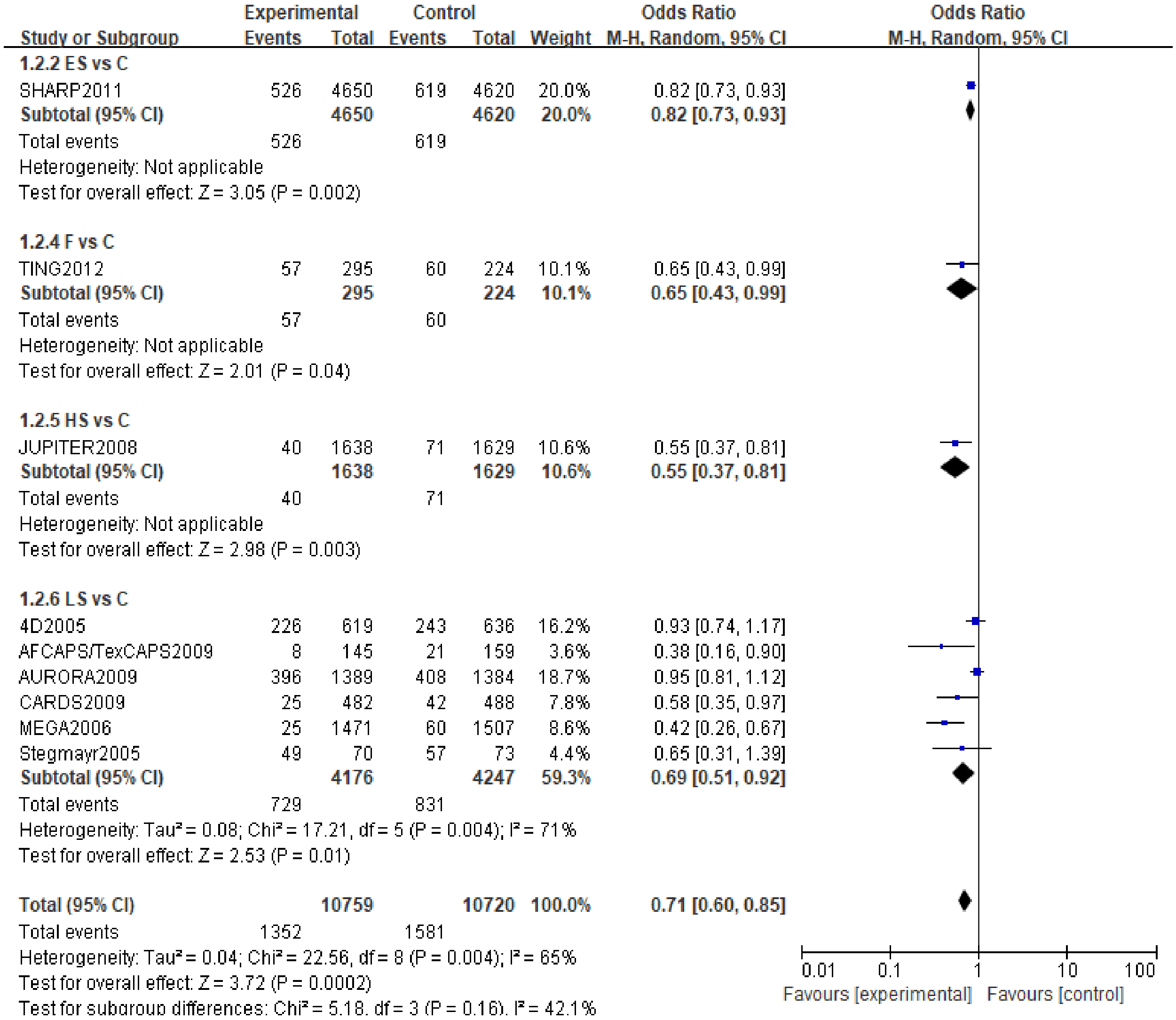

S6.3. Cardiovascular death and stroke

S6.3.1. Cardiovascular death

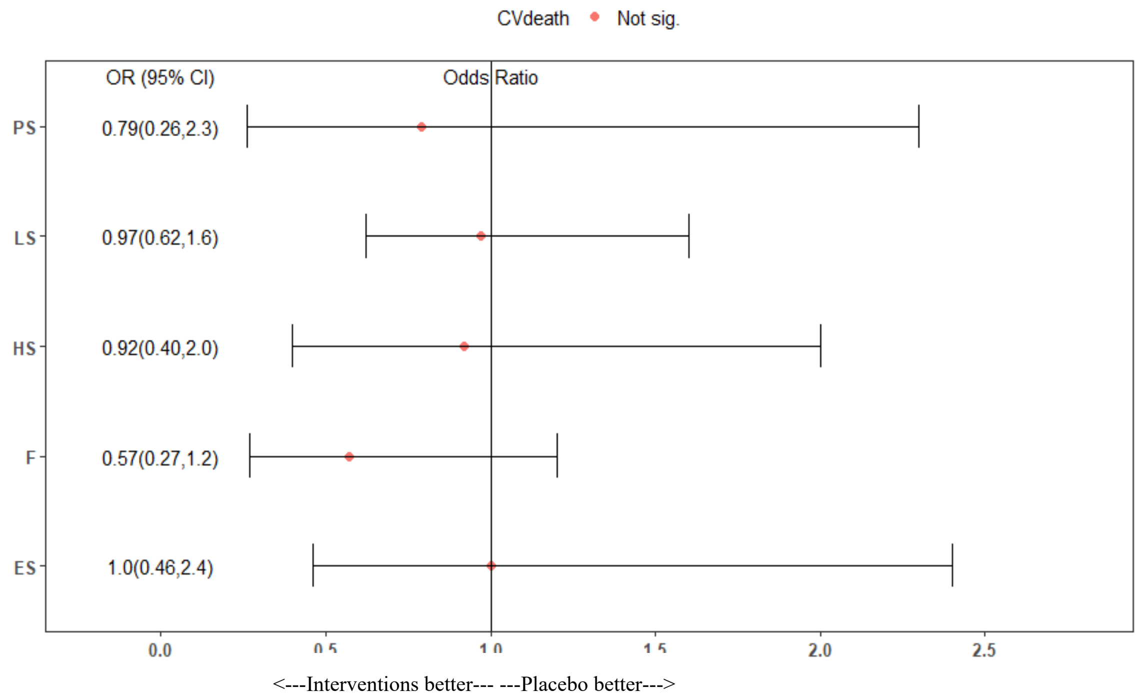

S6.3.2. Stroke

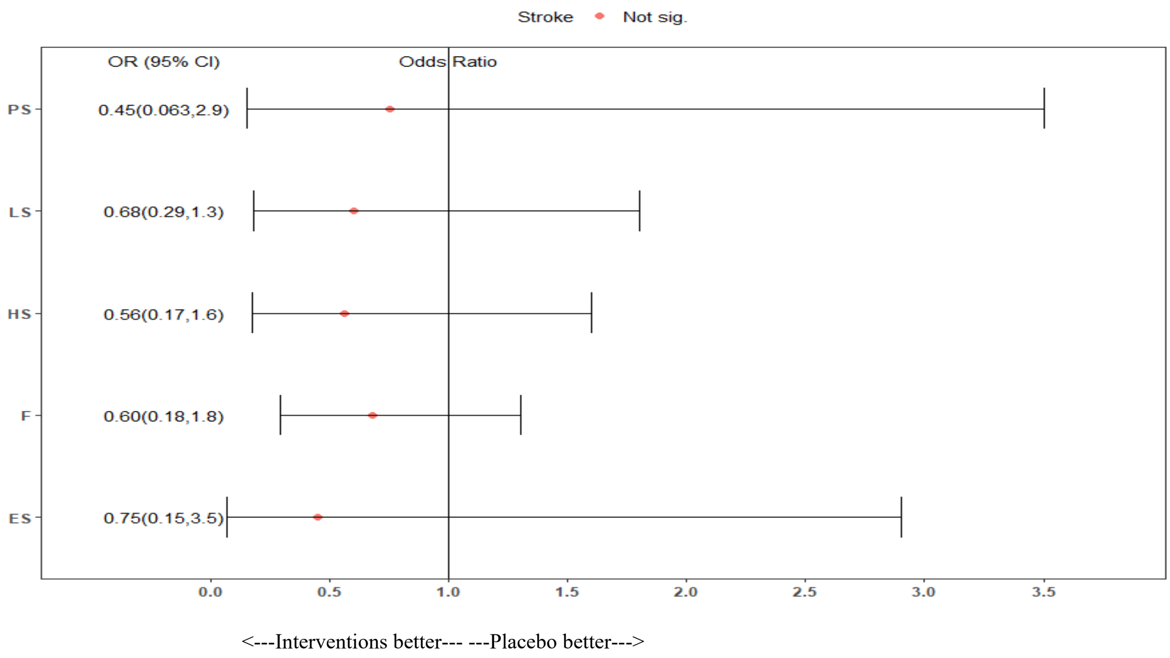

S6.4. LDL-c reduction

S6.4.1. the forest plot of NMA

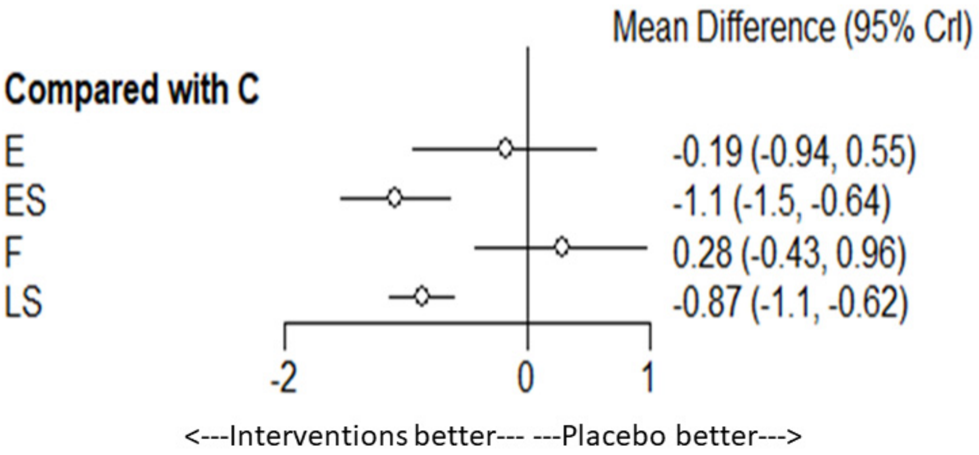

S6.4.2. The analysis of consistency (A) and heterogeneity (B).

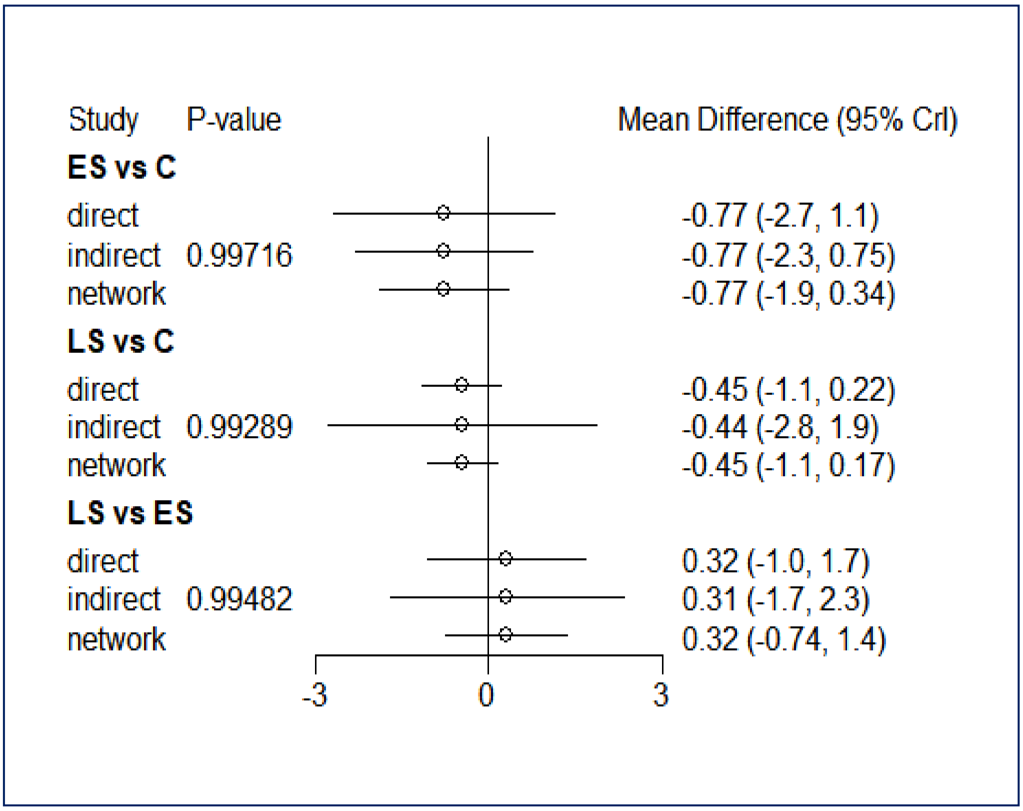

A

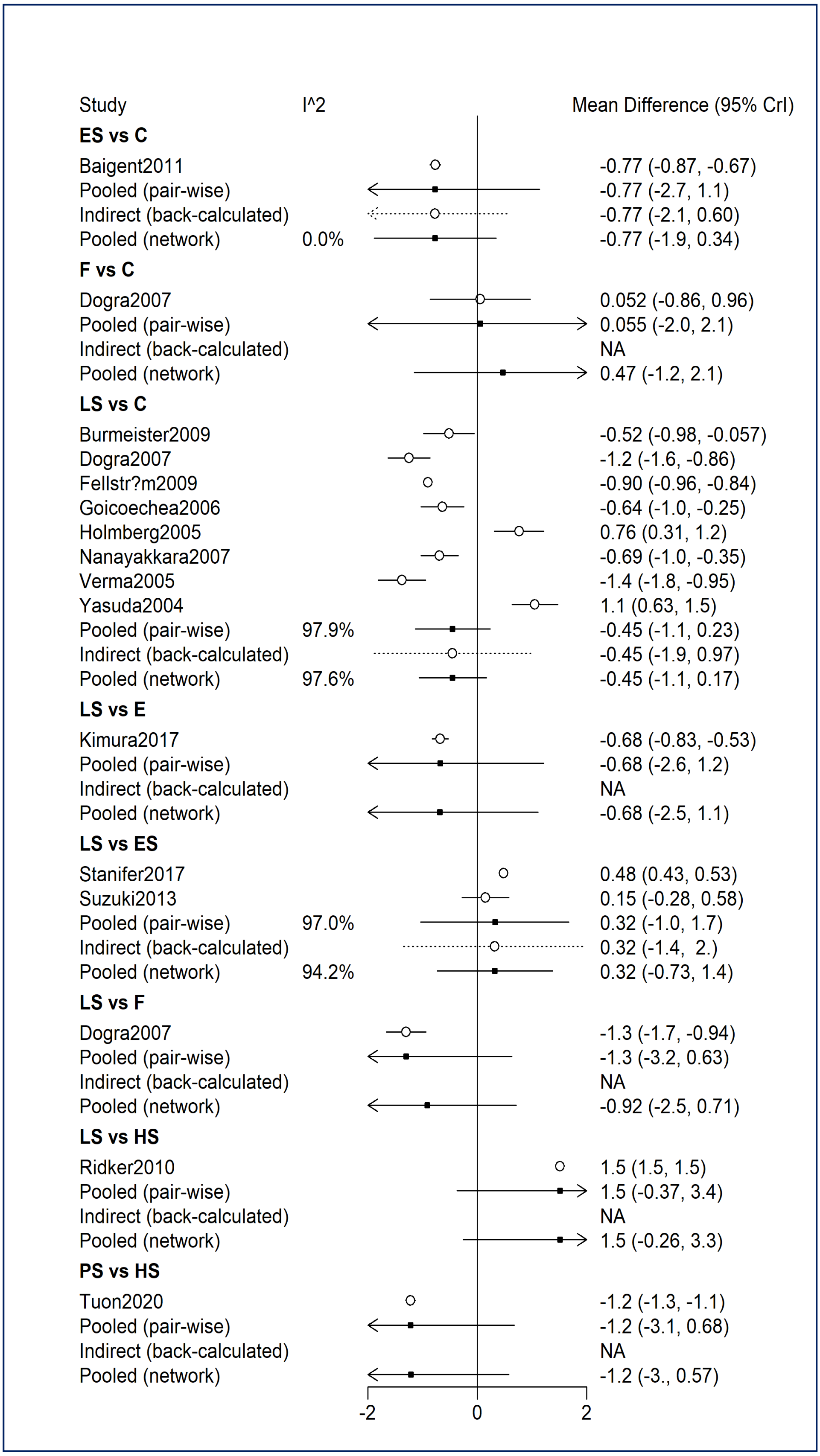
Study
I^2
Mean Difference (95% CrI)
ES vs C
Baigent2011 -0.77 (-0.87, -0.67)
Pooled (pair-wise) -0.77 (-2.7, 1.1)
Indirect (back-calculated) -0.77 (-2.1, 0.60)
Pooled (network) 0.0% -0.77 (-1.9, 0.34)
F vs C
Dogra2007 0.052 (-0.86, 0.96)
Pooled (pair-wise) 0.055 (-2.0, 2.1)
Indirect (back-calculated) NA
Pooled (network) 0.47 (-1.2, 2.1)
LS vs C
Burmeister2009 -0.52 (-0.98, -0.057)
Dogra2007 -1.2 (-1.6, -0.86)
Fellstr?m2009 -0.90 (-0.96, -0.84)
Goicoechea2006 -0.64 (-1.0, -0.25)
Holmberg2005 0.76 (0.31, 1.2)
Nanayakkara2007 -0.69 (-1.0, -0.35)
Verma2005 -1.4 (-1.8, -0.95)
Yasuda2004 1.1 (0.63, 1.5)
Pooled (pair-wise) 97.9% -0.45 (-1.1, 0.23)
Indirect (back-calculated) -0.45 (-1.9, 0.97)
Pooled (network) 97.6% -0.45 (-1.1, 0.17)
LS vs E
Kimura2017 -0.68 (-0.83, -0.53)
Pooled (pair-wise) -0.68 (-2.6, 1.2)
Indirect (back-calculated) NA
Pooled (network) -0.68 (-2.5, 1.1)
LS vs ES
Stanifer2017 0.48 (0.43, 0.53)
Suzuki2013 0.15 (-0.28, 0.58)
Pooled (pair-wise) 97.0% 0.32 (-1.0, 1.7)
Indirect (back-calculated) 0.32 (-1.4, 2.)
Pooled (network) 94.2% 0.32 (-0.73, 1.4)
LS vs F
Dogra2007 -1.3 (-1.7, -0.94)
Pooled (pair-wise) -1.3 (-3.2, 0.63)
Indirect (back-calculated) NA
Pooled (network) -0.92 (-2.5, 0.71)
LS vs HS
Ridker2010 1.5 (1.5, 1.5)
Pooled (pair-wise) 1.5 (-0.37, 3.4)
Indirect (back-calculated) NA
Pooled (network) 1.5 (-0.26, 3.3)
PS vs HS
Tuon2020 -1.2 (-1.3, -1.1)
Pooled (pair-wise) -1.2 (-3.1, 0.68)
Indirect (back-calculated) NA
Pooled (network) -1.2 (-3., 0.57)
-2
0
2

B

S6.5. Safety assessment

Study or Subgroup	Experimental Events	Experimental Total	Control Events	Control Total	Weight	Odds Ratio M-H, Random, 95% CI
**3.1.1 Muscle-related**						
ES vs. LS	40	2857	48	2828	7.2%	0.82 [0.54, 1.26]
F vs. C	57	494	37	494	6.8%	1.61 [1.04, 2.49]
HS vs. C	292	1638	303	1629	25.9%	0.95 [0.79, 1.13]
HS vs. LS	0	1888	0	1898		Not estimable
LS vs.C	370	5014	397	5104	31.5%	0.94 [0.82, 1.09]
PS vs. HS	327	3637	317	3293	28.6%	0.93 [0.79, 1.09]
**Subtotal (95% CI)**		**15528**		**15246**	**100.0%**	**0.97 [0.86, 1.09]**
Total events	1086		1102			
Heterogeneity: Tau² = 0.01; Chi² = 6.21, df = 4 (P = 0.18); I² = 36%						
Test for overall effect: Z = 0.56 (P = 0.58)						
**3.1.2 ALT/AST elevation**						
ES vs. LS	94	2915	87	2880	32.4%	1.07 [0.80, 1.44]
F vs. C	0	494	0	494		Not estimable
HS vs. C	2	1638	4	1629	4.3%	0.50 [0.09, 2.72]
HS vs. LS	22	1602	1	1605	3.2%	22.33 [3.01, 165.89]
LS vs.C	67	5014	66	5104	30.3%	1.03 [0.73, 1.46]
PS vs. HS	74	3637	54	3293	29.8%	1.25 [0.87, 1.77]
**Subtotal (95% CI)**		**15300**		**15005**	**100.0%**	**1.18 [0.81, 1.71]**
Total events	259		212			
Heterogeneity: Tau² = 0.09; Chi² = 10.49, df = 4 (P = 0.03); I² = 62%						
Test for overall effect: Z = 0.87 (P = 0.38)						

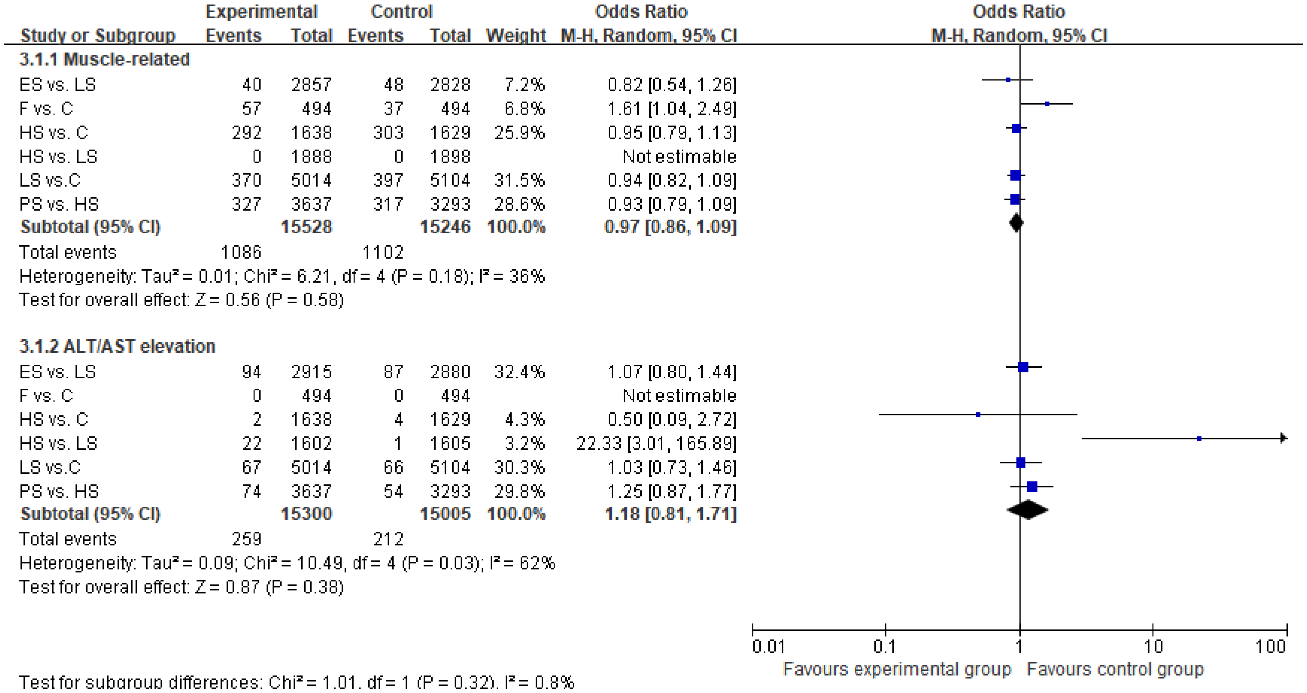

Test for subgroup differences: Chi² = 1.01, df = 1 (P = 0.32), I² = 0.8%

S7. GRADE assessment

All-cause death

Comparison	Number of RCTs	Within-study bias	Reporting bias	Indirectness	Imprecision	Heterogeneity	Incoherence	Confidence rating	Reason(s) for downgrading
C:ES	**1**	No concerns	Some concerns	No concern	Major concerns	Major concerns	No concern	Very low	["Reporting bias", "Imprecision", "Heterogeneity"]
C:F	**2**	Some concerns	Some concerns	No concern	Major concerns	No concern	No concern	Low	["Within-study bias", "Reporting bias", "Imprecision"]
C:HS	**1**	Some concerns	Some concerns	No concern	No concerns	Some concerns	No concern	Moderate	["Within-study bias", "Reporting bias", "Heterogeneity"]
C:LS	**9**	Some concerns	Some concerns	No concern	No concern	No concern	No concern	Moderate	["Within-study bias", "Reporting bias"]
C:PS	**0**	Some concerns	No concern	Some concern	No concern	No concern	No concern	Moderate	["Within-study bias", "Indirectness"]

CV events

Comparison	Number of RCTs	Within-study bias	Reporting bias	Indirectness	Imprecision	Heterogeneity	Incoherence	Confidence rating	Reason(s) for downgrading
C:ES	1	Some concerns	Some concerns	No concern	No concern	Major concerns	No concern	Low	["Within-study bias", "Reporting bias", "Heterogeneity"]
C:F	2	Some concerns	Some concerns	No concern	No concern	No concern	No concern	Moderate	["Within-study bias", "Reporting bias"]
C:HS	1	Some concerns	Some concerns	No concern	No concern	No concern	No concern	Moderate	["Within-study bias", "Reporting bias"]
C:LS	9	Some concerns	Some concerns	No concern	No concern	Some concerns	No concern	Moderate	["Within-study bias", "Reporting bias", "Heterogeneity"]
C:PS	0	Some concerns	Some concerns	Some concerns	No concern	No concern	No concern	Moderate	["Within-study bias", "Reporting bias"]

Cv death

Comparison	Number of studies	Within-study bias	Reporting bias	Indirectness	Imprecision	Heterogeneity	Incoherence	Confidence rating	Reason(s) for downgrading
C:ES	1	No concern	Some concerns	No concern	Major concerns	No concern	Major concerns	Very low	["Imprecision", "Incoherence"]
C:F	2	Some concerns	Some concerns	No concern	No concerns	Major concerns	Major concerns	Low	["Heterogeneity", "Incoherence"]
C:LS	5	Some concerns	Some concerns	No concern	Major concerns	No concern	Major concerns	Very low	["Imprecision", "Incoherence"]
C:HS	0	Some concerns	Some concerns	No concern	Major concerns	No concern	Major concerns	Very low	["Imprecision", "Incoherence"]
C:PS	0	Some concerns	Some concerns	Some concerns	Major concerns	No concern	Major concerns	Very low	["Imprecision", "Incoherence"]

Stroke

Comparison	Number of studies	Within-study bias	Reporting bias	Indirectness	Imprecision	Heterogeneity	Incoherence	Confidence rating	Reason(s) for downgrading
C:ES	1	No concern	Some concerns	No concern	Major concerns	No concern	No concern	Low	["Imprecision"]
C:HS	1	Some concerns	Some concerns	No concern	Major concerns	No concern	No concern	Low	["Imprecision"]
C:LS	8	Some concerns	Some concerns	No concern	Major concerns	No concern	No concern	Low	["Imprecision"]
C:PS	0	Some concerns	Some concerns	Some concerns	Major concerns	No concern	No concern	Low	["Imprecision"]

LDL-c reduction

Comparison	Number of studies	Within-study bias	Reporting bias	Indirectness	Imprecision	Heterogeneity	Incoherence	Confidence rating	Reason(s) for downgrading
C:ES	1	Some concerns	Some concerns	No concern	No concern	Some concerns	No concern	Moderate	["Within-study bias","Reporting bias","Heterogeneity"]
C:F	1	Some concerns	Some concerns	No concern	Major concerns	No concern	No concern	Low	["Imprecision"]
C:LS	8	Some concerns	Some concerns	No concern	No concern	Some concerns	No concern	Moderate	["Within-study bias","Reporting bias","Heterogeneity"]
C:E	0	Major concerns	Some concerns	No concern	Major concerns	No concern	No concern	Very low	["Within-study bias","Imprecision"]

S 8. The confidence assessment in the results of NMA using CINeMA approach

S8.1. The confidence assessment of all-cause death

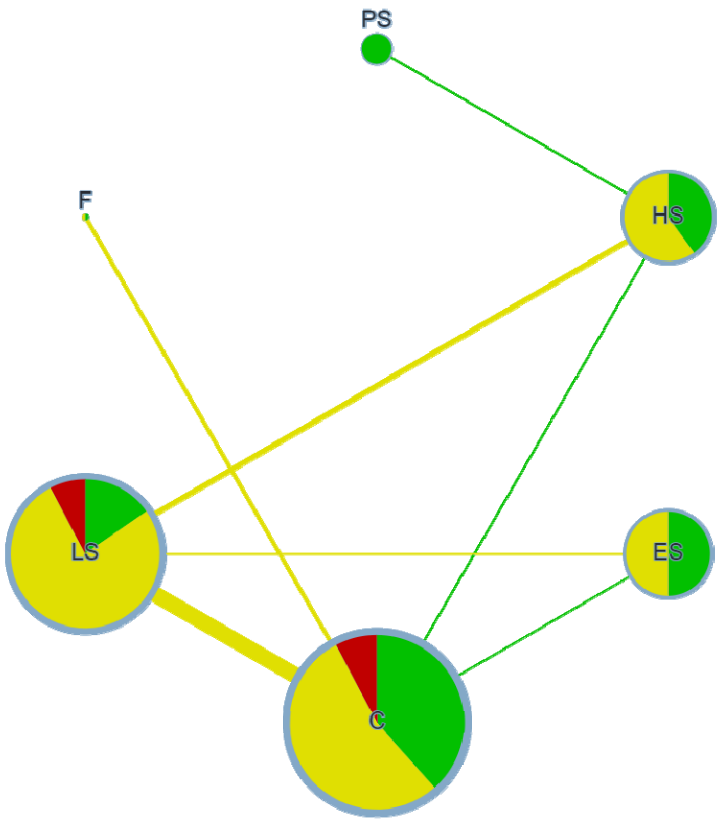

Network plot of all-cause death.

The node size represents sample size; the node color represents the risk of bias; the edge width represents the number of studies; the edge color represents the average Rob.

Per study contribution matrix.

random OR	SHARP 2011	VA-HIT 1999	TING 2012	JUPITER 2008	4D 2005	Toneil 2004	LIPS 2002	STEGMAYR 2005	4S 2009	MEGA 2006	AURORA 2009	CARDS 2009	Fassett 2010	IMPROVE-IT 2015	TNT 2007	ALLIANCE 2004	IDEAL 2005	FOURIER 2017
Mixed estimates	--	--	--	--	--	--	--	--	--	--	--	--	--	--	--	--	--	--
C:ES	75.92	0.00	0.00	0.32	2.03	3.77	0.04	0.22	0.43	0.28	4.38	0.35	0.05	11.88	0.12	0.05	0.15	0.00
C:F	0.00	35.89	64.11	0.00	0.00	0.00	0.00	0.00	0.00	0.00	0.00	0.00	0.00	0.00	0.00	0.00	0.00	0.00
C:HS	5.97	0.00	0.00	16.16	5.79	10.75	0.11	0.63	1.22	0.81	12.51	1.00	0.14	5.97	14.70	6.07	18.17	0.00
C:LS	10.	0.0	0.0	1.5	13.	24.	0.2	1.45	2.8	1.8	28.	2.3	0.3	10.	0.6	0.2	0.7	0.0

	35	0	0	9	38	83	6		2	6	88	2	2	35	0	5	4	0
Indirect estimates	--	--	--	--	--	--	--	--	--	--	--	--	--	--	--	--	--	--
C:PS	4.48	0.00	0.00	8.08	3.86	7.17	0.07	0.42	0.81	0.54	8.34	0.67	0.09	4.48	9.99	4.12	12.34	34.53

Per comparison contribution matrix.

random OR	C:ES	C:F	C:HS	C:LS	ES:LS	HS:LS	HS:PS
Mixed estimates							
C:ES	75.92	0.00	0.32	11.56	11.88	0.32	0.00
C:F	0.00	100.00	0.00	0.00	0.00	0.00	0.00
C:HS	5.97	0.00	16.16	32.96	5.97	38.93	0.00
C:LS	10.35	0.00	1.59	76.11	10.35	1.59	0.00
Indirect estimates							
C:PS	4.48	0.00	8.08	21.97	4.48	26.45	34.53

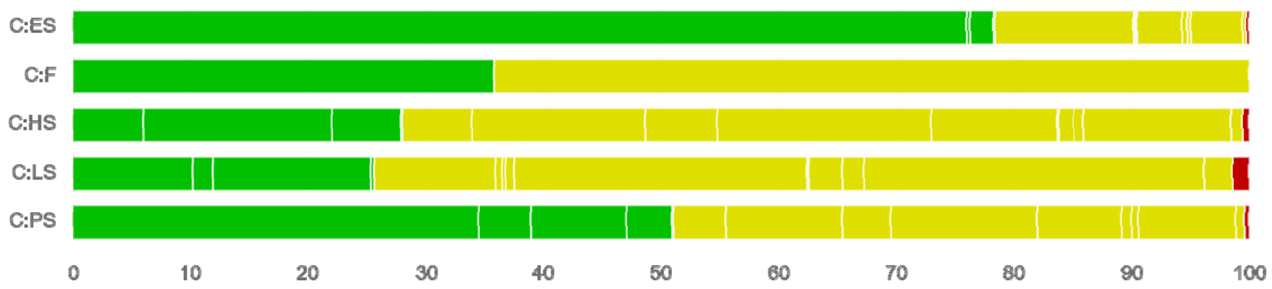

Risk of bias contributions.

The bar chart shows the contributions of each piece of study to the network estimate

CINeMA quality assessment report.

Comparison	Number of studies	Within-study bias	Reporting bias	Indirectness	Imprecision	Heterogeneity	Incoherence	Confidence rating	Reason(s) for downgrading
C:ES	1	No concerns	Low risk	No concerns	Major concerns	No concerns	Major concerns	Very low	["Imprecision","Incoherence"]
C:F	2	Some concerns	Low risk	No concerns	Major concerns	No concerns	Major concerns	Very low	["Imprecision","Incoherence"]
C:HS	1	Some concerns	Low risk	No concerns	No concerns	No concerns	Some concerns	Moderate	["Within-study bias","Incoherence"]
C:LS	9	Some concerns	Low risk	No concerns	No concerns	No concerns	Some concerns	Moderate	["Within-study bias","Incoherence"]
C:PS	0	Some concerns	Low risk	No concerns	No concerns	No concerns	Some concerns	Moderate	["Within-study bias","Incoherence"]

S8.2. the composite cardiovascular events

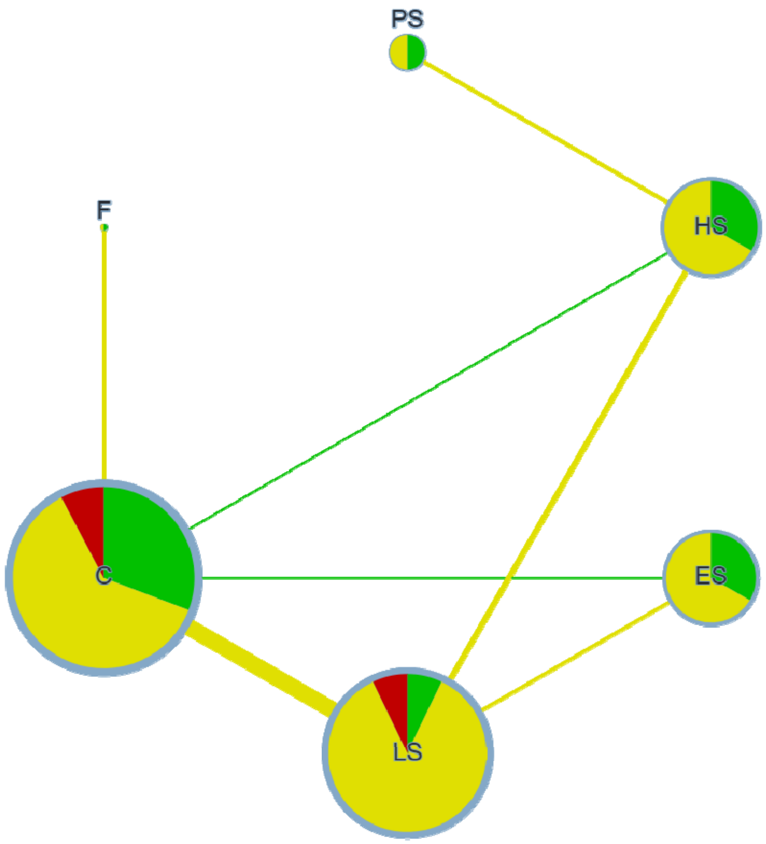

Network plot.

The node size represents sample size; the node color represents the risk of bias; the edge width represents the number of studies; the edge color represents the average Rob.

Per study contribution matrix.

random OR	SHARP 2011	VA-HIT 1999	TING 2012	JUPITER 2008	AFCAPS/TexCAPS 2010	Tonelli 2004	CARDS 2009	4D 2005	LIPS 2002	STEGMAYR 2005	4S 1994	MEGA 2006	AURORA 2009	HIJ-PROPER 2017	IMPROVE-IT 2015	TNT 2007	ALLIANCE 2004	IDEAL 2005	FOURIER 2017	Toth 2018
Mixed estimates	--	--	--	--	--	--	--	--	--	--	--	--	--	--	--	--	--	--	--	--
C:ES	47.38	0.00	0.00	1.38	0.80	5.43	1.79	4.20	1.58	0.98	2.46	2.01	5.01	8.22	17.40	0.51	0.35	0.52	0.00	0.00

C:F	0.00	43.95	56.05	0.00	0.00	0.00	0.00	0.00	0.00	0.00	0.00	0.00	0.00	0.00	0.00	0.00	0.00	0.00	0.00	0.00
C:HS	3.00	0.00	0.00	23.89	1.11	7.51	2.48	5.81	2.18	1.35	3.40	2.78	6.93	0.96	2.04	13.59	9.18	13.79	0.00	0.00
C:LS	5.49	0.00	0.00	3.51	2.70	18.36	6.07	14.19	5.33	3.30	8.31	6.79	16.94	1.76	3.73	1.30	0.88	1.32	0.00	0.00
Indirect estimates	--	--	--	--	--	--	--	--	--	--	--	--	--	--	--	--	--	--	--	--
C:PS	2.25	0.00	0.00	11.95	0.74	5.01	1.66	3.87	1.45	0.90	2.27	1.85	4.62	0.72	1.53	9.15	6.18	9.29	32.61	3.96

Per comparison contribution matrix.

random OR	C:ES	C:F	C:HS	C:LS	ES:LS	HS:LS	HS:PS
Mixed estimates							
C:ES	47.38	0.00	1.38	24.24	25.62	1.38	0.00
C:F	0.00	100.00	0.00	0.00	0.00	0.00	0.00
C:HS	3.00	0.00	23.89	33.56	3.00	36.56	0.00
C:LS	5.49	0.00	3.51	82.00	5.49	3.51	0.00
Indirect estimates							
C:PS	2.25	0.00	11.95	22.37	2.25	24.62	36.57

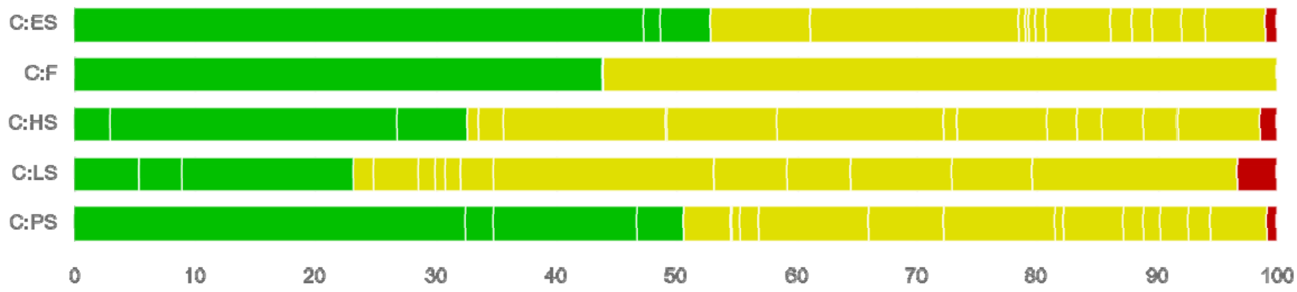

Risk of bias contributions.

The bar chart shows the contributions of each piece of study to the network estimate.

CINeMA quality assessment report.

Comparison	Number of studies	Within-study bias	Reporting bias	Indirectness	Imprecision	Heterogeneity	Incoherence	Confidence rating	Reason(s) for downgrading
C:ES	1	No concerns	Low risk	No concerns	No concerns	Major concerns	No concerns	Low	["Heterogeneity"]
C:F	2	Some concerns	Low risk	No concerns	No concerns	Major concerns	No concerns	Low	["Within-study bias","Heterogeneity"]

C:HS	1	Some concerns	Low risk	No concerns	No concerns	No concerns	No concerns	Moderate	["Within-study bias"]
C:LS	9	Some concerns	Low risk	No concerns	No concerns	Major concerns	No concerns	Low	["Within-study bias","Heterogeneity"]
C:PS	0	Some concerns	Low risk	No concerns	No concerns	No concerns	No concerns	Moderate	["Within-study bias"]

S8.3. Cardiovascular death

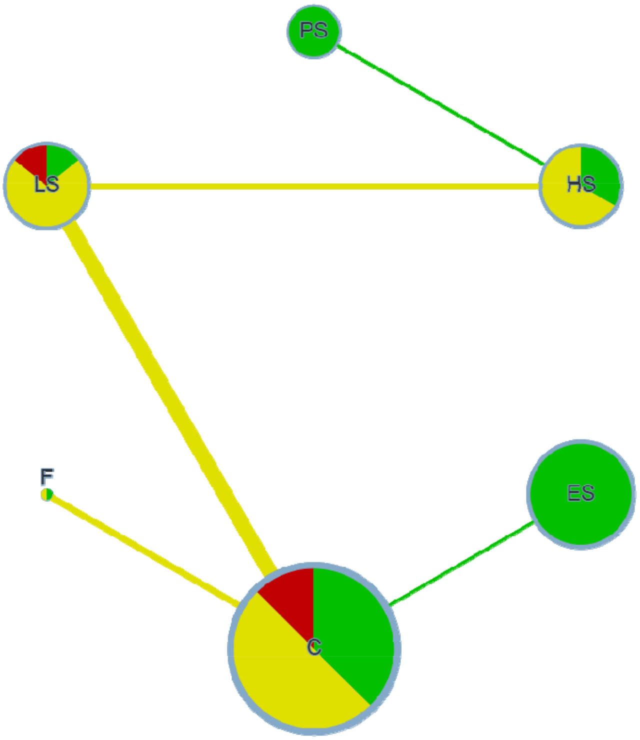

Network plot.

The node size represents sample size; the node color represents the risk of bias; the edge width represents the number of studies; the edge color represents the average Rob.

Per study contribution matrix.

random OR	SHARP 2011	VA-HIT 1999	TING 2012	Fasset t 2010	4D 2005	LIPS 2002	STEGMAYR 2005	AURORA 2009	ALLIANCE 2004	IDEAL 2005	FOURIER 2017
Mixed estimates	--	--	--	--	--	--	--	--	--	--	--
C:ES	100.00	0.00	0.00	0.00	0.00	0.00	0.00	0.00	0.00	0.00	0.00
C:F	0.00	36.52	63.48	0.00	0.00	0.00	0.00	0.00	0.00	0.00	0.00
C:LS	0.00	0.00	0.00	0.54	35.65	1.61	7.25	54.95	0.00	0.00	0.00

Indirect estimates	--	--	--	--	--	--	--	--	--	--	--
C:HS	0.00	0.00	0.00	0.27	17.82	0.81	3.63	27.47	11.79	38.21	0.00
C:PS	0.00	0.00	0.00	0.18	11.88	0.54	2.42	18.32	7.86	25.48	33.33

Per comparison contribution matrix.

random OR	C:ES	C:F	C:LS	HS:LS	HS:PS
Mixed estimates					
C:ES	100.00	0.00	0.00	0.00	0.00
C:F	0.00	100.00	0.00	0.00	0.00
C:LS	0.00	0.00	100.00	0.00	0.00
Indirect estimates					
C:HS	0.00	0.00	50.00	50.00	0.00
C:PS	0.00	0.00	33.33	33.33	33.33

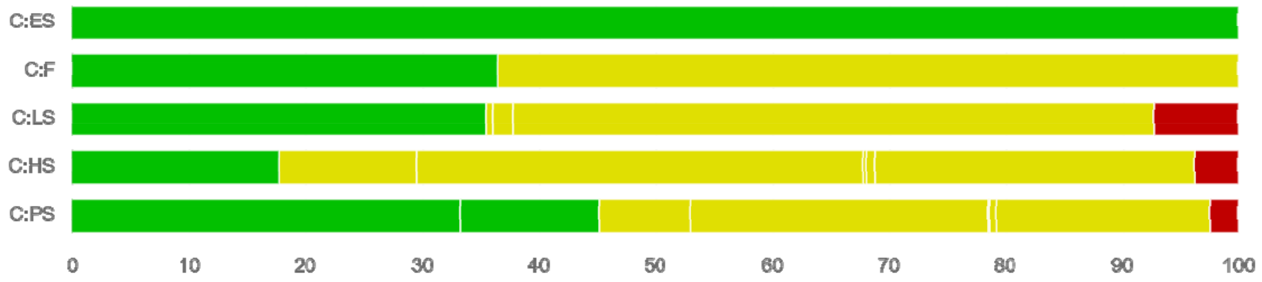

Risk of bias contributions.

The bar chart shows the contributions of each piece of study to the network estimate.

CINeMA quality assessment report.

Comparison	Number of studies	Within-study bias	Reporting bias	Indirectness	Imprecision	Heterogeneity	Incoherence	Confidence rating	Reason(s) for downgrading
C:ES	1	No concerns	Low risk	No concerns	Major concerns	No concerns	Major concerns	Very low	["Imprecision","Incoherence"]
C:F	2	Some concerns	Low risk	No concerns	No concerns	Major concerns	Major concerns	Very low	["Within-study bias","Heterogeneity","Incoherence"]
C:LS	5	Some concerns	Low risk	No concerns	Major concerns	No concerns	Major concerns	Very low	["Within-study bias","Imprecision","Incoherence"]
C:HS	0	Some concerns	Low risk	No concerns	Major concerns	No concerns	Major concerns	Very low	["Within-study bias","Imprecision","Incoherence"]
C:PS	0	Some concerns	Low risk	No concerns	Major concerns	No concerns	Major concerns	Very low	["Within-study bias","Imprecision","Incoherence"]

S8.4. Stroke

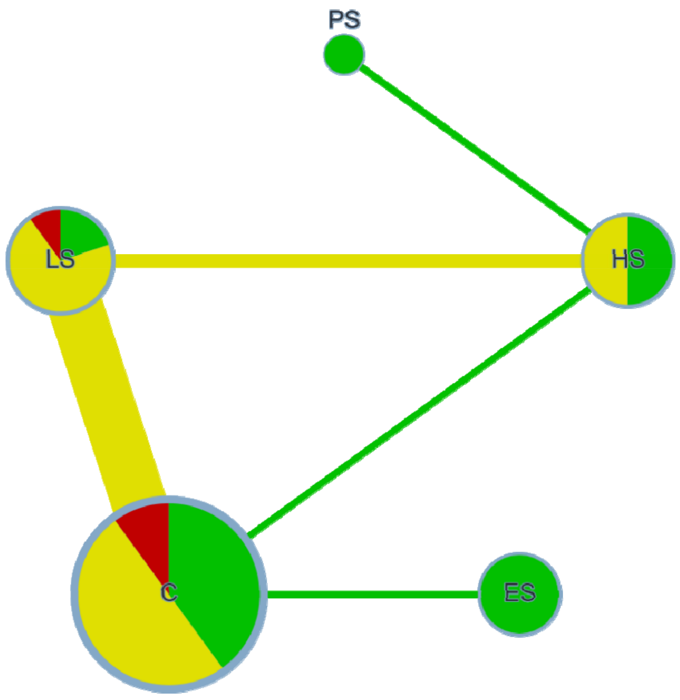

Network plot.

The node size represents sample size; the node color represents the risk of bias; the edge width represents the number of studies; the edge color represents the average Rob.

Per study contribution matrix.

random OR	SHARP 2011	JUPITER 2008	VA-HIT 1999	TING 2012	Fassett 2010	CARDS 2009	4D 2005	STEGMAYR 2005	MEGA 2006	AURORA 2009	ALLIANCE 2004	IDEAL 2005	FOURIER 2017
Mixed estimates	--	--	--	--	--	--	--	--	--	--	--	--	--
C:ES	100.00	0.00	0.00	0.00	0.00	0.00	0.00	0.00	0.00	0.00	0.00	0.00	0.00
C:HS	0.00	34.31	3.73	5.55	0.61	3.78	7.00	0.56	4.62	6.99	12.38	20.46	0.00
C:LS	0.00	4.47	10.34	15.40	1.70	10.49	19.41	1.55	12.80	19.39	1.68	2.78	0.00
Indirect estimates	--	--	--	--	--	--	--	--	--	--	--	--	--
C:PS	0.00	17.16	2.49	3.70	0.41	2.52	4.67	0.37	3.08	4.66	8.26	13.64	39.05

Per comparison contribution matrix.

random OR	C:ES	C:HS	C:LS	HS:LS	HS:PS
Mixed estimates					
C:ES	100.00	0.00	0.00	0.00	0.00
C:HS	0.00	34.31	32.85	32.85	0.00
C:LS	0.00	4.47	91.07	4.47	0.00
Indirect estimates					
C:PS	0.00	17.16	21.90	21.90	39.05

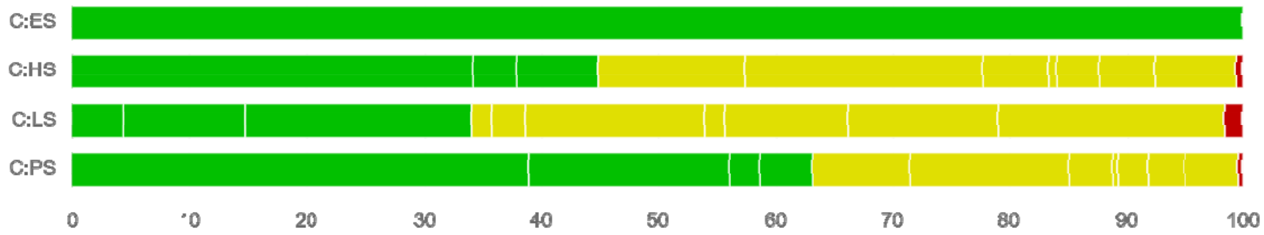

Risk of bias contributions.

The bar chart shows the contributions of each piece of study to the network estimate.

CINeMA quality assessment report.

Comparison	Number of studies	Within-study bias	Reporting bias	Indirectness	Imprecision	Heterogeneity	Incoherence	Confidence rating	Reason(s) for downgrading
C:ES	1	No concerns	Low risk	No concerns	Major concerns	No concerns	No concerns	Low	["Imprecision"]
C:HS	1	Some concerns	Low risk	No concerns	Major concerns	No concerns	No concerns	Low	["Within-study bias","Imprecision"]
C:LS	8	Some concerns	Low risk	No concerns	Major concerns	No concerns	No concerns	Low	["Within-study bias","Imprecision"]
C:PS	0	No concerns	Low risk	No concerns	Major concerns	No concerns	No concerns	Low	["Imprecision"]

S8.5. LDL-C reduction

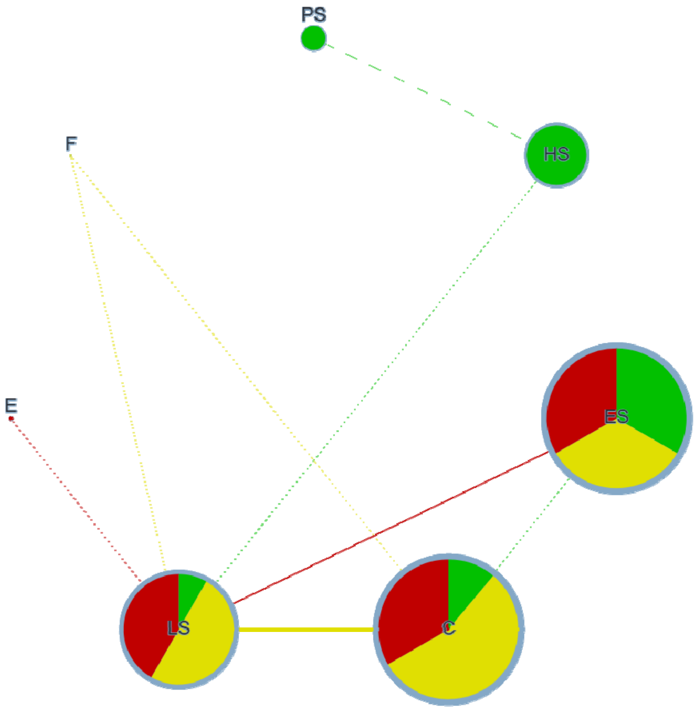

Network plot.

The node size represents sample size; the node color represents the risk of bias; the edge width represents the number of studies; the edge color represents the average Rob.

Per study contribution matrix.

random MD	SHARP 2011	Dogra 2007	ATIC 2007	AURORA 2009	Holmberg 2005	Yasuda 2004	Goicoechea 2006	Verma 2005	Burmeister 2009	ASUCA 2016	Suzuki 2014	IMPROVE-IT 2015	JUPITER 2008	ODYSSE YOUTCOME 2018
Mixed estimates	--	--	--	--	--	--	--	--	--	--	--	--	--	--
C:ES	40.57	5.78	3.60	4.12	3.31	3.40	3.47	3.35	3.26	0.00	13.11	16.03	0.00	0.00
C:F	1.42	77.30	2.92	3.33	2.68	2.75	2.81	2.71	2.64	0.00	0.64	0.78	0.00	0.00
C:LS	4.08	16.10	11.14	12.73	10.23	10.51	10.72	10.35	10.07	0.00	1.83	2.24	0.00	0.00
Indirect estimates	--	--	--	--	--	--	--	--	--	--	--	--	--	--
C:E	2.72	8.93	5.57	6.36	5.11	5.25	5.36	5.17	5.04	47.76	1.22	1.49	0.00	0.00
C:HS	2.72	8.93	5.57	6.36	5.11	5.25	5.36	5.17	5.04	0.00	1.22	1.49	47.76	0.00
C:PS	2.04	6.25	3.71	4.24	3.41	3.50	3.57	3.45	3.36	0.00	0.92	1.12	32.21	32.21

Per comparison contribution matrix.

random MD	C:ES	C:F	C:LS	E:LS	ES:LS	F:LS	HS:LS	HS:PS
Mixed estimates								
C:ES	40.57	1.14	28.00	0.00	29.14	1.14	0.00	0.00
C:F	1.42	50.37	22.68	0.00	1.42	24.10	0.00	0.00
C:LS	4.08	2.65	86.55	0.00	4.08	2.65	0.00	0.00
Indirect estimates								
C:E	2.72	1.77	43.28	47.76	2.72	1.77	0.00	0.00
C:HS	2.72	1.77	43.28	0.00	2.72	1.77	47.76	0.00
C:PS	2.04	1.33	28.85	0.00	2.04	1.33	32.21	32.21

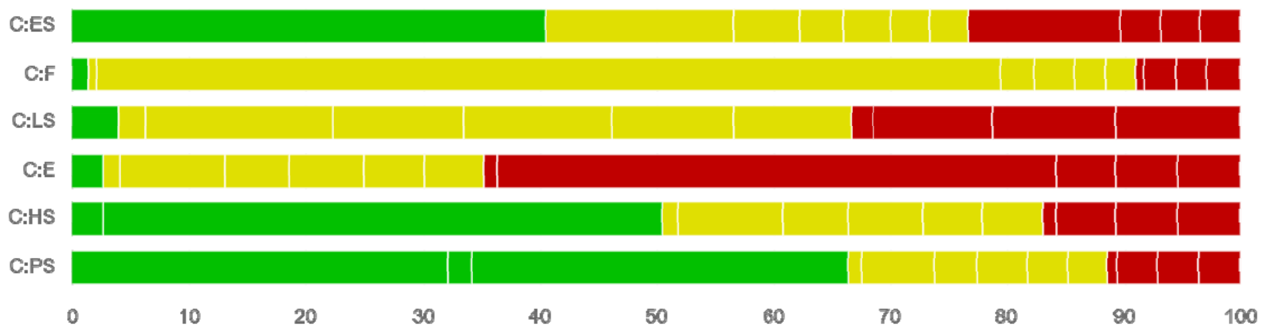

Risk of bias contributions.

The bar chart shows the contributions of each piece of study to the network estimate.

CINeMA quality assessment report.

Comparison	Number of studies	Within-study bias	Reporting bias	Indirectness	Imprecision	Heterogeneity	Incoherence	Confidence rating	Reason(s) for downgrading
C:ES	1	Some concerns	Low risk	No concerns	No concerns	Major concerns	No concerns	Low	["Within-study bias","Heterogeneity"]
C:F	1	Some concerns	Low risk	No concerns	Major concerns	No concerns	Major concerns	Very low	["Within-study bias","Imprecision","Incoherence"]
C:LS	8	Some concerns	Low risk	No concerns	No concerns	Major concerns	No concerns	Low	["Within-study bias","Heterogeneity"]
C:E	0	Major concerns	Low risk	No concerns	Major concerns	No concerns	No concerns	Low	["Within-study bias","Imprecision"]
C:HS	0	Some concerns	Low risk	No concerns	No concerns	No concerns	No concerns	Moderate	["Within-study bias"]
C:PS	0	No concerns	Low risk	No concerns	No concerns	No concerns	No concerns	High	[]
